# Shared molecular features and candidate pathways underlying gastric cancer–depression comorbidity: a systems biology analysis

**DOI:** 10.3389/fbinf.2026.1836419

**Published:** 2026-05-20

**Authors:** Bin Liu, Bowen Hou, Yu Zhao, Jianhua Niu, Jianzhong Hou, Jiageng He, Fang Liu

**Affiliations:** 1 Department of General Surgery, The First Affiliated Hospital of Shihezi University School of Medicine, Shihezi, Xinjiang, China; 2 Shihezi University School of Medicine, Shihezi, Xinjiang, China

**Keywords:** bioinformatics, comorbidity, depression, gastric cancer, miRNA-mRNA network

## Abstract

The bidirectional association between gastric cancer (GC) and depression remains incompletely elucidated. This investigation examines the genetic and molecular correlations between GC and depression through bioinformatics and experimental approaches. Utilizing Gene Expression Omnibus (RRID: SCR_005012), DisGeNET (RRID: SCR_006178), and GeneCards (RRID: SCR_002773) databases, 130 GC-associated and 534 depression-associated genes were identified. Overlapping genes underwent further analysis via Gene Ontology, Kyoto Encyclopedia of Genes and Genomes, and protein-protein interaction network methodologies. Six pivotal hub genes were identified: SERPINE1, COL4A1, PDGFRB, BMP1, NOTCH3, and EDNRA, with SERPINE1 emerging as a particularly significant hub gene. These genes demonstrated upregulation in GC tissues and exhibited correlation with diminished survival outcomes. Furthermore, single-sample Gene Set Enrichment Analysis revealed their association with immune cell infiltration patterns. Additionally, miRNA-mRNA network analysis demonstrated that specific microRNAs, including miR-21-5p, miR-145-5p, miR-16-5p, miR-34a-5p, and miR-491-5p, were predicted to target these genes. Real-time quantitative polymerase chain reaction and Western blot validation subsequently verified the distinct expression patterns of these mRNAs in GC tissues. Critical pathways, encompassing the PI3K-Akt, AGE-RAGE, and proteoglycans pathways, may contribute to the interconnection between GC and depression. These findings illuminate potential molecular linkages between GC and depression, though additional investigation is required to elucidate the underlying mechanisms.

## Graphical Abstract

**Figure:**
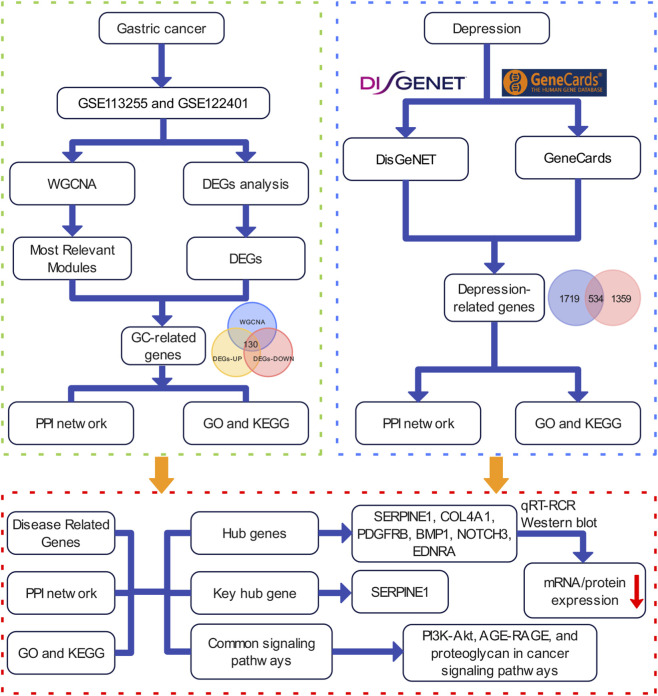

## 1 Introduction

Gastric cancer (GC) represents one of the most prevalent malignancies affecting the digestive tract, constituting a significant threat to human health and longevity (Smyth et al., 2020). Per the Global Cancer Statistics 2022, GC is positioned as the fifth leading cause in terms of both incidence and mortality across 36 categories of malignant neoplasms (Bray et al., 2024). Although global incidence and mortality rates associated with GC have demonstrated a declining trend throughout the preceding decade, elevated case numbers and fatality rates persist within East Asian populations, with China exhibiting a particularly pronounced burden (Sekiguchi et al., 2022; Chen et al., 2016). This regional variation can be attributed to multifactorial interactions encompassing genetic susceptibility, behavioral patterns, environmental determinants, and *Helicobacter pylori* colonization (Nagini, 2012; Tsukamoto et al., 2017). Contemporary treatment modalities, encompassing surgical intervention, radiation therapy, systemic chemotherapy, and precision molecular therapeutics, have yielded enhanced clinical outcomes for selected patient populations (Guan et al., 2023). Nevertheless, suboptimal early diagnostic rates coupled with the emergence of therapeutic resistance during advanced disease stages continue to compromise overall survival (OS) outcomes.

Depression represents a prevalent and debilitating mental disorder, impacting approximately 350 million individuals globally. The condition is distinguished by enduring melancholy and diminished interest, frequently accompanied by cognitive impairment and social isolation, substantially compromising individual quality of life and societal frameworks (Difrancesco et al., 2022). Based on World Health Organization data, in the absence of efficacious intervention strategies, depression is anticipated to emerge as a primary mortality factor worldwide by 2030 (Huang et al., 2023).

Notably, GC, which affects digestive system architecture, and depression, which influences cognitive processes, exhibit an undeniable interconnection (Liu JY. et al., 2023). Epidemiological data indicate that individuals diagnosed with GC demonstrate a 1.28-fold increased likelihood of developing depression relative to those without GC (Kwon et al., 2022). Conversely, patients experiencing depression present a 1.84-fold elevated risk of GC development compared to the general population (Zhang et al., 2021). This reciprocal relationship has attracted substantial investigative focus. Furthermore, Han et al. (2013) documented that variables including reduced income levels, alterations in body perception, fatigue, dyspnea, and sleep disturbances among GC survivors may facilitate depression onset. Conversely, neuroendocrine and inflammatory modifications linked to depression may enhance vulnerability to gastric mucosal damage, consequently increasing malignancy risk (Beurel et al., 2020).

Mechanistically, GC-induced oxidative stress may compromise neurological function through oxidative damage mechanisms, thereby precipitating or aggravating depressive manifestations (Wei et al., 2009). Moreover, serum and tissue concentrations of leptin and its receptor (LepRb) are markedly elevated in GC patients presenting with depression relative to healthy controls and non-depressed patients (Pan et al., 2017). This observation indicates that the leptin-LepRb axis may not only accelerate GC progression but also exacerbate psychological disturbances via central nervous system modulation. In contrast, elevated oxidative stress levels in depressed individuals trigger ABL1 proto-oncogene activation through reactive oxygen species pathways. The ABL1 activation subsequently facilitates GC advancement by modulating downstream signaling cascades (Huang T. et al., 2019). Furthermore, depression induces neuroendocrine phenotypic alterations in GC cells through β2-AR/MACC1 signaling axis activation, mediated by increased plasma catecholamine concentrations, thereby augmenting tumor invasiveness and metastatic potential (Pan et al., 2021). These observations advance the comprehension of comorbidity mechanisms linking GC and depression. Nevertheless, additional research remains necessary to characterize the phenotypic features and shared signaling networks between these conditions, as well as to decipher their genetic underpinnings, which remain insufficiently elucidated.

**FIGURE 1:**
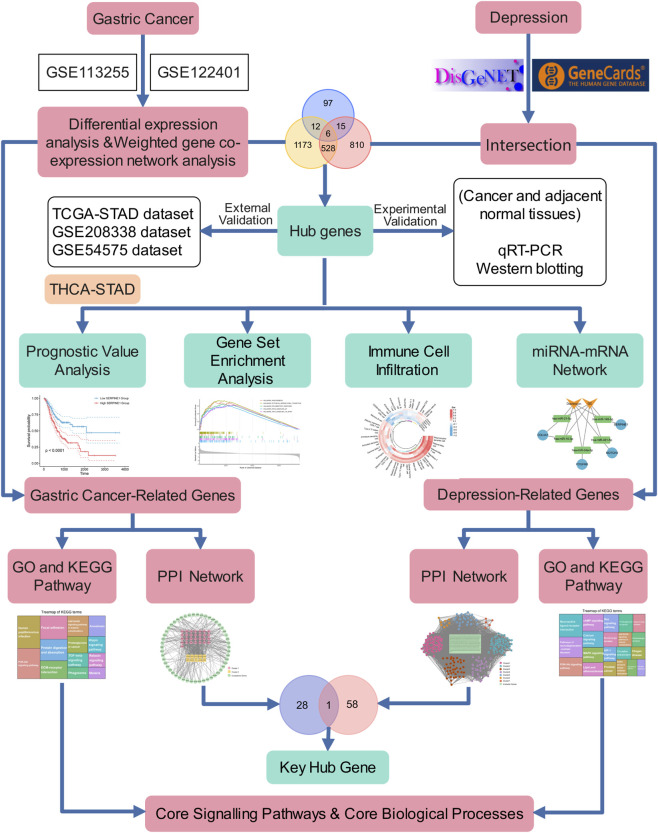
Flowchart of the analysis in this study.

In recent years, the accelerated advancement of bioinformatics and high-throughput omics technologies has transformed the investigation of intricate disease mechanisms. Comprehensive transcriptomic analytical approaches, including RNA-seq and microarray platforms, enable thorough characterization of disease-associated alterations in gene expression patterns. Through the implementation of integrated bioinformatics methodologies, encompassing differential expression analysis, weighted gene co-expression network analysis (WGCNA), and pathway enrichment strategies, investigators can establish effective correlations between genetic perturbations and phenotypic manifestations. For example, Chen Y. et al. (2025) determined CTSK, C3, and IFITM1 as central immune biomarkers for *Helicobacter* pylori-associated GC through the application of differential expression analysis and WGCNA. Wang et al. (2022) employed comprehensive bioinformatics approaches to characterize S100A12, TIGIT, SERPINB2, GRB10, and LHFPL2 as molecular indicators for depression. Furthermore, Hu et al. (2022) applied WGCNA and pathway enrichment analysis to establish that S100A6 and the regulation of endodermal cell fate specification may serve as molecular biomarkers and pathophysiological indicators of type 2 diabetes and pancreatic cancer. Nevertheless, these sophisticated analytical methodologies have not been comprehensively utilized in examining genetic correlations between GC and depression. Therefore, this study aims to predict shared genes and candidate pathways between GC and depression via bioinformatics approaches, providing novel insights into potential molecular associations relevant to their comorbidity. The overall design flowchart for this investigation is presented in Figure 1.

## 2 Materials and methods

### 2.1 Identification of GC-related genes

Microarray expression datasets pertaining to GC were procured from the Gene Expression Omnibus (GEO, RRID: SCR_005012, https://www.ncbi.nlm.nih.gov/geo/, accessed on 6 February 2024) database (Clough et al., 2024). Regarding GC analysis, the GSE113255 and GSE122401 datasets were acquired utilizing the GEOquery (Davis and Meltzer, 2007) R Bioconductor package (RRID:SCR_000146, version 2.70.0, downloaded on 6 February 2024). The GSE113255 dataset (GPL18573 platform, Illumina NextSeq 500, link: https://www.ncbi.nlm.nih.gov/geo/query/acc.cgi?acc=GSE113255) (Kim et al., 2020) encompasses 130 tumor specimens and 10 normal specimens, whereas the GSE122401 dataset (GPL16791 platform, Illumina HiSeq 2,500, link: https://www.ncbi.nlm.nih.gov/geo/query/acc.cgi?acc=GSE122401) (Mun et al., 2019) incorporates 80 tumor specimens and 80 normal specimens. Concerning depression analysis, the GSE208338 and GSE54575 datasets were acquired through the GEOquery Bioconductor R package (downloaded on 20 March 2025). The GSE208338 dataset (GPL5188 platform, [HuEx-1_0-st] Affymetrix Human Exon 1.0 ST Array [probe set (exon) version], link: https://www.ncbi.nlm.nih.gov/geo/query/acc.cgi?acc=GSE208338) (Clough et al., 2024) encompasses gene arrays derived from the dorsolateral prefrontal cortex region, incorporating 24 human patients diagnosed with major depressive disorder (MDD) and 62 control specimens. The GSE54575 dataset (GPL96 platform, [HG-U133A] Affymetrix Human Genome U133A Array, link: https://www.ncbi.nlm.nih.gov/geo/query/acc.cgi?acc=GSE54575) (Chang et al., 2014) comprises gene arrays obtained from the prefrontal cortex, encompassing specimens from 12 human patients with MDD and 12 control specimens. Raw expression matrices underwent preprocessing through the limma package, incorporating normalization and log2 (x+1) transformation procedures. When multiple probes were associated with identical genes, the mean expression value for each gene was computed. Additionally, STAD RNA-seq data [log2 (TPM+1)] and clinical information were procured from The Cancer Genome Atlas (TCGA, RRID:SCR_003193, https://portal.gdc.cancer.gov/, data release 39.0, accessed on 21 February 2024) database (Tomczak et al., 2015) through the TCGABiolinks R package (RRID:SCR_017683, version 2.30.0) (Colaprico et al., 2016). This dataset comprised 412 GC specimens and 36 adjacent normal tissue specimens. In this investigation, the GSE113255 and GSE122401 datasets served as the discovery group, while TCGA-STAD, GSE208338, and GSE54575 functioned as the validation group. Moreover, patients lacking survival information and RNA-seq data were excluded, yielding a final selection of 368 specimens for prognostic evaluation.

To identify genes that assume pivotal roles in GC development, three distinct bioinformatics methodologies were employed for analyzing datasets procured from the GEO database. Initially, the limma R package (RRID:SCR_010943, version 3.64.3) (Ritchie et al., 2015) was employed for expression matrix normalization and identification of differentially expressed genes (DEGs). Significance thresholds were developed at |log2FC| > 1, accompanied by an adjusted p-value <0.05. To ensure analytical robustness, subsequent validation of DEGs was executed employing the GEO2R online tool (RRID:SCR_016569, https://www.ncbi.nlm.nih.gov/geo/geo2r/, accessed on 20 January 2024), employing identical significance criteria of |log2(FC)| > 1 and an adjusted p-value <0.05. Genes were classified as upregulated when log2FC > 1 and downregulated when log2FC < −1. Subsequently, WGCNA was performed utilizing the R package WGCNA (RRID:SCR_003302, version 1.72–1) (Langfelder and Horvath, 2008). The analysis was conducted on the top 5,000 genes ranked by median absolute deviation (MAD) to concentrate on the most variable genetic elements. The WGCNA workflow encompassed several essential steps: (1) sample correlations were calculated using Pearson correlation for sample clustering with outlier exclusion; (2) the optimal soft threshold β was ascertained through the pickSoftThreshold function; (3) an adjacency matrix was constructed employing the formula aij = |Sij|^β, where Sij denotes the Pearson correlation matrix between genes; (4) the adjacency matrix underwent transformation into a topological overlap matrix (TOM) alongside calculation of a dissimilarity matrix for gene module identification; (5) genes were classified into hierarchical clusters based on topological overlap; and (6) correlations between module eigengenes and clinical traits were calculated. In this investigation, emphasis was placed on the gene module demonstrating the strongest correlation with clinical features in the WGCNA results.

Finally, GC-associated genes were determined through a three-step intersection strategy to ensure robustness. First, DEGs identified by limma in GSE113255 and GSE122401 were intersected, with only those genes showing consistent up/downregulation directions in both datasets retained. Second, the aforementioned DEG set was further intersected with DEGs identified by GEO2R to eliminate platform-specific bias. Third, WGCNA was applied to identify key module genes. Ultimately, the GC-related genes were obtained by intersecting and deduplicating the gene sets obtained from the three steps above.

### 2.2 Identification of depression-related genes

Given the absence of microarray datasets for GC patients presenting comorbid depression, depression-associated gene information was obtained from the DisGeNET (RRID:SCR_006178, https://www.disgenet.org/) (Piñero et al., 2020) and GeneCards (RRID:SCR_002773, https://www.genecards.org/) (Stelzer et al., 2016) databases in January 2024. Genes obtained were further filtered based on the relevance score ≥10 for GeneCards and the number of PubMed IDs (PMIDs) > 0 for DisGeNET. To reduce false positives caused by single-database bias and improve the reliability of the candidate gene set, we performed an intersection analysis of the filtered gene lists from the two databases. The gene set obtained after the intersection analysis was defined as depression-related genes.

### 2.3 Functional enrichment analysis and protein‒protein interaction (PPI) network for GC-related and depression-related genes

To annotate gene functions and elucidate participation in signaling pathways, Gene Ontology (GO) and Kyoto Encyclopedia of Genes and Genomes (KEGG) enrichment analyses were executed utilizing the R packages clusterProfiler (RRID:SCR_016884, version 4.10.1) (Yu et al., 2012) and org.Hs.eg.db (RRID:SCR_024739, version 3.18.0) (Carlson et al., 2016). Visualization of results was accomplished through the R package ggplot2 (RRID:SCR_014601, version 3.4.4) (Wickham, 2011). Markedly enriched results were characterized by an adjusted p-value below 0.05. Among the top 20 enriched terms, shared biological processes and pathways between GC and depression were identified and classified as core biological processes and core pathways. Furthermore, PPI networks for GC and depression were procured from the Search Tool for the Retrieval of Interacting Genes (STRING) database (RRID:SCR_005223, https://cn.string-db.org/, version 12.0, accessed on 6 March 2024) (Szklarczyk et al., 2023), with subsequent analysis and visualization performed employing Cytoscape software (RRID:SCR_003032, version 3.10.1).

To identify pivotal modules and genes from the PPI network, three complementary algorithms were implemented via Cytoscape software, with identical parameter settings applied to both the GC and depression PPI networks. First, topological property analysis was performed to calculate the degree, betweenness centrality, and closeness centrality for all nodes, and genes with values exceeding the network average were retained through pre-filtering. Second, the MCODE plugin (Liu L. et al., 2023) was employed for module detection using default parameters (degree cutoff = 2, k-core = 2, node score cutoff = 0.2, haircut = true, fluff = false). The clusters were ranked by their MCODE scores, and genes within the highest-scoring cluster were selected as module core genes. Ultimately, the Maximal Clique Centrality (MCC) algorithm from the Cytohubba plugin was utilized to ascertain the top 20 genes within the network (Chin et al., 2014), which were similarly regarded as closely associated genes. The final single-disease key hub genes were defined as the intersection of the pre-filtered topological genes, MCODE highest-scoring cluster genes, and MCC top 20 genes.

### 2.4 Identification of common hub genes

Through the utilization of the Venn package (version 1.11), overlapping genes derived from the intersection between GC-related genes and depression-related genes were identified as common hub genes. Overlapping genes obtained from the intersection analysis of single-disease key hub genes were identified as the key hub genes of GC and depression.

### 2.5 Enrichment analysis of common hub genes

Gene Set Enrichment Analysis (GSEA) was conducted employing the R package clusterProfiler (RRID:SCR_016884, version 4.10.1) (Yu et al., 2012) to elucidate the functional significance of common hub genes. GSEA represents a sophisticated analytical approach that assesses gene expression data through comparison of specific gene sets against molecular signature databases, thereby yielding insights into biological pathways and processes (Subramanian et al., 2005). The h.all.v2023.2.Hs.symbols.gmt gene set served as the reference gene collection for this investigation. Enrichment results were deemed statistically significant when satisfying the thresholds of adjusted p-value <0.05 and absolute normalized enrichment score >1.

### 2.6 Immune infiltration analysis

Single-sample Gene Set Enrichment Analysis was performed utilizing the R package GSVA (RRID:SCR_021058, version 1.50.0) (Hänzelmann et al., 2013) to investigate the interactions between hub genes and immune cells. Gene sets representing 28 distinct immune cell types were procured from the TISIDB database (http://cis.hku.hk/TISIDB/index.php, accessed 6 December 2023) (Ru et al., 2019). The Wilcoxon rank-sum test was employed to assess differences in the proportions of these immune cell types between GC tissues and adjacent normal tissues, with statistical significance set at p-value <0.05. Spearman correlation analysis was utilized to examine the links between hub genes and the 28 immune cell types.

### 2.7 Construction of miRNAs-mRNAs regulatory networks

miRNAs associated with GC and depression were procured from the Human microRNA Disease Database (HMDD, http://www.cuilab.cn/hmdd, accessed 10 April 2024). The HMDD database consolidates experimental evidence regarding associations between human miRNAs and various diseases (Huang Z. et al., 2019). Through cross-referencing analysis, miRNAs that potentially modulate common hub genes between GC and depression were identified. Subsequently, the miRTarBase database (RRID:SCR_017355, https://miRTarBase.cuhk.edu.cn/, accessed 10 April 2024) (Huang et al., 2020) was employed to predict target genes of these miRNAs. By conducting intersection analysis between predicted target genes and identified common hub genes, specific genes potentially regulated by miRNAs in the context of both diseases were determined. Ultimately, a miRNA-mRNA regulatory network was established and displayed through Cytoscape software.

### 2.8 Clinical specimens and ethics statement

Per the Helsinki Declaration, GC tissues and adjacent nontumour tissues were procured from 12 patients who received surgical intervention at the First Affiliated Hospital of Shihezi University. Approval for the study protocol was sanctioned by the Ethics Committee of the First Affiliated Hospital of Shihezi University. Informed consent was procured from all enrolled patients.

### 2.9 Real-time quantitative polymerase chain reaction (qRT-PCR)

Total RNA isolation from tissue samples was performed employing the E.Z.N.A. Total RNA Kit I (R6834-01, InvitgenOMEGA, United States). RNA concentration was quantified through spectrophotometric analysis, while purity assessment was performed based on the 260 nm/280 nm absorbance ratio. Subsequently, cDNA synthesis was accomplished via reverse transcription employing the RevertAid First Strand cDNA Synthesis Kit (Thermo Fisher Scientific, United States). RT-qPCR amplification was executed using FastSYBR Mixture (Kangwei Biotech, Beijing, China) on a 7,500 Real-Time PCR System (Bio-Rad Laboratories, United States). β-actin served as the internal reference gene. Primers utilized for RT-qPCR were procured from GenePharma (Shanghai, China; Supplementary Table S1).

### 2.10 Western blot analysis

Protein extraction from patient specimens was performed utilizing Radioimmunoprecipitation Assay Lysis Buffer obtained from Sigma-Aldrich. The resultant protein solutions were subjected to sodium dodecyl sulfate-polyacrylamide gel electrophoresis, followed by transfer onto 0.22 μm polyvinylidene difluoride membranes (Millipore Corporation). After blocking with 5% bovine serum albumin (Solarbio), the membranes were subjected to overnight incubation at 4 °C employing primary antibodies targeting BMP1 (AffinityBiosciences, #DF9280, 1:1000), EDNRA (AffinityBiosciences, #DF4923, 1:1000), SERPINE1 (AffinityBiosciences, #BF0086, 1:1000), PDGFRB (AffinityBiosciences, #AF6133, 1:1000), COL4A1 (AffinityBiosciences, #AF0510, 1:1000), NOTCH3 (AffinityBiosciences, #AF7548, 1:1000), and GAPDH (ZSGB-Bio, TA-08, 1:1000). After washing, the membranes received incubation with corresponding secondary antibodies for 2 h under ambient conditions. Detection of protein bands was accomplished using enhanced chemiluminescence reagent (Millipore).

### 2.11 Statistical analysis

All analytical procedures were executed utilizing R software (RRID:SCR_001905, version 4.1.2; https://www.r-project.org/) and GraphPad Prism 8.0 (RRID:SCR_000306). All qRT-PCR and Western blot experiments were performed with three technical replicates per sample. For examination of publicly available datasets retrieved from TCGA and GEO databases, the Mann-Whitney U test was utilized to evaluate gene expression differences between distinct groups. Survival analyses were executed employing the Kaplan-Meier (K-M) methodology, with statistical significance ascertained via the log-rank test. During the experimental validation phase, mRNA expression levels in paired GC tissues were assessed through RT-qPCR quantification. Data are denoted as median values with interquartile ranges, and inter-group comparisons were performed utilizing the nonparametric paired Wilcoxon test. Statistical significance was developed at p-values below 0.05. *P < 0.05, **P < 0.01, ***P < 0.001.

## 3 Results

### 3.1 Identification of GC-related genes

**FIGURE 2:**
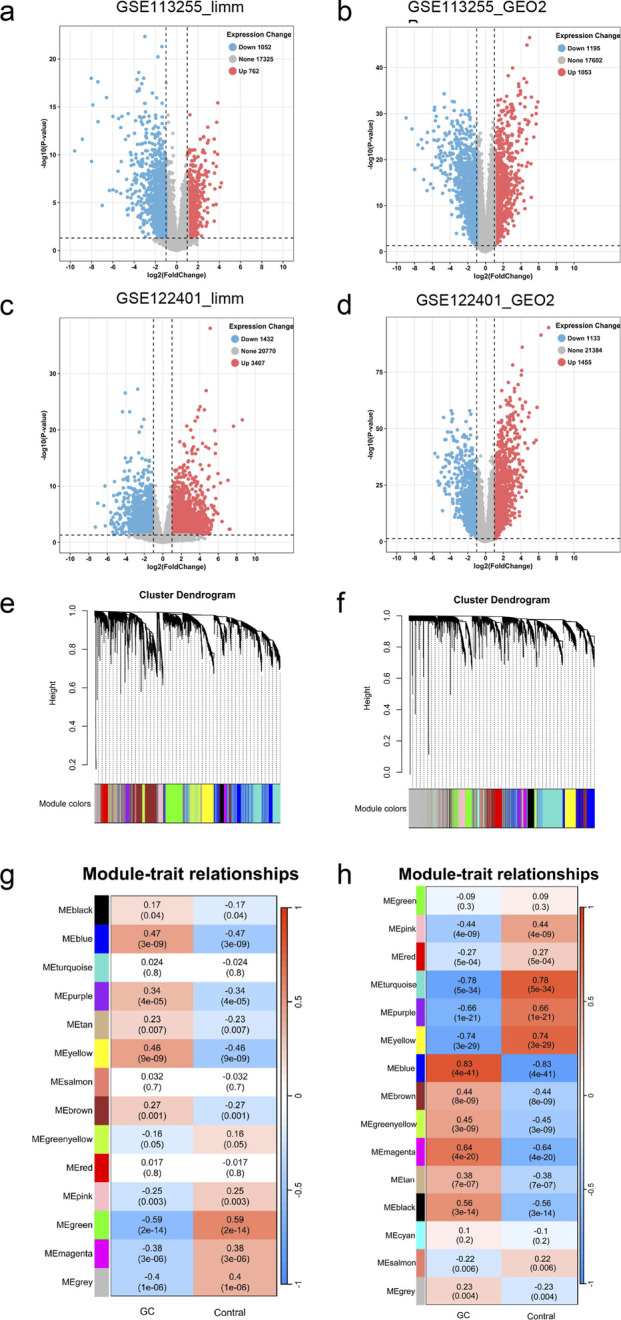
Differential expression analysis and WGCNA of the GEO dataset. **(a)** Volcano plot of DEGs based on limma analysis of the GSE113255 dataset. **(b)** Volcano plot of DEGs based on GEO2R tool analysis of the GSE113255 dataset. **(c)** Volcano plot of DEGs based on limma analysis of the GSE122401 dataset. **(d)** Volcano plot of DEGs based on GEO2R tool analysis of the GSE122401 dataset. **(e, g)** WGCNA results based on GSE113255 dataset. **(f, h)** WGCNA results based on GSE122401 dataset. DEGs, differentially expressed genes; WGCNA, weighted co-expression network analysis.

Through utilization of the limma package, 1814 DEGs were detected within the GSE113255 dataset, wherein 762 genes demonstrated upregulation and 1052 genes exhibited downregulation (Figure 2a). Within the GSE122401 dataset, 2,248 DEGs were identified, encompassing 1053 upregulated genes and 1195 downregulated genes (Figure 2b). Furthermore, through implementation of the GEO2R tool, 4839 DEGs were detected within the GSE113255 dataset, consisting of 3,407 upregulated genes and 1,432 downregulated genes (Figure 2c). Within the GSE122401 dataset, 2,588 DEGs were identified, consisting of 1455 upregulated genes and 1133 downregulated genes (Figure 2d).

To ensure analytical precision in WGCNA implementation, outlier samples were systematically identified and removed before analysis. Pairwise similarities between each sample and all other samples were first calculated using Pearson correlation coefficients. For each sample, a mean similarity score was then computed by averaging its correlation values with all other specimens. The overall mean (μ) and standard deviation (σ) of similarity scores across all samples were determined, and a strict threshold of μ − 3σ was applied to define outliers. Samples with mean similarity scores below this threshold were considered outliers and excluded from downstream analysis. Using this criterion, sample GSM3101199 was excluded from the GSE113255 dataset (Supplementary Figures S1a, b). With soft-thresholding power β established at five and R2 exceeding 0.85 (Supplementary Figure S1c), 14 modules were detected (Figure 2e), while associations between modules and clinical characteristics were elucidated (Figure 2g). Notably, the “blue” module, encompassing 730 genes (cor = 0.47, P = 3e-09), demonstrated the strongest correlation with GC. Therefore, genes contained within this module were selected for subsequent analysis. Similarly, within the GSE122401 dataset, samples GSM3501821, GSM3501830, and GSM3501852 were eliminated (Supplementary Figure S1d,e). With soft-thresholding power β configured at six and R2 surpassing 0.85 (Supplementary Figure S1f), 15 modules were identified (Figure 2f), while relationships between modules and clinical parameters were illustrated (Figure 2h). Among these, the “blue” module, containing 762 genes (cor = 0.83, P = 4e-41), exhibited the strongest correlation with GC. Therefore, attention was focused on genes within this particular module.

**FIGURE 3:**
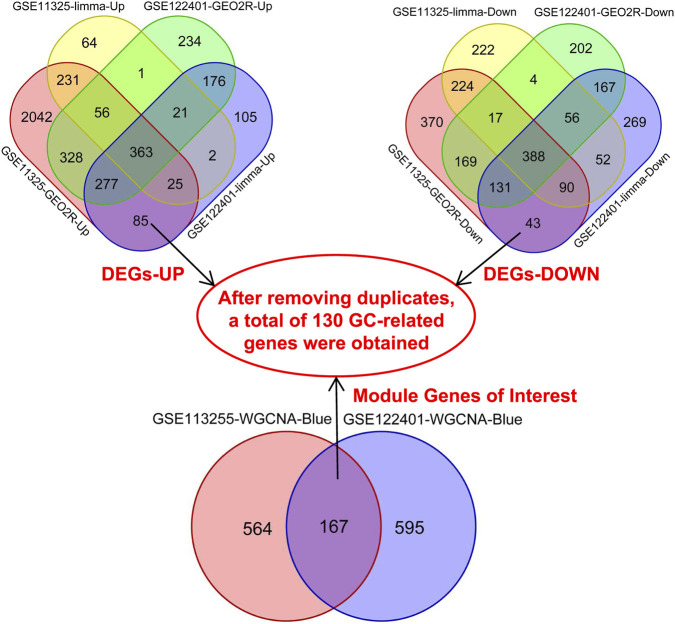
GC-related genes identified by crossing the DEGs and the results of WGCNA in the GSE113255 and GSE122401 datasets. GC, gastric cancer; WGCNA, weighted gene co-expression network analysis; DEGs, differentially expressed genes; DEGs_UP, upregulated differentially expressed genes; DEGs-DOWN, downregulated differentially expressed genes.

Through the intersection of DEGs derived from both datasets, a total of 751 genes demonstrating identical expression patterns were identified, comprising 363 genes exhibiting consistent downregulation and 388 genes displaying consistent upregulation. Additionally, the overlap analysis of genes from the most pertinent modules identified through WGCNA results across both datasets revealed 167 shared genes. Subsequently, following the intersection of these DEGs with the shared modular genes, 130 genes associated with GC were ultimately determined (Figure 3; Supplementary Table S2).

### 3.2 Identification of depression-related genes

A total of 1719 and 1358 depression-related genes were retrieved from the DisGeNET and GeneCards databases, respectively. The intersection of these two gene sets was obtained to generate the final non-redundant depression-related gene set comprising 534 genes.

### 3.3 Functional enrichment analysis and PPI network analysis

**FIGURE 4:**
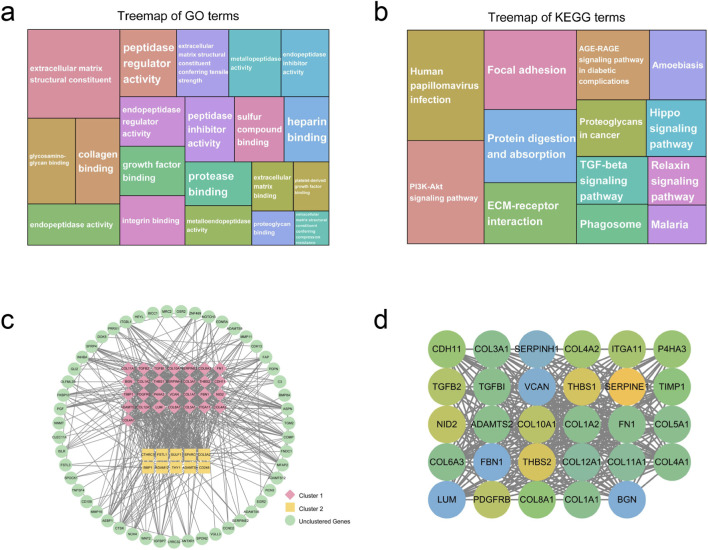
The results of functional enrichment analysis, PPI network, and clustering analysis of GC-related genes. **(a,b)** The top 20 enriched GO **(a)** and KEGG **(b)** terms of GC-related genes. **(c)** PPI network of GC-related genes. **(d)** Top clusters (clustering score = 22.571) extracted from the PPI network of GC-related genes. GC, gastric cancer; PPI, protein‒protein interaction.

Biological process analysis demonstrated that GC-associated genes were predominantly engaged in extracellular matrix (ECM) organization, extracellular structure organization, external encapsulating structure organization, collagen fibril organization, and connective tissue development (Figure 4a; Supplementary Table S4). KEGG enrichment analysis indicated that these genes participate markedly in protein digestion and absorption, ECM-receptor interaction, focal adhesion, human papillomavirus infection, and the AGE-RAGE signaling pathway in diabetic complications (Figure 4b; Supplementary Table S5). Through the establishment of a minimum interaction score at 0.4 and concealment of disconnected nodes, a PPI network comprising 129 nodes and 799 edges was generated (Figure 4c). Topological analysis disclosed that 21 nodes displayed degree, betweenness centrality, and closeness centrality values surpassing the average (Supplementary Table S6). Utilizing the MCODE plugin, the PPI network was partitioned into two principal clusters. The clustering outcomes were subsequently ranked according to scores, with the highest-scoring cluster encompassing 29 nodes and 316 edges, attaining a score of 22.571 (Figure 4d). KEGG enrichment analysis revealed that genes in cluster one showed predominant enrichment in protein digestion and absorption, ECM-receptor interaction, focal adhesion, AGE-RAGE signaling pathway in diabetic complications, and human papillomavirus infection (Supplementary Table S7). Notably, these pathways exhibited substantial overlap with those identified in the initial KEGG analysis of GC-associated genes, indicating that the genes within this cluster may constitute GC-associated genes. Comprehensive results of the remaining clusters are presented in Supplementary Table S8. Additionally, 20 genes were recognized as closely GC-associated genes through utilization of the CytoHubba plugin (Supplementary Figure S2a).

**FIGURE 5:**
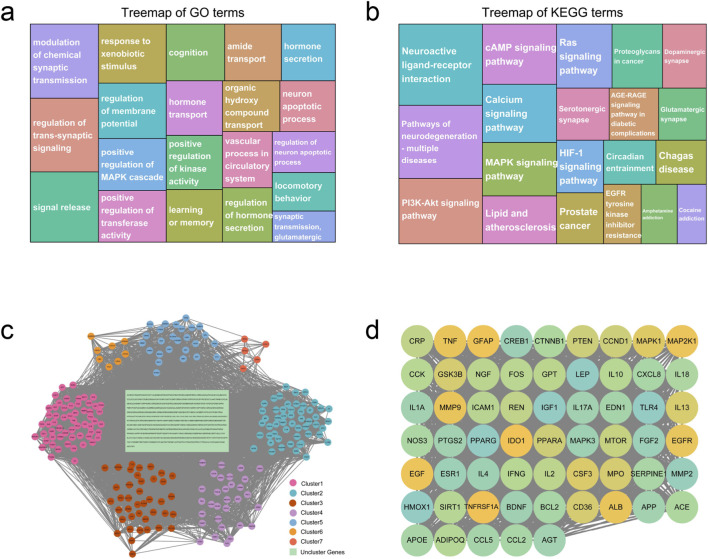
The results of functional enrichment analysis, PPI network, and clustering analysis of depression-related genes. **(a,b)** The top 20 enriched GO **(a)** and KEGG **(b)** terms of depression-related genes. **(c)** PPI network of depression-related genes. **(d)** Top clusters (clustering score = 49.103) extracted from the PPI network of depression-related genes. PPI, protein‒protein interaction.

Genes linked to depression demonstrated enrichment in multiple biological processes, encompassing the regulation of chemical synaptic transmission, control of trans-synaptic signaling, signal release mechanisms, responses to xenobiotic stimuli, and cognitive functions (Figure 5a; Supplementary Table S9). Additionally, KEGG pathway analysis revealed the involvement of depression-associated genes in neuroactive ligand-receptor interactions, cAMP signaling cascades, PI3K-Akt signaling networks, neurodegeneration pathways affecting multiple diseases, and calcium signaling mechanisms (Figure 5b; Supplementary Table S10). Through the establishment of a minimum interaction score threshold of 0.4 and concealment of disconnected nodes, a PPI network comprising 533 nodes and 13,644 edges was generated (Figure 5c). Topological examination indicated that 126 nodes demonstrated degree, betweenness centrality, and closeness centrality values surpassing the mean threshold (Supplementary Table S11). Concurrently, utilizing the MCODE plugin, the network underwent further clustering into 19 discrete clusters, wherein the highest-scoring cluster attained a score of 49.103 while incorporating 59 nodes and 1,424 edges (Figure 5d). Clustering outcomes with scores exceeding five are documented in Supplementary Table S12. KEGG enrichment analysis disclosed that genes within cluster one participate in the IL-17 signaling cascade, prostate cancer pathogenesis, AGE-RAGE signaling networks in diabetic complications, Chagas disease mechanisms, and HIF-1 signaling pathways. Notably, these pathways exhibited substantial concordance with those identified through initial KEGG analysis of depression-associated genes, indicating that genes within this cluster may constitute depression-related genetic elements (Supplementary Table S13). Moreover, 20 genes were identified as closely depression-associated genes through application of the CytoHubba plugin (Supplementary Figure S2b).

### 3.4 Identification of overlapping signaling pathways and key hub genes

**FIGURE 6:**
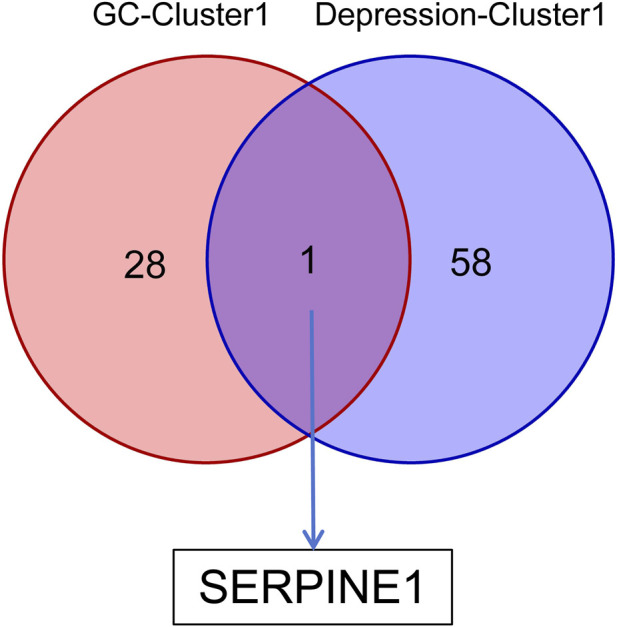
Key hub genes of GC and depression. GC, gastric cancer.

Intersection analysis of KEGG pathways demonstrated shared signaling pathways between GC and depression, encompassing the PI3K-Akt signaling pathway, the AGE-RAGE signaling pathway in diabetic complications, and proteoglycans in cancer. Additionally, SERPINE1 was identified as the only key hub gene for both diseases through the intersection of three complementary PPI-based approaches (topological property analysis, MCODE, and MCC algorithm) applied separately to GC and depression PPI networks (Figure 6).

### 3.5 Identification of hub genes and analysis of prognostic value

**FIGURE 7:**
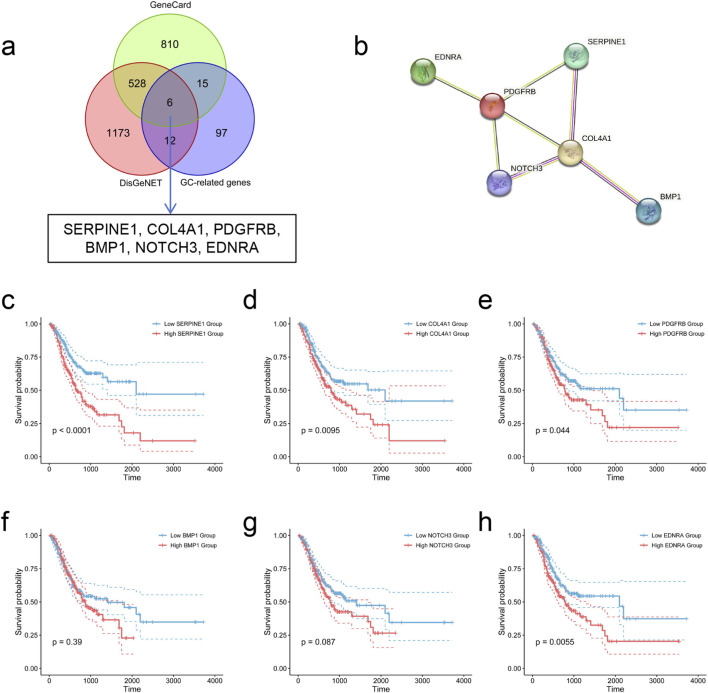
Identification and analysis of hub genes bridging GC and depression. **(a)** Six hub genes bridging GC and depression. **(b)** PPI network of six hub genes. **(c–h)** Kaplan-Meier survival curves based on the median expression levels of SERPINE1 **(c)**, COL4A1 **(d)**, PDGFRB **(e)**, BMP1 **(f)**, NOTCH3 **(g)**, and EDNRA **(h)** in the TCGA-STAD dataset. GC, gastric cancer.

Through the intersection of GC-associated genes with depression-associated genes utilizing the Venn package (version 1.11), six overlapping genes were identified between GC and depression: SERPINE1, COL4A1, PDGFRB, BMP1, NOTCH3, and EDNRA (Figure 7a). These genes, designated as hub genes, are hypothesized to function as molecular bridges linking GC and depression. The PPI network analysis pertaining to these six genes is presented in Figure 7b.

To assess the prognostic value of the hub genes in GC, Kaplan-Meier survival analysis was performed on the STAD dataset employing the survival R package. Samples exhibiting follow-up periods shorter than 30 days were eliminated, while gene expression levels were split into elevated and reduced expression groups per the median threshold. The K-M curves demonstrated that elevated expression of SERPINE1, COL4A1, PDGFRB, and EDNRA was markedly linked to inferior OS (P < 0.05) (Figures 7c–h). These observations were subsequently corroborated through the GEPIA2 online platform (http://gepia2.cancer-pku.cn/#survival, accessed on 1 October 2024) (Supplementary Figures S3a–f).

### 3.6 GSEA enrichment analysis of hub genes

**FIGURE 8:**
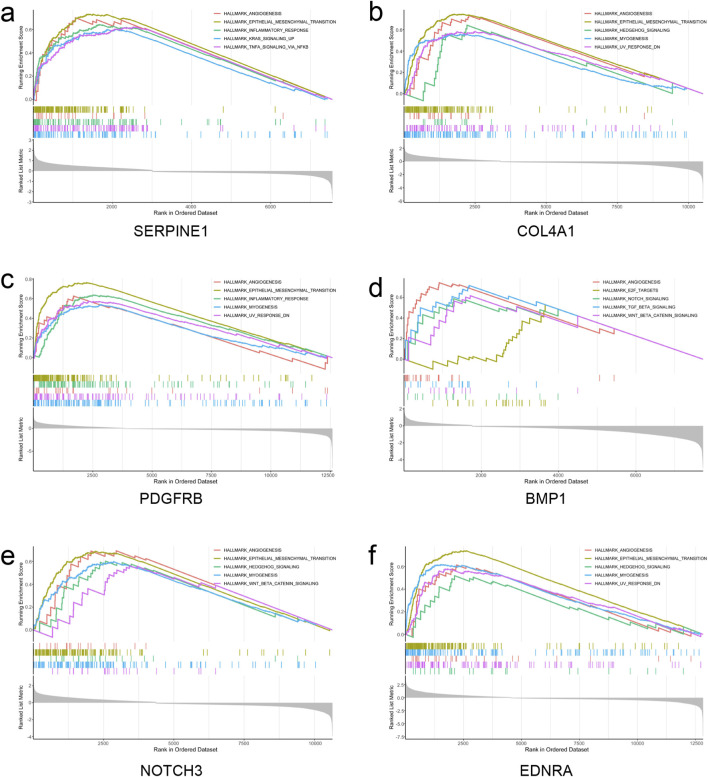
Top 5 GSEA results of hub genes based on the TCGA-STAD dataset. **(a)** GSEA results of SERPINE1. **(b)** GSEA results of COL4A1. **(c)** GSEA results of PDGFRB. **(d)** GSEA results of BMP1. **(e)** GSEA results of NOTCH3. **(f)** GSEA results of EDNRA. GSEA, gene set enrichment analysis.

Samples within the STAD dataset were stratified into high and low expression groups per the median expression values of the hub genes. GSEA analysis was conducted for each gene utilizing the clusterProfiler R package, with the five most markedly enriched pathways being selected for demonstration. The analysis revealed that SERPINE1 and PDGFR genes were implicated in pathways including HALLMARK_EPITHELIAL_MESENCHYMAL_TRANSITION, HALLMARK_INFLAMMATORY_RESPONSE, and HALLMARK_ANGIOGENESIS. COL4A1 and NOTCH3 genes were observed to engage in HALLMARK_EPITHELIAL_MESENCHYMAL_TRANSITION, HALLMARK_ANGIOGENESIS, and HALLMARK_HEDGEHOG_SIGNALING pathways. BMP1 gene participation was identified in HALLMARK_ANGIOGENESIS, HALLMARK_TGF_BETA_SIGNALING, and HALLMARK_WNT_BETA_CATENIN_SIGNALING pathways, while EDNRA gene involvement was detected in HALLMARK_EPITHELIAL_MESENCHYMAL_TRANSITION, HALLMARK_MYOGENESIS, and HALLMARK_ANGIOGENESIS pathways. Collectively, these genes were predominantly linked to epithelial-mesenchymal transition, angiogenesis, and inflammatory response processes (Figures 8a–f). Additionally, comparable findings were documented in datasets GSE113255 (Supplementary Figures S4a–f) and GSE122401 (Supplementary Figures S5a–f).

### 3.7 Immune infiltration analysis and the association between hub genes and immune cells

To explore the shared immune mechanisms underlying the comorbidity of GC and depression, and to elucidate the regulatory roles of the six hub genes in the common immune microenvironment, we performed ssGSEA. This analysis compared immune cell infiltration patterns between GC and depression and assessed their correlations with the hub genes.

**FIGURE 9:**
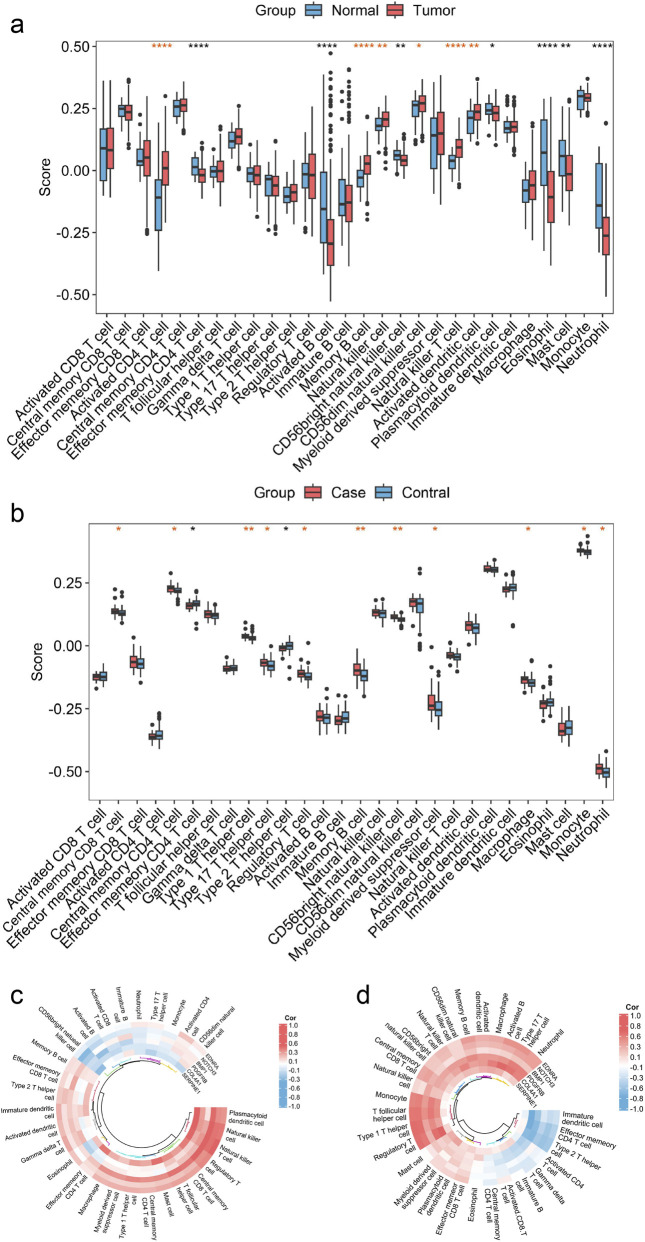
ssGSEA results of the hub genes based on the TCGA-STAD dataset. **(a)** Differences in immune cells between GC and adjacent normal tissues in TCGA-STAD dataset. **(b)** Differences in immune cells infiltration between depression and healthy controls in GSE208338 dataset. **(c)** Correlation heatmap between immune cell infiltration and six hub genes in TCGA-STAD dataset. **(d)** Correlation heatmap between immune cell infiltration and six hub genes in GSE208338 dataset. ssGSEA, single-sample gene set enrichment analysis; GC, gastric cancer. The *P* value were shown as *P < 0.05; **P < 0.01; and ***P < 0.001.

Box plot analysis of the GC dataset (TCGA-STAD) revealed markedly elevated levels of activated CD4 T cells, memory B cells, natural killer cells, CD56dim natural killer cells, natural killer T cells, and activated dendritic cells within GC specimens relative to normal tissue samples. In contrast, effector memory CD4 T cells, activated B cells, CD56bright natural killer cells, plasmacytoid dendritic cells, eosinophils, mast cells, and neutrophils displayed substantially diminished abundance (Figure 9a). Examination of the depression dataset (GSE208338) revealed markedly augmented populations of central memory CD8 T cells, central memory CD4 T cells, type 1 helper T cells, type 17 helper T cells, regulatory T cells, memory B cells, CD56bright natural killer cells, myeloid-derived suppressor cells, macrophages, monocytes, and neutrophils within cerebral tissue specimens from depression subjects. Conversely, diminished populations of effector memory CD4 T cells and type 2 helper T cells were observed (Figure 9b). Notably, GC and depression manifested analogous patterns regarding memory B cells, CD56dim natural killer cells, and effector memory CD4 T cells when contrasted with the control group.

Furthermore, Spearman correlation analysis was utilized to investigate the links between hub genes and immune cells in both GC and depression. The correlation heatmaps demonstrate the associations between six hub genes and immune cells in GC tissue and brain tissue of depressed patients, respectively (Figures 9c,d). Notably, substantially similar positive associations were identified for central memory CD8 T cells, regulatory T cells, and T follicular helper cells with SERPINE1, natural killer T cells with BMP1, regulatory T cells with COL4A1, mast cells with EDNRA, and natural killer cells, T follicular helper cells, and type 1 T helper cells with PDGFRB in GC and depression (cor >0.3, P < 0.05). Additionally, similar immune infiltration differences and associations were observed in the GC (GSE113255 and GSE122401) and depression (GSE54575) datasets (Supplementary Figures S6a–f).

### 3.8 Construction of miRNA‒mRNA networks in GC and depression

**FIGURE 10:**
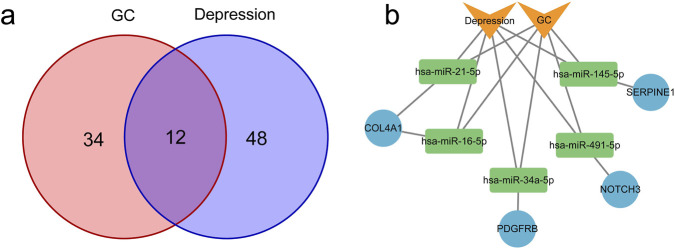
Construction of miRNAs-mRNAs regulatory networks for hub genes. **(a)** Common miRNAs between GC and depression. **(b)** miRNA‒mRNA regulatory network. The orange quadrangles represent GC and depression, the blue circles represent hub genes, and the green rectangles represent common miRNAs. GC, gastric cancer.

Through interrogation of the HMDD database, 45 miRNAs associated with GC and 60 miRNAs correlated with depression were identified. Notably, 12 miRNAs were determined to be shared between both GC and depression (Figure 10a). Subsequently, 4,861 target genes for these 12 shared miRNAs were predicted utilizing the miRTarBase database. Among the predicted target genes, four were recognized as overlapping genes: SERPINE1, COL4A1, PDGFRB, and NOTCH3. Ultimately, the miRNA-mRNA interaction network was developed employing Cytoscape software (Figure 10b). It should be noted that these predictions require further validation by expression correlation analysis and functional experiments.

### 3.9 Internal validation

**FIGURE 11:**
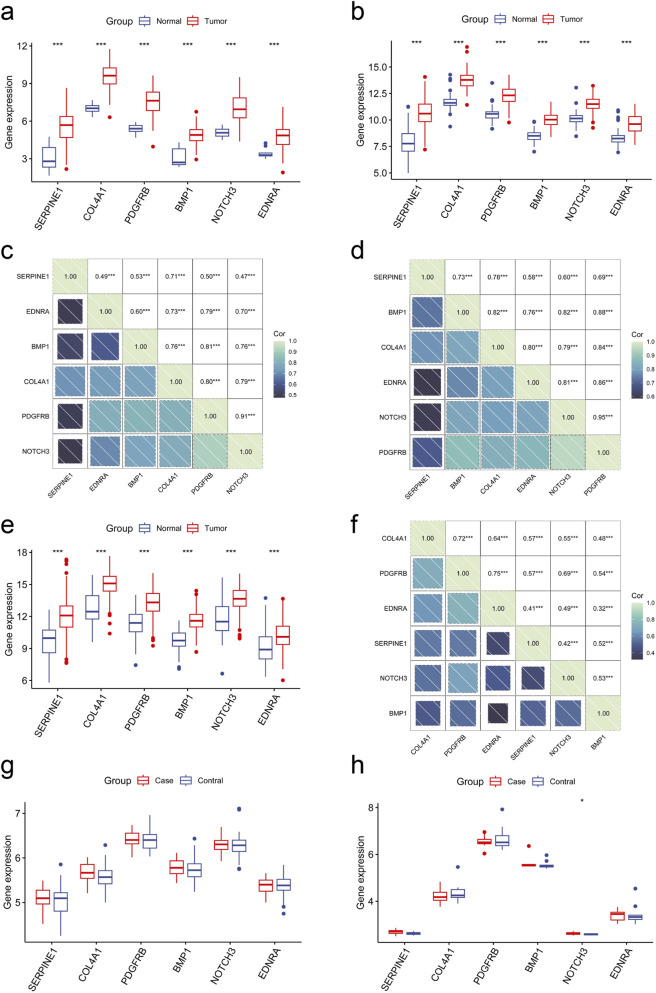
The mRNA levels and correlation analysis of hub genes. **(a,b)** The mRNA expression levels of six hub genes in GSE113255 **(a)** and GSE122401 **(b)** datasets. **(c,d)** Correlation analysis of six hub genes in GSE113255 **(c)** and GSE122401 **(d)** datasets. **(e)** The mRNA levels of six hub genes in the TCGA-STAD dataset. **(f)** Correlation analysis of six hub genes in TCGA-STAD dataset. **(g,h)** The mRNA levels of six hub genes in GSE208338 **(g)** and GSE54575 **(h)** datasets. The *P* value were shown as *P < 0.05; **P < 0.01; and ***P < 0.001.

Based on the gene expression profiles within datasets GSE113255 and GSE122401, elevated expression levels of SERPINE1, COL4A1, PDGFRB, BMP1, NOTCH3, and EDNRA were observed in GC specimens relative to control samples (Figures 11a,b). Correlation analyses revealed statistically significant associations among these six hub genes across both GSE113255 and GSE122401 datasets (cor >0.3, P < 0.05) (Figures 11c,d).

### 3.10 External validation

Based on expression values within the TCGA-STAD dataset, SERPINE1, COL4A1, PDGFRB, BMP1, NOTCH3, and EDNRA demonstrated elevated expression levels in GC relative to control specimens (Figure 11e). Correlation analysis revealed significant inter-correlations among these six hub genes within this group (cor >0.3, P < 0.05) (Figure 11f). Box plot analyses of expression values derived from depression datasets GSE208338 and GSE54575 indicated that NOTCH3 exhibited significant upregulation in depression relative to normal control subjects (P < 0.05) (Figures 11g,h). Notably, expression variations of SERPINE1, COL4A1, PDGFRB, BMP1, and EDNRA within the depression datasets failed to achieve statistical significance.

Box plot analyses of expression values derived from depression datasets GSE208338 and GSE54575 were performed to validate the expression of the six hub genes in MDD. In the GSE208338 dataset, none of the six hub genes (SERPINE1, COL4A1, PDGFRB, BMP1, NOTCH3, and EDNRA) showed statistically significant differential expression between MDD patients and healthy controls (P > 0.05) (Figure 11g). In the GSE54575 dataset only NOTCH3 exhibited significant upregulation in MDD relative to normal control subjects (P < 0.05), while SERPINE1, COL4A1, PDGFRB, BMP1, and EDNRA still showed no statistically significant expression differences (P > 0.05) (Figure 11h). This constraint may be attributed to multiple factors. Initially, the GSE54575 dataset encompasses merely 12 MDD specimens, substantially limiting the capacity to identify meaningful variations. Additionally, both MDD datasets were derived from prefrontal cortex subregions. Contemporary neuroimaging investigations have demonstrated that neuropathological alterations in MDD display substantial regional specificity, indicating that molecular modifications within particular subregions may become masked when examined at the comprehensive cortical level (Tannous et al., 2020; Jacob et al., 2022).

### 3.11 Experimental validation

**FIGURE 12:**
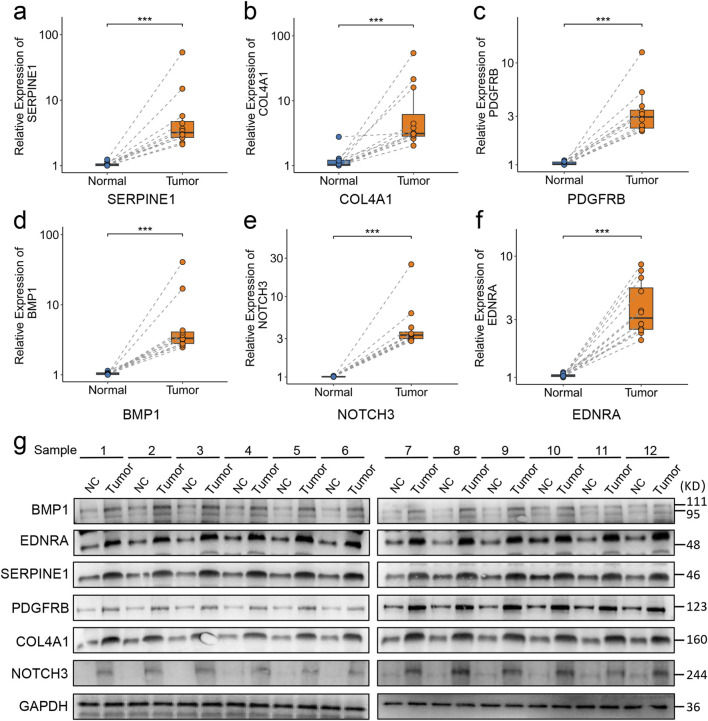
RT-qPCR and Western blot analysis of mRNA and protein expression levels of hub genes in GC. **(a–f)** RT-qPCR quantification of SERPINE1 **(a)**, COL4A1 **(b)**, PDGFRB **(c)**, BMP1 **(d)**, NOTCH3 **(e)**, and EDNRA **(f)** in paired GC and adjacent nontumor tissues. **(g)** Western blot analysis of protein expression in paired GC and adjacent nontumor tissues. GC, gastric cancer. The *P* value were shown as *P < 0.05; **P < 0.01; and ***P < 0.001.

To further validate the expression patterns of SERPINE1, COL4A1, PDGFRB, BMP1, NOTCH3, and EDNRA in malignant tissues, gene expression levels were assessed in 12 GC specimens alongside corresponding adjacent tissues through RT-qPCR and Western blot analyses. The findings demonstrated that SERPINE1, COL4A1, PDGFRB, BMP1, NOTCH3, and EDNRA exhibited markedly elevated expression levels in GC tissues relative to paired normal tissues (Figures 12a–g).

## 4 Discussions

GC and depression, notwithstanding their distinct pathological mechanisms and physiological processes, are increasingly acknowledged to possess an intrinsic association. For example, Zhou et al. (Zhou and Yu, 2022) demonstrated that particular immune cells, encompassing Th1, Th2, and Th17 cells, along with their corresponding cytokines, are intimately linked to emotional and cognitive impairments in GC patients. Specific genetic markers, encompassing FKBP5 (Kang JI. et al., 2012) and APEX1 (Wei et al., 2009), may serve as pivotal mediators in the interaction between GC and depression. Liu et al. (Liu Y. et al., 2022) established that β2-adrenergic receptor activation triggers epithelial-mesenchymal transition in GC cells via the JAK-STAT3 signaling pathway, a mechanism concurrently implicated in depressive symptoms observed in GC patients. Additionally, α-2-macroglobulin concentrations following abdominal surgical procedures in cancer patients have been correlated with depression severity (Fujita et al., 2003). Nevertheless, the molecular mechanisms governing the complex interplay between GC and depression remain incompletely elucidated. To the best of current knowledge, no investigation has comprehensively examined the potential genetic markers and molecular pathways implicated. Therefore, this study elucidated the bidirectional genes and signaling pathways linking GC and depression through bioinformatics approaches, offering a novel perspective on their comorbidity mechanisms.

In this investigation, a combination of the limma R package, the GEO2R tool, and WGCNA was utilized to validate the presence of 130 GC-related genes. Through the synthesis of findings derived from the DisGeNET and GeneCards databases, a total of 534 depression-related genes were identified. GO enrichment analysis suggested that GC-related genes are predominantly engaged in the regulation of ECM structure and function. These processes may undergo disruption or alteration during tumor formation and progression, subsequently facilitating the invasion and metastasis of malignant cells. Conversely, depression-related genes exert influence on signaling and cognitive processes within the nervous system. Perturbations in these processes can precipitate alterations in emotional regulation and cognitive function. Additionally, KEGG enrichment analysis demonstrated that GC-related genes participated in several biological pathways, encompassing hsa04974 (protein digestion and absorption), hsa04512 (ECM-receptor interaction), hsa04510 (focal adhesion), hsa05165 (human papillomavirus infection), and hsa04933 (AGE-RAGE signaling pathway in diabetic complications), consistent with preceding research. Furthermore, depression-related genes were predominantly enriched in pathways including hsa04080 (neuroactive ligand-receptor interaction), hsa04024 (cAMP signaling pathway), hsa04151 (PI3K–Akt signaling pathway), hsa05022 (pathways of neurodegeneration–multiple diseases), and hsa04020 (calcium signaling pathway). Notably, it was observed that the PI3K-Akt signaling pathway, the AGE-RAGE signaling pathway in diabetic complications, and proteoglycans in cancer pathways were commonly enriched in both diseases. While this observation suggests that these pathways may play a role in the developmental mechanisms underlying their comorbidity, it is important to acknowledge that these pathways are broadly involved in multiple diseases and are commonly enriched in general pathological processes such as inflammation and cell proliferation. Thus, their enrichment in both GC and depression may reflect general pathological responses rather than specific, unique mechanistic links exclusively underlying GC-depression comorbidity.

The PI3K-Akt signaling pathway, the AGE-RAGE signaling pathway in diabetic complications, and proteoglycans in cancer pathways have been established as markedly linked to GC occurrence and progression (Fattahi et al., 2020; Yan et al., 2018; Zhao et al., 2021). Conversely, within the depression context, the PI3K-Akt pathway modulates mood and behavioral patterns through its effects on neuronal survival, plasticity, and neurotransmitter system functionality (Li et al., 2021). AGE-RAGE pathway activation correlates with oxidative stress and inflammatory responses, while its inhibition has been demonstrated to ameliorate depressive-like behaviors (Yu et al., 2022). Additionally, the proteoglycan signaling pathway serves an essential function in preserving ECM structure and functionality (Dituri et al., 2022). Investigations reveal that ECM modifications can alter neural connectivity and neurotransmitter release within the brain, consequently affecting mood regulation and stress response mechanisms (Blanco and Conant, 2021). Collectively, these signaling pathways represent fundamental components in the pathogenesis of both GC and depression, but their broad involvement in diverse pathological conditions limits the specificity of their implication in GC-depression comorbidity.

Notably, the genes SERPINE1, COL4A1, PDGFRB, BMP1, NOTCH3, and EDNRA have been characterized as hub genes establishing connections between GC and depression. Among these, SERPINE1 has been recognized as a pivotal hub gene that may exert a fundamental role in the pathogenesis and progression of these pathological conditions. The SERPINE1 gene encodes plasminogen activator inhibitor 1 (PAI-1), which functions to inhibit tissue plasminogen activator (t-PA) and urokinase-type plasminogen activator, consequently diminishing plasmin formation and modulating fibrin degradation (Rabieian et al., 2018). Clinical investigations demonstrate that SERPINE1 expression levels are markedly upregulated in GC tissue and exhibit strong correlations with tumor malignancy and prognosis (Brungs et al., 2017). Subsequent research has established that SERPINE1 facilitates tumor growth through enhanced angiogenesis within GC tissues (Teng et al., 2021). Furthermore, in the immunological microenvironment of GC, SERPINE1 modulates the function and infiltration of immune cells, consequently impacting tumor growth and metastasis (Khan et al., 2022; Feng et al., 2023). In the context of depression, dysregulation of the fibrinolytic system may constitute a fundamental mechanism resulting in compromised brain remodeling, encompassing thrombus formation, enhanced inflammatory responses, and deterioration of neuronal connections (Hoirisch-Clapauch, 2022). Scientific evidence demonstrates that t-PA can facilitate the conversion of proBDNF into mature BDNF through a proteolytic cascade, whereas depression is characterized by decreased BDNF levels, thereby strengthening the association between the fibrinolytic system and depression (Tsai, 2006; Tsai, 2007). An additional investigation revealed substantial elevation in serum PAI-1 levels among patients with major depression, emphasizing the potentially significant role of SERPINE1 in the association between GC and depression (Elsayed et al., 2023). Additionally, in accordance with prior research, it was also observed that the genes COL4A1, PDGFRB, BMP1, NOTCH3, and EDNRA exhibit overexpression in GC, which may contribute to unfavorable prognosis in patients (Wang et al., 2021; Liu B. et al., 2022; Lee et al., 2022; Kang H. et al., 2012; Wei et al., 2018). Regarding depression, scientific evidence indicates that the upregulation of the COL4A1 gene in the dentate gyrus (DG) of MDD mouse brains suggests that this gene may participate in depression development (Wei et al., 2021). The PDGFRB gene encodes a transmembrane protein functioning as a receptor tyrosine kinase that undergoes autophosphorylation and activates its tyrosine kinase activity upon PDGF binding. Activated PDGFRB subsequently stimulates downstream signaling pathways, including the RAS-MAPK pathway and the PI3K-Akt pathway, thereby modulating cell proliferation, migration, and survival (Iesato et al., 2021; Sun et al., 2021). Scientific evidence suggests that abnormal activation of the immune-inflammatory response system occurs in patients with MDD, accompanied by elevated PDGF levels (Maes et al., 2022). Furthermore, a substantial increase in PDGFRB in the serum of MDD patients could potentially intensify the inflammatory response (Al-Hakeim et al., 2023). The BMP1 gene is predominantly involved in ECM remodeling processes, particularly in collagen maturation and mineralization mechanisms (Sarras, 1996). ECM remodeling is essential for nervous system function and plasticity and can contribute to or aggravate the development of depressive symptoms (Spijker et al., 2020). Additional evidence suggests that the perineuronal net, a specialized form of ECM, is intimately associated with depression (Morphett et al., 2024). Animal investigations have demonstrated that electrostimulation can restructure the ECM in depressed mice, enhancing synaptic plasticity and producing antidepressant effects (Feng et al., 2022). Mutations in the NOTCH3 gene are acknowledged as classical genetic markers of cerebral autosomal dominant arteriopathy with subcortical infarcts and leukoencephalopathy (CADASIL) (Park et al., 2017). Nevertheless, the relationship between this gene and depression remains ambiguous. Research demonstrates that the NOTCH signaling pathway maintains a persistent and substantial role in promoting neurogenesis and remodeling (Ables et al., 2011). Ma et al. (Ma et al., 2019) reported that silencing miR-9 in the hippocampus can effectively stimulate the Notch signaling pathway and enhance the regeneration of hippocampal neurons, thereby ameliorating depressive symptoms in mice. The EDNRA gene encodes a G protein-coupled receptor belonging to the endothelin receptor family and participates in mammalian circulatory development and neuronal differentiation (Lee et al., 2023). However, the relationship between EDNRA and depression remains inadequately characterized and necessitates further investigation. In conclusion, it is postulated that SERPINE1, COL4A1, PDGFRB, BMP1, NOTCH3, and EDNRA may function as novel molecular markers establishing connections between GC and depression. Notably, only NOTCH3 showed statistically significant upregulation in MDD datasets, while the other five hub genes exhibited non-significant expression differences. This discrepancy does not negate their potential roles in GC-depression comorbidity, but stems from multiple objective limitations of the current datasets. First, the small sample sizes of depression cohorts severely limit statistical power to detect subtle but biologically meaningful expression changes. Second, both datasets were derived exclusively from the prefrontal cortex, whereas MDD pathogenesis involves multiple brain regions, which may be masked in bulk prefrontal cortex analysis. Third, the clinical heterogeneity of MDD (distinct subtypes, disease severities and durations) and the terminal nature of post-mortem brain samples may dilute true expression signals. Finally, these genes may mediate comorbidity through peripheral pathways (e.g., systemic inflammation, neuroendocrine regulation) rather than direct central expression changes, which would not be detected in brain tissue analyses. Nevertheless, this conclusion is predominantly derived from bioinformatics analysis with limited experimental validation. Future investigations should incorporate larger clinical groups, functional experiments, and mechanistic studies to validate the causal roles of these key genes and pathways in both conditions.

miRNA is a small non-coding RNA molecule that plays a crucial role in post-transcriptional gene regulation by specifically binding to the 3′untranslated region (3′UTR) of target mRNA, thereby inhibiting its translation or promoting its degradation. In this study, we identified 12 miRNAs shared between gastric cancer and depression using the HMDD database. Among them, miR-21-5p, miR-145-5p, miR-16-5p, miR-34a-5p, and miR-491-5p were predicted to target and regulate the hub genes SERPINE1, COL4A1, PDGFRB, and NOTCH3. These regulatory factors may exert their effects by modulating common signaling pathways or genes involved in both GC and depression. Accumulating evidence has demonstrated the functional roles of these miRNAs in both diseases. In GC, upregulation of miR-21-5p has been shown to promote lymph node metastasis and correlate with poor patient prognosis (Ciesielski et al., 2025), while intervention with anti-miR-21-5p effectively inhibits the metastatic capacity of GC cells (Bilan et al., 2024). Meanwhile, in depression, levated miR-21-5p expression can downregulate CASKIN1 expression in the excitatory neurons one subpopulation (Excitatory.neurons_1 cells), thereby leading to reduced neural connectivity and abnormal synaptic plasticity (Chen J. et al., 2025). As a well-recognized tumor suppressor, miR-145-5p drives the differentiation of GC cells (Zhou et al., 2019). In the context of GC, miR-145-5p targets SMAD5 to suppress tumor cell proliferation (Wang et al., 2025). Notably, miR-145-5p was detectable in hippocampal (HIP) samples from patients with depression, indicating that this miRNA is also expressed in the central nervous system and may potentially be involved in the pathological processes of psychiatric disorders such as depression (Søkilde et al., 2025). miR-16-5p can target cyclins like CCND2 to promote GC proliferation and migration. In rat models of depression, extracellular vesicles derived from neural stem cells (NSC-EVs) have been shown to alleviate neuronal damage through the miR-16-5p/MYB axis, exerting significant neuroprotective effects (Zhuo et al., 2021). For miR-34a-5p, it targets and inhibits SIRT1 expression in GC, thereby reversing chemotherapy resistance in GC cells (Min et al., 2024). Consistent with previous studies, Chauhan et al. (Chauhan and Jain, 2025) also confirmed that miR-34a-5p, as an upstream regulator, is involved in the development of major depressive disorder (MDD). In GC, miR-491-5p functions as a tumor suppressor that inhibits tumor metastasis (Kang et al., 2021). Although no direct studies have investigated the role of miR-491-5p in depression, recent findings by Tang et al. (2022) in a mouse controlled cortical impact model revealed that mild downregulation of miR-491-5p in the brain significantly alleviates oxidative stress by targeting metallothionein 2 (MT2), activates the HIF-1α/VEGF signaling pathway, promotes cerebral microvascular angiogenesis and local cerebral blood flow recovery, and reduces neuronal apoptosis, thereby improving neurological function. It is noteworthy that the HIF-1α/VEGF pathway plays a crucial role in antidepressant effects by regulating neurogenesis and synaptic plasticity. Despite the potential importance of these miRNAs, current understanding of their functional roles in the comorbidity of GC and depression remains limited. These regulatory relationships are primarily based on database predictions and literature inferences, and rigorous validation through dual-luciferase reporter gene assays, miRNA mimic/inhibitor transfection, and *in vivo* functional experiments is still required in future studies.

Immune cells associated with diseases substantially impact disease onset, progression, and therapeutic responsiveness. Therefore, examining the link between immune cell infiltration and gene expression patterns remains essential for elucidating disease-associated immune mechanisms and identifying innovative therapeutic approaches. These findings demonstrated distinct immune cell distribution patterns among GC patients, depression patients, and healthy controls. In GC patients, elevated proportions of activated CD4 T cells, natural killer cells, and activated dendritic cells were observed, accompanied by reduced proportions of effector memory CD4 T cells, activated B cells, CD56^bright NK cells, and neutrophils. Similarly, depression patients exhibited increased proportions of central memory CD8 T cells, central memory CD4 T cells, type 1 helper T cells, type 17 helper T cells, regulatory T cells, memory B cells, CD56 bright natural killer cells, myeloid-derived suppressor cells, macrophages, monocytes, and neutrophils, while displaying decreased proportions of effector memory CD4 T cells and type 2 helper T cells. These observations indicate an activated immune microenvironment accompanied by immune evasion mechanisms in both GC and depression. Additionally, analysis of interacting gene effects on the immune microenvironment in GC patients revealed remarkably significant correlations with immune cell populations. This finding implies that these genes may modulate immune cell infiltration and functionality in GC through direct or indirect mechanisms. For example, SERPINE1 affects tumor progression and metastasis by modulating immune cell function and infiltration in GC patients (Feng et al., 2023). Bioinformatics studies have shown that COL4A1 is associated with immune cell infiltration and affects patient prognosis in GC (Xie et al., 2023). In diffuse GC, PDGFRB correlates with immune infiltration, and its inhibition can restore immune microenvironment suppression (Akiyama et al., 2023). BMP1 contributes to adverse prognosis by influencing the tumor immune microenvironment in GC patients (Zhu et al., 2022). Notably, research demonstrates that NOTCH3 associates with immune tolerance, indicating its potential function as a molecular regulator governing tumor-immune interactions in GC. Furthermore, elevated EDNRA expression within the cancer-associated fibroblast angiogenesis prognostic score system associates with immune evasion and diminished immunotherapy response in GC, emphasizing its potential as a resistance biomarker for immune checkpoint inhibitors (Xia et al., 2024). Regarding depression, investigations have demonstrated that immune dysregulation may trigger depression onset and potentially compromise antidepressant treatment efficacy (Bai et al., 2024). Nevertheless, systematic research on immunotherapeutic targets for depression remains limited, and the underlying molecular mechanisms require further elucidation.

Despite the revelation of potential genetic and molecular associations between GC and depression in this investigation, several constraints persist. For instance, dependence solely upon publicly accessible sequencing datasets may introduce inherent selection bias and batch effects, consequently restricting the generalizability of these observations. While external and experimental validations were conducted, these were concentrated predominantly on GC tissues, and the absence of sequencing datasets from patients presenting with concurrent GC and depression constituted a significant constraint of this investigation, thereby creating a substantial gap in establishing bidirectional molecular connections. Moreover, the identified pertinent pathways connecting GC and depression remain hypothetical and necessitate additional functional investigations, including knockout models or pharmacological interventions. Additionally, although correlations between hub gene expression and immune cell infiltration in patients with GC and depression were observed, the potential contributions of these immune infiltrations to disease pathogenesis remain unexplored. Furthermore, the miRNA-mRNA network was primarily built on bioinformatics predictions without experimental validation (e.g., dual-luciferase reporter assays) or in-depth pathway analysis, limiting the conclusiveness of this section. Future studies should prioritize *in vitro* experiments to verify the regulatory relationships of key miRNAs and their target genes in the comorbidity of GC and depression. Notwithstanding these constraints, these findings highlight the potential of SERPINE1, COL4A1, PDGFRB, BMP1, NOTCH3, and EDNRA as molecular connectors between GC and depression, thereby providing promising directions for subsequent research and therapeutic advancement.

## 5 Conclusion

This bioinformatics investigation revealed SERPINE1, COL4A1, PDGFRB, BMP1, NOTCH3, and EDNRA as shared hub genes linking GC and depression. The PI3K-Akt, AGE-RAGE, and proteoglycans in cancer pathways are candidate common pathways that may contribute to GC–depression comorbidity, although their non-specific nature across multiple diseases warrants cautious interpretation. Future experimental validation is still required to substantiate these conclusions.

## Data Availability

The datasets supporting the conclusions of this article are available in publicly accessible repositories. The GC-related datasets GSE113255 and GSE122401 utilized in this study can be found in the GEO database. Gene expression data for GC in TPM format were obtained from TCGA data portal.

## Glossary

GC: Gastric cancer
GEO: Gene expression omnibus database
TCGA: The cancer genome atlas
RRID: Research resource identifier
FC: Fold change
WGCNA: Weighted gene co-expression network analysis
DEGs: Differentially expressed genes
TOM: Topological overlap matrix
MEs: Module eigengenes
PPI: Protein-protein interaction
MCODE: Molecular complex detection
GO: Gene ontology
KEGG: Kyoto encyclopedia of genes and genomes
GSEA: Gene set enrichment analysis
ssGSEA: Single-sample gene set enrichment analysis
K-M: Kaplan-meier
IARC: International agency for research on cancer
WHO: World health organization
OS: Overall survival
PDB: Protein data bank
HMDD: Human microRNA disease database
ECM: Extracellular matrix
tPA: Tissue plasminogen activator
uPA: Urokinase-type plasminogen activator
PAI-1: Plasminogen activator inhibitor one
NES: Normalized enrichment score
TPM: Transcripts per million
MAD: Median absolute deviation
STRING: Search Tool for the Retrieval of Interacting Genes
MCC: Maximal clique centrality
DG: Dentate gyrus
MDD: Major depressive disorder
PNN: Perineuronal net
CADASIL: Cerebral autosomal dominant arteriopathy with subcortical infarcts and leukoencephalopathy
IRS: Immune-inflammatory response system
