# Supplementary material for: Shared molecular features and candidate pathways underlying gastric cancer–depression comorbidity: a systems biology analysis
Source: Front Bioinform. 2026 May 20;6:1836419. doi: 10.3389/fbinf.2026.1836419 (PMC13231047; doi:10.3389/fbinf.2026.1836419)
Supplement: Supplementary file 1 [file Supplementaryfile1.docx]

**Supplementary Material**

**Content**

**[1 DOWNLOAD OF GEO DATASETS (GEOquery) 1](#_Toc20665)**

**[2 DOWNLOAD OF TCGA DATASETS (BiocManager) 1](#_Toc7376)**

**[3 DIFFERENTIAL EXPRESSION ANALYSIS AND VISUALIZATION (limma) 2](#_Toc19589)**

**[4 VISUALIZATION OF DIFFERENTIAL EXPRESSION RESULTS (ggplot2) 3](#_Toc10280)**

**[5 WEIGHTED GENE CO-EXPRESSION NETWORK ANALYSIS (WGCNA) 4](#_Toc8424)**

**[6 GO AND KEGG ENRICHMENT ANALYSIS (clusterProfiler) 6](#_Toc28638)**

**[7 Visualization of GO and KEGG results (treemap) 7](#_Toc718)**

**[8 SURVIVAL ANALYSIS (survival) 7](#_Toc2100)**

**[9 GENE SET ENRICHMENT ANALYSIS (GSEA) (clusterProfiler) 8](#_Toc17491)**

**[10 SINGLE-SAMPLE GENE SET ENRCHMENT ANALYSIS (ssGSEA) (GSVA) 9](#_Toc22066)**

**[11 ANALYSIS OF THE CORRELATION BETWEEN GENES AND IMMUNE INFILTRATION 11](#_Toc3151)**

**[12 CIRCULAR HEATMAP 11](#_Toc11132)**

**[13 VENN DIAGRAM 12](#_Toc15192)**

# DOWNLOAD OF GEO DATASETS (GEOquery)

*Load the necessary R package*

> BiocManager::install("GEOquery")

> library(GEOquery)

> f = "GSExxxxxx_eSet.Rdata"

*Download and save GEO dataset*

> if(!file.exists(f)){

+ gset <- getGEO('GSExxxxxx', destdir=".",

+ AnnotGPL = F,

+ getGPL = F)

+ save(gset,file=f)

+ }

*Load pre-downloaded dataset*

> load("GSExxxxxx_eSet.Rdata")

> expr_data <- exprs(gset[[1]])

> pheno_data <- pData(gset[[1]])

> write.csv(expr_data,"GSExxxxxx_expression_matrix.csv")

> write.csv(pheno_data,"GSExxxxxx_phenotype_data.csv")

# DOWNLOAD OF TCGA DATASETS (BiocManager)

*Load the necessary R package*

> library(TCGAbiolinks)

> library(SummarizedExperiment)

> library(tidyverse)

> library(dplyr)

*Setting up the TCGA download program (TCGA-STAD as an example)*

> project <- "TCGA-STAD"

*Query and download transcriptome data from the GDC (Genomic Data Commons)*

> query <- GDCquery(project = project,

+ data.category = "Transcriptome Profiling",

+ data.type = "Gene Expression Quantification",

+ workflow.type = "STAR - Counts",

+ experimental.strategy = "RNA-Seq",

+ access = "open")

> GDCdownload(query, files.per.chunk = 100)

> GDCprepare(query, save = T, save.filename = "TCGA_STAD.Rdata")

*Load the saved RData file and extract expression data*

> load(file = "TCGA_STAD.Rdata")

> rowdata <- rowData(data)

> coldata <- colData(data)

> mRNA_data <- data[rowdata$gene_type == "protein_coding",]

> exp_TPM <- assay(mRNA_data,"tpm_unstrand")

> mRNA_symbol <- rowData(mRNA_data)$gene_name

> exprSet <- cbind(as.data.frame(mRNA_symbol), as.data.frame(exp_TPM))

*Process the expression data*

> mRNA_expr <- exprSet %>%

+ group_by(mRNA_symbol) %>%

+ summarise(across(everything(), mean)) %>%

+ filter(!is.na(mRNA_symbol) & mRNA_symbol != "") %>%

+ distinct(mRNA_symbol, .keep_all = T) %>%

+ column_to_rownames(var = "mRNA_symbol")

*Log2 transformations*

> mRNA_expr_re <- log2(mRNA_expr+1)

*Save expression matrix to file*

> write.csv(mRNA_expr_re, file = "STAD_TPM_exp_data.csv")

# DIFFERENTIAL EXPRESSION ANALYSIS AND VISUALIZATION (limma)

*Load necessary R packages*

> library(limma)

*Read the expression matrix*

> expr_data <- read.csv("expression_matrix.csv", row.names = 1)

*Visualize data before and after normalization*

> par(mfrow = c(1, 2))

> boxplot(expr_data, main = "Pre-normalization", las = 2)

> normalized_data <- normalizeBetweenArrays(expr_data)

> boxplot(normalized_data, main = "Post-normalization", las = 2)

*Read the group file*

> group_data <- read.csv("group_file.csv")

> group <- factor(group_data$Group, levels = c("Normal","Tumor"))

*Create a design matrix for the linear model*

> design <- model.matrix(~0+group)

> colnames(design) <- c("Normal","Tumor")

*Fit the linear model to the expression data*

> fit1 <- lmFit(normalized_data,design)

*Define the contrast of interest (Tumor vs Normal)*

> cont.matrix <- makeContrasts(Tumor-Normal,levels = design)

*Compute contrast results*

> fit2 <- contrasts.fit(fit1,cont.matrix)

> fit2 <- eBayes(fit2)

*Extract and save the results*

> deg_result <- topTable(fit2,coef = 1,number = dim(expdt)[1],adjust.method = "BH",

+ sort.by = "B",resort.by = "M")

> deg_result <- na.omit(deg_result )

> write.csv(deg_result,"Differential_Expression_Results.csv")

# VISUALIZATION OF DIFFERENTIAL EXPRESSION RESULTS (ggplot2)

*Load necessary R packages*

> library(ggplot2)

> library(dplyr)

> library(ggrepel)

> library(tidyverse)

*Read the DEG results*

> deg_result <- read.csv("deg_result.csv",row.names = 1)

*Define filtering criteria*

> foldChange = 1

> pval = 0.05

*Prepare data for visualization*

> data <- final %>%

+ mutate(change = as.factor(ifelse(adj.P.Val < 0.05 & abs(logFC) > 1,ifelse(logFC > 1 ,'Up','Down'),'None'))) %>%

+ rownames_to_column('gene')

*Define colors for the plot*

> mycol <- c("#55aeda","grey","#fa6464")

*Create the volcano plot*

> ggplot(data, aes(logFC, -log10(adj.P.Val)))+

+ geom_point(aes(col=change), alpha = 1, shape = 16, size = 2)+

+ scale_color_manual(values = mycol2,

+ labels = c('Down 1195', 'None 17602', "Up 1053"))+

+ scale_x_continuous(breaks = c(seq(-10, 10, 2)), limits = c(-10, 14)) +

+ labs(x="log2(FoldChange)",y="-log10(P-value)",colour = "Expression Change")+

+ geom_vline(xintercept=c(-1,1), colour="black", linetype="dashed")+

+ geom_hline(yintercept = -log10(0.05),colour="black", linetype="dashed")+

+ guides(color = guide_legend(override.aes = list(size = 5))) +

+ theme_bw() +

+ theme(panel.border = element_rect(colour = "black", fill = NA, linewidth = 0.5),

+ panel.grid.minor = element_blank(),

+ panel.background = element_blank(),

+ plot.background = element_blank(),

+ axis.title = element_text(face = "bold", color = "black", size = 10),

+ axis.text = element_text(color = "black", size = 9, face = "bold"),

+ legend.background = element_blank(),

+ legend.title = element_text(face = "bold", color = "black", size = 10),

+ legend.text = element_text(face = "bold", color = "black", size = 9),

+ legend.spacing.x = unit(0, "cm"),

+ legend.position = c(0.86, 0.88))

# WEIGHTED GENE CO-EXPRESSION NETWORK ANALYSIS (WGCNA)

*Load necessary R packages*

> library(WGCNA)

> options(stringsAsFactors = FALSE)

*Read gene expression data (genes as rows, samples as columns)*

> expr <- read.csv("expression_data.csv", row.names = 1)

> Select top 5000 genes with highest median absolute deviation

> data.mat <- t(expr[order(apply(expr, 1, mad), decreasing = TRUE)[1:5000], ])

> datExpr <- as.data.frame(data.mat)

*Calculate sample-wise Pearson correlation matrix*

> correlation_matrix <- cor(t(datExpr), method = "pearson")

> average_correlations <- apply(correlation_matrix, 1, mean)

> threshold <- mean(average_correlations) - 3 * sd(average_correlations)

> outliers <- which(average_correlations < threshold)

*Visualize outliers: plot average correlations per sample*

> plot(average_correlations, pch = 17, main = "Average Correlations with Outliers",

+ xlab = "Sample Index", ylab = "Average Correlation")

> abline(h = threshold, col = "red", lwd = 2)

> points(outliers, average_correlations[outliers],

+ pch = 4, col = "blue", cex = 1.5)

*Remove identified outliers from dataset*

> datExpr1 <- datExpr[-outliers, ]

*Sample clustering tree (before excluding outlier samples)*

> sampleTree <- hclust(dist(datExpr), method = "average")

> plot(sampleTree, main = "Sample clustering to detect outliers", sub = "", xlab = "",

+ cex.lab = 1, cex.axis = 1, cex.main = 1.2, cex = 0.5)

*Sample clustering tree (after excluding outlier samples)*

> sampleTree1 <- hclust(dist(datExpr1), method = "average")

> plot(sampleTree1, main = "Sample clustering to detect outliers", sub = "", xlab = "",

+ cex.lab = 1, cex.axis = 1, cex.main = 1.2, cex = 0.5)

*Soft threshold selection*

> powers <- c(seq(1, 10, by = 1), seq(12, 20, by = 2))

> sft <- pickSoftThreshold(datExpr1, powerVector = powers, verbose = 5)

> par(mfrow = c(1, 2))

> plot(sft$fitIndices[, 1], -sign(sft$fitIndices[, 3]) * sft$fitIndices[, 2],

+ xlab = "Soft Threshold (power)", ylab = "Scale Free Topology Model Fit, signed R^2",

+ type = "n", main = "Scale independence")

> text(sft$fitIndices[, 1], -sign(sft$fitIndices[, 3]) * sft$fitIndices[, 2],

labels = powers, cex = 0.877, col = "red")

> abline(h = 0.877, col = "red")

> plot(sft$fitIndices[, 1], sft$fitIndices[, 5],

+ xlab = "Soft Threshold (power)", ylab = "Mean Connectivity", type = "n",

+ main = "Mean connectivity")

> text(sft$fitIndices[, 1], sft$fitIndices[, 5], labels = powers, cex = 0.877, col = "red")

*Store selected power value*

> power <- sft$powerEstimate

*Network construction*

> datExpr1[] <- lapply(datExpr1, as.numeric)

> net <- blockwiseModules(datExpr1, power = power, minModuleSize = 30,

+ reassignThreshold = 0, mergeCutHeight = 0.25,

+ numericLabels = TRUE, pamRespectsDendro = FALSE,

+ saveTOMs = TRUE, corType = "pearson",

+ saveTOMFileBase = "network-TOM", verbose = 3)

*Module visualization*

> mergedColors <- labels2colors(net$colors)

> plotDendroAndColors(net$dendrograms[[1]], mergedColors[net$blockGenes[[1]]],

+ "Module colors", dendroLabels = FALSE, hang = 0.03,

+ addGuide = TRUE, guideHang = 0.05, cex.colorLabels = 1,

+ cex.dendroLabels = 1, cex.rowText = 1)

*Module-trait association*

> MEs <- moduleEigengenes(datExpr1, mergedColors)$eigengenes

> MEs <- orderMEs(MEs)

*Read clinical trait data*

> clinical <- read.csv("your_clinical_data.csv", header = TRUE, row.names = 1)

> datTraits <- clinical

*Calculate module-trait correlations*

> moduleTraitCor <- cor(MEs, datTraits, use = "p")

> moduleTraitValue <- corPvalueStudent(moduleTraitCor, nrow(datExpr1))

*Module-trait visualization*

> textMatrix <- paste(signif(moduleTraitCor, 2), "\n(", signif(moduleTraitValue, 1), ")", sep = "")

> dim(textMatrix) <- dim(moduleTraitCor)

> par(mar = c(2, 5, 1, 2))

> labeledHeatmap(Matrix = moduleTraitCor, xLabels = colnames(datTraits),

+ yLabels = names(MEs), ySymbols = names(MEs), colorLabels = TRUE,

+ colors = blueWhiteRed(50), textMatrix = textMatrix, setStdMargins = FALSE,

+ cex.text = 0.6, cex.lab = 0.6, zlim = c(-1, 1), xLabelsAngle = TRUE,

+ main = "Module-trait relationships")

*Get the genes in the module (blue)*

> moduleColors <- labels2colors(net$colors)

> gene_list <- names(datExpr)[moduleColors == "blue"]

> write.csv(gene_list, "blue_module_genes.csv")

# GO AND KEGG ENRICHMENT ANALYSIS (clusterProfiler)

*Load necessary R packages*

> required_packages <- c("clusterProfiler", "org.Hs.eg.db", "ggplot2", "readxl")

> BiocManager::install(required_packages, ask = FALSE)

> library(clusterProfiler)

> library(org.Hs.eg.db)

> library(ggplot2)

> library(readxl)

*Read gene list*

> data <- read_excel("your_gene_list.xlsx")

> genenames <- data$Gene

*Perform GO enrichment analysis*

> ego <- enrichGO(

+ gene = gene_list,

+ OrgDb = org.Hs.eg.db,

+ ont = "ALL",

+ pAdjustMethod = "BH",

+ pvalueCutoff = 0.05,

+ qvalueCutoff = 0.2,

+ keyType = "SYMBOL"

+ )

> write.csv(ego, "GO_BP_MF_CC_enrichment_results.csv")

*Convert gene symbols to ENTREZ IDs*

> gene_conversion <- bitr(gene_list,

+ fromType = "SYMBOL",

+ toType = "ENTREZID",

+ OrgDb = org.Hs.eg.db)

*Perform KEGG pathway analysis*

> kegg_result <- enrichKEGG(

+ gene = gene_conversion$ENTREZID,

+ organism = 'hsa',

+ keyType = 'kegg',

+ pAdjustMethod = 'fdr',

+ pvalueCutoff = 0.05,

+ qvalueCutoff = 0.2

+ )

> write.csv(kegg_result, "KEGG_enrichment_results.csv")

# Visualization of GO and KEGG results (treemap)

*Load necessary R packages*

> library(treemap)

*Read the results of the functional enrichment analysis (GO-BP)*

> bp_re <- read.csv("GC_BP_TOP20.csv",row.names = 1)

*Plotting*

> treemap(bp_re,

+ index="Description",

+ vSize="Count",

+ vColor="p.adjust",

+ fontsize.labels=c(14,12),

+ fontcolor.labels = "white",

+ align.labels=list(c("left", "center")),

+ border.col="black",

+ border.lwds=c(1,1),

+ title="Treemap of GO terms",

+ fontsize.title=16,

+ alpha=0.5

+ )

*Read the results of the functional enrichment analysis (KEGG)*

> kegg_re <- read.csv("GC_BP_TOP20.csv",row.names = 1)

*Plotting*

> treemap(kegg_re,

+ index="Description",

+ vSize="Count",

+ vColor="p.adjust",

+ fontsize.labels=c(14,12),

+ fontcolor.labels = "white",

+ align.labels=list(c("left", "center")),

+ border.col="black",

+ border.lwds=c(1,1),

+ title="Treemap of GO terms",

+ fontsize.title=16,

+ alpha=0.5

+ )

# SURVIVAL ANALYSIS (survival)

*Load necessary R packages*

> library(survival)

> library(survminer)

> library(readxl)

> library(rms)

*Read survival data*

> surv_data <- read_xlsx("survival_data_TPM.xlsx")

*Prepare data for analysis*

> dd <- datadist(surv_data)

> options(datadist = dd)

> attach(surv_data)

*Binarize gene expression based on median values*

> genes <- c("GENE1", "GENE2", "GENE3", "GENE4", "GENE5", "GENE6")

> for (gene in genes) {

+ surv_data[[gene]] <- ifelse(surv_data[[gene]] > median(surv_data[[gene]]), "High", "Low")

+ }

*Define survival object*

> surv_obj <- Surv(futime, fustat == 1)

*Fit survival model (GENE1)*

> fit <- survfit(surv_obj ~ GENE1, data = surv_data)

*Visualize survival curves*

> ggsurvplot(

+ fit1,

+ pval = TRUE,

+ conf.int = TRUE,

+ palette = c("#55aeda", "#fa6464"),

+ conf.int.style = "step",

+ legend.labs = c("Low GENE1", "High GENE1"),

+ legend.title = "",

+ legend = c(0.8, 0.95)

+ )

# GENE SET ENRICHMENT ANALYSIS (GSEA) (clusterProfiler)

*Load necessary R packages*

> library(clusterProfiler)

> library(ggplot2)

*Read gene expression data (excluding normal samples)*

> expr <- read.csv("expression_data_excluded_normal.csv", row.names = 1)

> t_expr <- as.data.frame(t(expr))

*Sort samples by GENE1 expression*

> t_expr_sorted <- t_expr[order(t_expr$GENE1,decreasing = F),]

> expr_sorted <- as.data.frame(t(t_expr_sorted))

*Divided into high and low expression group*

> group_list=c(rep("low",ncol(GENE1)/2),rep("high",ncol(GENE1)/2))

> colData <- data.frame(row.names = colnames(GENE1),

+ Group = group_list)

*Set up linear model with limma*

group <- factor(colData$Group, levels = c("high","low"))

design <- model.matrix(~0 + newgroup)

colnames(design) <- c("high","low")

*Fit the linear model to the expression data*

> fit1 <- lmFit(normalized_data,design)

Define the contrast of interest (Tumor vs Normal)

> cont.matrix <- makeContrasts(Tumor-Normal,levels = design)

*Compute contrast results*

> fit2 <- contrasts.fit(fit1,cont.matrix)

> fit2 <- eBayes(fit2)

*Extract the results*

> deg_result <- topTable(fit2,coef = 1,number = dim(expdt)[1],adjust.method = "BH",

+ sort.by = "B",resort.by = "M")

> deg_result <- na.omit(deg_result )

*Filter significant genes*

> out <- deg_result[deg_result$adj.P.Val < 0.05,]

*Sorting genes by logFC values*

> geneList <- out$logFC

> names(geneList) <- rownames(out)

> geneList_sorted <- sort(geneList, decreasing = TRUE)

*Load pathway gene sets*

> kegmt <- read.gmt("h.all.v2023.2.Hs.symbols.gmt")

*Perform GSEA analysis*

> KEGG <- GSEA(geneList2, TERM2GENE = kegmt)

*Visualization results (top 5)*

> num <- 5

> gseaplot2(KEGG, geneSetID = rownames(KEGG@result)[tail(order(KEGG@result$enrichmentScore),num)])

# SINGLE-SAMPLE GENE SET ENRCHMENT ANALYSIS (ssGSEA) (GSVA)

*Load necessary R packages*

> library(GSVA)

> library(tibble)

> library(reshape2)

> library(ggplot2)

*Read the gene expression matrix*

> exp_tpm <- read.csv("expression_data.csv", row.names = 1)

> exp_tpm <- as.matrix(exp_tpm)

*Read the immune gene set*

> cellMarker <- read.csv("immune_gene_set.csv", header = F)

> cellMarker <- cellMarker %>% column_to_rownames("V1") %>% t()

> a <- cellMarker

> a <- a[1:nrow(a), ]

> set <- colnames(a)

> geneSet <- list()

> i <- "Activated CD8 T cell"

> for (i in set) {

+ x <- as.character(a[,i])

+ x <- x[nchar(x)!=0]

+ x <- as.character(x)

+ geneSet[[i]] <-x

+ }

*Setup parameters*

> params <- gsvaParam(

+ exprData = as.matrix(Exp_tpm),

+ geneSets = geneSet,

+ minSize = 1,

+ maxSize = Inf,

+ kcdf = "Gaussian",

+ tau = 1,

+ maxDiff = TRUE,

+ absRanking = FALSE

+ )

*GSVA analysis*

> gsva_result <- gsva(params, verbose = TRUE,

+ BPPARAM = BiocParallel::SerialParam(progressbar = TRUE))

*Read sample grouping files (tumor and normal)*

> group_dt <- read.csv("group_file".csv)

*Add grouping information to results*

> gsva_result <- gsva_result %>% t() %>% as.data.frame()

> gsva_result$Group <- group_dt$Group

*Create ggplot2 input matrix*

> gsva_result <- rownames_to_column(gsva_result, var = "GeneName")

> plot_data <- melt(gsva_result,id.vars=c("GeneName","Group"))

> colnames(plot_data) <- c("Sample","Group","Celltype","Score")

*Visualization results*

> ggplot(plot_data,aes(Celltype,Score)) +

+ geom_boxplot(aes(fill = Group),outlier.shape = 20)+

+ theme_bw() +

+ labs(x = NULL, y = "Score") +

+ scale_fill_manual(values = c("#55aeda", "#fa6464"))+

+ stat_compare_means(aes(group = Group,label = after_stat(p.signif)),

+ method = "wilcox.test",

+ hide.ns = T)+

+ theme(plot.margin=unit(c(1,1,1,1),'cm'),

+ plot.title = element_text(size = 12,color="black",hjust = 0.5),

+ axis.title = element_text(size = 12,color ="black"),

+ axis.text = element_text(size= 12,color = "black"),

+ panel.grid.minor.y = element_blank(),

+ panel.grid.minor.x = element_blank(),

+ axis.text.x = element_text(angle = 45, hjust = 1 ),

+ panel.grid=element_blank(),

+ legend.position = "top",

+ legend.text = element_text(size= 12),

+ legend.title= element_text(size= 12))

# ANALYSIS OF THE CORRELATION BETWEEN GENES AND IMMUNE INFILTRATION

*Load necessary R packages*

> library(Hmisc)

*Read the gene expression matrix*

> exp_tpm <- read.csv("expression_data.csv", row.names = 1)

*Read the GSEA result*

> gsea_r <- read.csv("ssGSEA_Result.csv",row.names = 1)

> gsea_r_t <- t(gsea_r)

> gene_name <- c("GENE1","GENE2","GENE3","GENE4","GENE5","GENE6")

> rdata = t(rbind(gsea_r_t,exp_tpm[gene_name,]))

*Calculate correlation*

> cor = rcorr(nc)$r[1:nrow(ssgsea), (ncol(rdata)-length(gene_name) + 1):ncol(nc)]

> p_value = rcorr(nc)$P[1:nrow(ssgsea), (ncol(rdata)-length(gene_name) + 1):ncol(nc)]

*Save results*

> write.csv(m,"correlation.csv")

> write.csv(p,"correlation_p_value.csv")

# CIRCULAR HEATMAP

*Load necessary R packages*

> library(ComplexHeatmap)

> library(circlize)

*Read correlation analysis results*

> cor_dt <- read.csv("correlation.csv",row.names = 1)

*Custom color*

> range(cor_dt)

> mycol=colorRamp2(c(-0.54, 0.18, 0.54),c("blue", "white", "red"))

*Plotting*

> circos.heatmap(cir1,

+ col=mycol,

+ dend.side="inside",

+ rownames.side="outside",

+ rownames.col="black",

+ rownames.cex=0.8,

+ rownames.font=1,

+ cluster=TRUE)

> circos.clear()

# VENN DIAGRAM

*Load necessary R packages*

> library(venn)

*Read gene list*

> gene_list1 <- read.csv("dataset1.csv")

> gene_list2 <- read.csv("dataset2.csv")

> gene_list3 <- read.csv("dataset3.csv")

*Creating a list of gene collections*

> gene_list <- list(

+ A = unique(gene_list1),

+ B = unique(gene_list2),

+ C = unique(gene_list3)

+ )

*Plotting venn diagrams*

> venn(gene_list,

+ box = FALSE,

+ ellipse = FALSE,

+ plotsize = 13,

+ borders = TRUE,

+ ilcs = 2,

+ sncs = 1.8,

+ lty = 1,

+ zcolor = c("skyblue", "pink", "lightgreen"),

+ col = c("skyblue", "pink", "lightgreen")

+ )
