# Supplementary material for: Shared molecular features and candidate pathways underlying gastric cancer–depression comorbidity: a systems biology analysis
Source: Front Bioinform. 2026 May 20;6:1836419. doi: 10.3389/fbinf.2026.1836419 (PMC13231047; doi:10.3389/fbinf.2026.1836419)
Supplement: Supplementary file 2 [file Table1.docx]

Supplementary Material

**Content**

**[1 Table S1: Primer for qRT-PCR. 1](#_Toc16464)**

**[2 Table S2: 130 gastric cancer-related genes. 1](#_Toc25398)**

**[3 Table S3: 534 depression-related genes. 2](#_Toc8354)**

**[4 Table S4: The top 20 biological processes of gastric cancer-related genes. 3](#_Toc16761)**

**[5 Table S5: The top 20 KEGG signaling pathways of gastric cancer-related genes. 6](#_Toc12626)**

**[6 Table S6: The topological characteristic of 21 nodes in the PPI network of gastric cancer-related genes. 7](#_Toc4511)**

**[7 Table S7: The KEGG signaling pathways of cluster I genes in the PPI network of gastric cancer-related genes. 9](#_Toc11356)**

**[8 Table S8: The clustering of PPI networks of gastric cancer-related genes. 10](#_Toc1960)**

**[9 Table S9: The top 20 biological processes of depression-related genes. 11](#_Toc6126)**

**[10 Table S10: The top 20 KEGG signaling pathways of depression-related genes. 20](#_Toc21130)**

**[11 Table S11: The topological characteristic of 126 nodes in the PPI network of depression-related genes. 25](#_Toc10911)**

**[12 Table S12: The clustering of PPI networks of depression-related genes. 31](#_Toc27668)**

**[13 Table S13: The KEGG signaling pathways of cluster I genes in the PPI network of depression-related genes. 33](#_Toc26186)**

**[14 Table S14: Common miRNAs between gastric cancer and depression. 35](#_Toc10452)**

# Table S1: Primer for qRT-PCR.

| **Gene** | **Sequences** |
| --- | --- |
| β-actin | For：5′- 3′AACCGCGAGAAGATGACCCAG  Rev：5′- 3′GGATAGCACAGCCTGGATAGCAA |
| BMP1 | For：5′- 3′GGGTCATCCCCTTTGTCATTG  Rev：5′- 3′GCAAGGTCGATAGGTGAACACA |
| COL4A1 | For：5′- 3′GGACTACCTGGAACAAAAGGG  Rev：5′- 3′GCCAAGTATCTCACCTGGATCA |
| EDNRA | For：5′- 3′TCGGGTTCTATTTCTGTATGCCC  Rev：5′- 3′TGTTTTTGCCACTTCTCGACG |
| NOTCH3 | For：5′- 3′TGGCGACCTCACTTACGACT  Rev：5′- 3′CACTGGCAGTTATAGGTGTTGAC |
| PDGFRB | For：5′- 3′AGCACCTTCGTTCTGACCTG  Rev：5′- 3′TATTCTCCCGTGTCTAGCCCA |
| SERPINE1 | For：5′- 3′ACCGCAACGTGGTTTTCTCA  Rev：5′- 3′TTGAATCCCATAGCTGCTTGAAT |

# Table S2: 130 gastric cancer-related genes.

| COL11A1, CST1, COL10A1, WNT2, INHBA, SFRP4, FAP, COL8A1, THBS2, STRA6, FNDC1, EBF2, NOX4, SULF1, P4HA3, BGN, CTHRC1, SPOCK1, ITGBL1, COMP, ADAMTS12, PRND, CPZ, OSR2, PRRX1, SERPINE1, FOXS1, CST2, COL1A1, THY1, MFAP2, ADAM12, ASPN, OLFML2B, COL6A3, COL3A1, ISM1, COL1A2, ADAMTS2, CPXM1, MMP11, RPLP0P2, FMO1, CRABP2, SPARC, FN1, KCNQ3, TIMP1, DOK5, COL12A1, COL4A1, LUM, HEYL, CORIN, LZTS1, CACNA1E, NID2, IGFBP7, NNMT, BICC1, VCAN, NTM, GEM, PDGFRB, CSMD2, PGF, HTRA3, CDH11, SYNDIG1, THBS1, CTSK, GFPT2, ANTXR1, MMP16, LRRC32, RCN3, FSTL1, TNFSF4, ZNF469, CPT1C, ISLR, BMP8A, COL5A2, SERPINE2, PMEPA1, BMP1, PLXDC2, PDE3A, SCARF2, COL4A2, GUCY1A2, SPON2, RAB31, FBN1, FKBP10, SERPINH1, CLEC11A, C3, SEMA6B, KIF26B, PDPN, VGLL3, CD248, COL5A1, ITGA11, ADAMTS6, TGFBI, ADAMTS4, EGR2, NOTCH3, EDNRA, CDH13, GPR176, OSMR, CD109, AEBP1, ADAMTS9, CHN1, SULF2, MSC, GAL3ST4, MRC2, SH3PXD2B, GLI2, TGFB2, CCND2, KIAA1755, FSTL3, TGM2, SHROOM4 |
| --- |

# Table S3: 534 depression-related genes.

| SLC6A4, HTR2A, TPH2, BDNF, FKBP5, COMT, GRIN1, GRIN2A, MECP2, DRD2, APOE, DRD4, MAOA, SLC6A3, GRIN2B, TNF, CACNA1C, HTR1A, IL6, NOTCH3, TWNK, GRIA1, NR3C1, GAD1, MTHFR, TH, CRH, PRL, TPH1, WFS1, MAPT, DISC1, SLC6A2, PTEN, DLG4, DRD3, HTR2C, CRP, NRXN1, IL1B, NTRK2, CTNNB1, NR4A2, POMC, POLG, DEAF1, IL10, ACE, RELN, CYP2D6, GLI3, INS, PRKN, LEP, NOTCH1, TCF4, NR3C2, CRHR1, OXT, HTR1B, CREB1, GNB3, ALB, FGFR3, ESR1, IFNG, NR5A1, NPY, ALG8, FLNA, TP53, KRAS, DRD5, FMR1, SNCA, MTOR, DAOA, RORA, APP, FGFR1, AKT1, IGF1, MAOB, C9orf72, GDNF, OXTR, OPRM1, DRD1, XBP1, GRIA2, HTR3A, SNAP25, PRNP, JAK2, NGF, GRM7, GRIK2, TRAPPC9, GRIA3, MAP2K1, XK, NOS1, TOR1A, TUSC3, HCRT, GRM5, DBH, PER3, CLOCK, CHD7, PAH, SOD1, FGFR2, CNR1, GHRL, PCLO, CD36, CRY1, MED12, ADCY5, CYP2C19, PER2, HTT, GSK3B, PDGFRB, EHMT1, SLC18A2, AR, SYN1, PLA2G6, GNB1, SETD2, SCN8A, ATP1A3, ADIPOQ, LRRK2, CCK, PSEN1, GFAP, GNAS, TSPO, CBS, ABCB1, SLC2A1, CHRNA4, PDYN, PGAP1, DCTN1, NPC1, ATP6AP2, GCH1, KDM6B, BRCA2, PAX6, DTNBP1, MYT1L, GAD2, P2RX7, CHRM2, ITGB3, SST, ATXN2, NOS3, NRG1, SERPINA1, PANK2, CAMK2B, APOB, FOS, KDM5B, GRM1, SYP, ASXL1, GABRB3, HLA-DRB1, SLC17A5, TACR1, TARDBP, CYP3A4, PON1, ATRX, PVALB, RPS6KA3, GABRA1, CTLA4, HLA-DQB1, CAMK2A, VCP, RYR1, NEFL, GAL, PLAU, HTR1D, CC2D1A, DNMT1, ATM, CCL2, NSUN2, TAC1, ATXN3, TTN, PIK3CA, SLC6A1, ATP2A2, TACR3, ANKK1, TBP, KIT, GRIA4, HBB, NR1D1, NRAS, CP, MAPK1, PDE4B, CXCL8, IL2, SLC1A1, TIMELESS, MSH6, USH2A, ADCYAP1, ATP7B, VEGFA, TGFBR2, SIRT1, ALPL, STAT3, AVPR1B, TGFB1, PINK1, SYNE1, SLC1A2, S100B, FUS, GABRG2, ACSL4, IDO1, ARSA, GRN, CRY2, NPAS2, AHI1, PCDH15, SERPINA3, SGCE, COL4A1, PIK3R1, TTR, DAO, MEN1, ALDH2, SLC1A3, SMPD1, REN, INSR, FGD1, LMAN2L, PRRT2, HTR6, CASR, ST3GAL3, PPARG, HMBS, NTF3, CRHR2, CAMK2G, CSF1R, TRH, FHIT, CYP27A1, ERBB4, RAC1, NLGN3, PSAP, ELN, AGT, COL9A3, GNRH1, SERPINE1, RRM2B, CYP2B6, HLA-B, MOCOS, C19orf12, TLR4, CRHBP, GP1BB, MTR, OTX2, VDR, AGTR1, GH1, IGF2, ADRA2A, ADORA2A, SQSTM1, GPR50, NLRP3, MEFV, CYP19A1, CDH23, GALC, PDE11A, GLA, TBX1, CLN6, HP, PARK7, CACNA1H, NEK1, IL17A, LGI1, MAD1L1, CHAT, SIGMAR1, ASMT, HMOX1, GRM2, ATP13A2, PPARGC1A, WARS2, REST, GRM3, FAS, AVP, APOA1, MOG, ACHE, SMS, PTH, VGF, COQ2, PPP1R1B, TNFRSF1A, SLC25A4, SLC45A1, SPAST, IL1RN, ABCC8, AFG3L2, ACTB, IL4, CAT, NDE1, FIG4, GPT, FGF8, HCRTR1, PHF8, MMP9, AGO1, COL9A2, HLA-DQA1, PCNT, F3, WNT1, BRCA1, GJA1, EDN1, TERT, ATXN7, ESR2, AVPR1A, HOMER1, ADH1C, SCN10A, FAAH, ALDH18A1, IL2RA, FBXW7, CALB2, BCL2, IFNA1, EPO, IDUA, COL9A1, NCAM1, TSNAX, CALCA, CHMP2B, KCNT1, CRTAP, EGFR, FZD6, BCS1L, TREM2, ABL1, GABRA3, MYO7A, TBX5, PPT1, SELP, LINS1, ADH1B, PDE4A, IMPA2, PTPN22, FTL, ADRB2, AANAT, PAFAH1B1, ABCA1, DPP4, IGF1R, NTRK1, PTGS2, VPS13C, ATN1, SLC29A3, MPO, THOC2, SLC6A8, NOS2, IL18, GRIK4, TMEM106B, SOD2, JMJD1C, IL1A, HCN1, ICAM1, PRKCG, EDNRA, PYY, FA2H, CRBN, POLG2, BCHE, FGF2, OPN4, GAP43, CLCN4, PPIG, SOX2, RSRC1, CALB1, STAT1, BMP1, NDUFS4, SHBG, DHDDS, FTO, DHH, NOS1AP, TCF7L2, TET2, ALMS1, PREP, OPTN, EGF, PPP2CA, PLG, ENG, NTRK3, TFAP2B, LTA, MKS1, TRPV1, WDR26, STAR, PLA2G4A, PPARA, AMH, PRKAR1A, SLC17A7, FGF13, MIF, MMP2, ABCC9, PHIP, MLH1, BMP6, IRF6, CDKN2A, MAPK3, WDR11, SNCAIP, PPOX, ABCA13, CNTF, PROKR2, FGF14, UNC13A, GNRHR, AQP4, CYP2C9, DGUOK, NGFR, AMY1B, GRIN2D, CSF3, CCL5, PDGFB, CALR, UCHL1, ASPM, HTR3B, DNAJC13, TBK1, SLC5A7, CFH, EYA1, MSH2, ARVCF, LEPR, HTR7, SOCS1, ADAM10, NRGN, CLU, IL13, TG, GSTM1, LIFR, ANOS1, BCR, MAG, TSC22D3, HNMT, CHRNB2, MTRR, DNAH8, PPP2R2B, TNFAIP3, KISS1, SRPX2, SLC12A2, MCPH1, FANCD2, ATXN10, IL2RB, TDO2, CCND1 |
| --- |

# Table S4: The top 20 biological processes of gastric cancer-related genes.

| **ID** | **Description** | **GeneRatio** | **BgRatio** | **pvalue** | **p.adjust** | **qvalue** | **geneID** | **Count** |
| --- | --- | --- | --- | --- | --- | --- | --- | --- |
| GO:0030198 | extracellular matrix organization | 36/126 | 321/18870 | 3.07E-34 | 2.76E-31 | 2.22E-31 | COL4A1/BMP1/COL11A1/COL10A1/FAP/COL8A1/SULF1/COMP/ADAMTS12/COL1A1/OLFML2B/COL3A1/COL1A2/ADAMTS2/MMP11/COL12A1/LUM/NID2/CTSK/ANTXR1/MMP16/ZNF469/COL5A2/COL4A2/FKBP10/SERPINH1/PDPN/COL5A1/ADAMTS6/TGFBI/ADAMTS4/AEBP1/ADAMTS9/SULF2/SH3PXD2B/TGFB2 | 36 |
| GO:0043062 | extracellular structure organization | 36/126 | 322/18870 | 3.44E-34 | 2.76E-31 | 2.22E-31 | COL4A1/BMP1/COL11A1/COL10A1/FAP/COL8A1/SULF1/COMP/ADAMTS12/COL1A1/OLFML2B/COL3A1/COL1A2/ADAMTS2/MMP11/COL12A1/LUM/NID2/CTSK/ANTXR1/MMP16/ZNF469/COL5A2/COL4A2/FKBP10/SERPINH1/PDPN/COL5A1/ADAMTS6/TGFBI/ADAMTS4/AEBP1/ADAMTS9/SULF2/SH3PXD2B/TGFB2 | 36 |
| GO:0045229 | external encapsulating structure organization | 36/126 | 323/18870 | 3.85E-34 | 2.76E-31 | 2.22E-31 | COL4A1/BMP1/COL11A1/COL10A1/FAP/COL8A1/SULF1/COMP/ADAMTS12/COL1A1/OLFML2B/COL3A1/COL1A2/ADAMTS2/MMP11/COL12A1/LUM/NID2/CTSK/ANTXR1/MMP16/ZNF469/COL5A2/COL4A2/FKBP10/SERPINH1/PDPN/COL5A1/ADAMTS6/TGFBI/ADAMTS4/AEBP1/ADAMTS9/SULF2/SH3PXD2B/TGFB2 | 36 |
| GO:0030199 | collagen fibril organization | 17/126 | 65/18870 | 4.87E-23 | 2.62E-20 | 2.10E-20 | BMP1/COL11A1/COMP/ADAMTS12/COL1A1/COL3A1/COL1A2/ADAMTS2/MMP11/COL12A1/LUM/COL5A2/FKBP10/SERPINH1/COL5A1/AEBP1/TGFB2 | 17 |
| GO:0061448 | connective tissue development | 20/126 | 285/18870 | 3.96E-15 | 1.70E-12 | 1.37E-12 | PDGFRB/BMP1/COL11A1/EBF2/SULF1/BGN/COMP/ADAMTS12/OSR2/PRRX1/COL1A1/COL3A1/TIMP1/CTSK/BMP8A/SERPINH1/COL5A1/TGFBI/SULF2/SH3PXD2B | 20 |
| GO:0051216 | cartilage development | 16/126 | 207/18870 | 6.07E-13 | 2.17E-10 | 1.75E-10 | BMP1/COL11A1/SULF1/BGN/COMP/ADAMTS12/OSR2/PRRX1/COL1A1/COL3A1/TIMP1/CTSK/BMP8A/SERPINH1/TGFBI/SULF2 | 16 |
| GO:0001503 | ossification | 21/126 | 444/18870 | 1.76E-12 | 5.40E-10 | 4.34E-10 | BMP1/COL11A1/CTHRC1/COMP/ADAMTS12/OSR2/COL1A1/ASPN/COL1A2/VCAN/CDH11/CTSK/MMP16/BMP8A/COL5A2/CLEC11A/ITGA11/EGR2/MRC2/GLI2/FSTL3 | 21 |
| GO:0032963 | collagen metabolic process | 12/126 | 106/18870 | 5.97E-12 | 1.60E-09 | 1.29E-09 | INHBA/FAP/COL1A1/COL1A2/ADAMTS2/MMP11/CTSK/MMP16/RCN3/SERPINH1/COL5A1/MRC2 | 12 |
| GO:0060840 | artery development | 12/126 | 111/18870 | 1.04E-11 | 2.48E-09 | 2.00E-09 | PDGFRB/NOTCH3/EDNRA/STRA6/COMP/PRRX1/COL3A1/FKBP10/ADAMTS6/EGR2/ADAMTS9/TGFB2 | 12 |
| GO:0048844 | artery morphogenesis | 10/126 | 80/18870 | 1.36E-10 | 2.92E-08 | 2.35E-08 | PDGFRB/NOTCH3/EDNRA/STRA6/COMP/PRRX1/COL3A1/FKBP10/ADAMTS9/TGFB2 | 10 |
| GO:0060348 | bone development | 14/126 | 215/18870 | 1.82E-10 | 3.56E-08 | 2.86E-08 | SFRP4/SULF1/BGN/COMP/ADAMTS12/OSR2/COL1A1/COL3A1/MMP16/FBN1/SERPINH1/SULF2/SH3PXD2B/TGM2 | 14 |
| GO:0031589 | cell-substrate adhesion | 17/126 | 356/18870 | 2.37E-10 | 4.24E-08 | 3.41E-08 | SERPINE1/COL8A1/SPOCK1/ITGBL1/ADAMTS12/COL1A1/THY1/COL3A1/FN1/NID2/CDH11/THBS1/ANTXR1/PDPN/ITGA11/CDH13/ADAMTS9 | 17 |
| GO:0071560 | cellular response to transforming growth factor beta stimulus | 15/126 | 287/18870 | 8.49E-10 | 1.40E-07 | 1.13E-07 | WNT2/COL1A1/ASPN/COL3A1/COL1A2/HTRA3/THBS1/CTSK/LRRC32/PMEPA1/PDE3A/COL4A2/FBN1/CD109/TGFB2 | 15 |
| GO:0071559 | response to transforming growth factor beta | 15/126 | 293/18870 | 1.13E-09 | 1.62E-07 | 1.30E-07 | WNT2/COL1A1/ASPN/COL3A1/COL1A2/HTRA3/THBS1/CTSK/LRRC32/PMEPA1/PDE3A/COL4A2/FBN1/CD109/TGFB2 | 15 |
| GO:0035987 | endodermal cell differentiation | 8/126 | 49/18870 | 1.13E-09 | 1.62E-07 | 1.30E-07 | COL11A1/INHBA/COL8A1/FN1/COL12A1/COL5A2/COL4A2/COL5A1 | 8 |
| GO:0072001 | renal system development | 15/126 | 324/18870 | 4.44E-09 | 5.78E-07 | 4.65E-07 | COL4A1/PDGFRB/NOTCH3/EDNRA/STRA6/SULF1/OSR2/HEYL/BICC1/FBN1/KIF26B/ADAMTS6/SULF2/GLI2/TGFB2 | 15 |
| GO:0001706 | endoderm formation | 8/126 | 58/18870 | 4.58E-09 | 5.78E-07 | 4.65E-07 | COL11A1/INHBA/COL8A1/FN1/COL12A1/COL5A2/COL4A2/COL5A1 | 8 |
| GO:0035904 | aorta development | 8/126 | 65/18870 | 1.16E-08 | 1.38E-06 | 1.11E-06 | PDGFRB/EDNRA/COL3A1/FKBP10/ADAMTS6/EGR2/ADAMTS9/TGFB2 | 8 |
| GO:0007178 | transmembrane receptor protein serine/threonine kinase signaling pathway | 16/126 | 410/18870 | 1.49E-08 | 1.68E-06 | 1.35E-06 | INHBA/SFRP4/SULF1/COMP/ASPN/COL3A1/COL1A2/HTRA3/THBS1/LRRC32/FSTL1/PMEPA1/FBN1/CD109/TGFB2/FSTL3 | 16 |
| GO:0001822 | kidney development | 14/126 | 314/18870 | 2.42E-08 | 2.59E-06 | 2.09E-06 | PDGFRB/NOTCH3/EDNRA/STRA6/SULF1/OSR2/HEYL/BICC1/FBN1/KIF26B/ADAMTS6/SULF2/GLI2/TGFB2 | 14 |

# Table S5: The top 20 KEGG signaling pathways of gastric cancer-related genes.

| **ID** | **Description** | **GeneRatio** | **BgRatio** | **pvalue** | **p.adjust** | **qvalue** | **geneID** | **Count** |
| --- | --- | --- | --- | --- | --- | --- | --- | --- |
| hsa04974 | Protein digestion and absorption | 12/61 | 103/8645 | 4.57E-12 | 5.94E-10 | 5.19E-10 | COL4A1/COL11A1/COL10A1/COL8A1/COL1A1/COL6A3/COL3A1/COL1A2/COL12A1/COL5A2/COL4A2/COL5A1 | 12 |
| hsa04512 | ECM-receptor interaction | 10/61 | 89/8645 | 4.68E-10 | 3.04E-08 | 2.66E-08 | COL4A1/THBS2/COMP/COL1A1/COL6A3/COL1A2/FN1/THBS1/COL4A2/ITGA11 | 10 |
| hsa04510 | Focal adhesion | 13/61 | 203/8645 | 1.10E-09 | 4.75E-08 | 4.15E-08 | COL4A1/PDGFRB/THBS2/COMP/COL1A1/COL6A3/COL1A2/FN1/PGF/THBS1/COL4A2/ITGA11/CCND2 | 13 |
| hsa05165 | Human papillomavirus infection | 15/61 | 331/8645 | 5.79E-09 | 1.88E-07 | 1.65E-07 | COL4A1/PDGFRB/NOTCH3/WNT2/THBS2/COMP/COL1A1/COL6A3/COL1A2/FN1/HEYL/THBS1/COL4A2/ITGA11/CCND2 | 15 |
| hsa04933 | AGE-RAGE signaling pathway in diabetic complications | 9/61 | 100/8645 | 2.72E-08 | 7.06E-07 | 6.17E-07 | SERPINE1/COL4A1/NOX4/COL1A1/COL3A1/COL1A2/FN1/COL4A2/TGFB2 | 9 |
| hsa04151 | PI3K-Akt signaling pathway | 14/61 | 359/8645 | 1.34E-07 | 2.91E-06 | 2.54E-06 | COL4A1/PDGFRB/THBS2/COMP/COL1A1/COL6A3/COL1A2/FN1/PGF/THBS1/COL4A2/ITGA11/OSMR/CCND2 | 14 |
| hsa05146 | Amoebiasis | 7/61 | 102/8645 | 6.70E-06 | 0.000124365 | 0.000108756 | COL4A1/COL1A1/COL3A1/COL1A2/FN1/COL4A2/TGFB2 | 7 |
| hsa04350 | TGF-beta signaling pathway | 6/61 | 108/8645 | 0.000105114 | 0.001708103 | 0.001493726 | INHBA/THBS1/LRRC32/BMP8A/FBN1/TGFB2 | 6 |
| hsa05144 | Malaria | 4/61 | 50/8645 | 0.000405417 | 0.005856024 | 0.005121058 | THBS2/COMP/THBS1/TGFB2 | 4 |
| hsa05205 | Proteoglycans in cancer | 7/61 | 205/8645 | 0.00056072 | 0.00728936 | 0.006374501 | WNT2/COL1A1/COL1A2/FN1/LUM/THBS1/TGFB2 | 7 |
| hsa04390 | Hippo signaling pathway | 6/61 | 157/8645 | 0.00079397 | 0.009383276 | 0.008205618 | SERPINE1/WNT2/BMP8A/GLI2/TGFB2/CCND2 | 6 |
| hsa04926 | Relaxin signaling pathway | 5/61 | 129/8645 | 0.00208709 | 0.022610141 | 0.019772431 | COL4A1/COL1A1/COL3A1/COL1A2/COL4A2 | 5 |
| hsa04145 | Phagosome | 5/61 | 152/8645 | 0.004240821 | 0.042408208 | 0.03708572 | THBS2/COMP/THBS1/C3/MRC2 | 5 |

# Table S6: The topological characteristic of 21 nodes in the PPI network of gastric cancer-related genes.

| **Gene** | **BetweennessCentrality** | **ClosenessCentrality** | **Degree** |
| --- | --- | --- | --- |
| TIMP1 | 0.02073219 | 0.577922078 | 33 |
| COL1A1 | 0.074875377 | 0.723577236 | 58 |
| COL1A2 | 0.053965488 | 0.700787402 | 56 |
| COL3A1 | 0.046794558 | 0.674242424 | 52 |
| FBN1 | 0.055847529 | 0.613793103 | 41 |
| FN1 | 0.107736133 | 0.700787402 | 54 |
| COL5A2 | 0.031033159 | 0.644927536 | 47 |
| COL5A1 | 0.017681619 | 0.626760563 | 43 |
| COL11A1 | 0.013667749 | 0.593333333 | 38 |
| COL12A1 | 0.019283385 | 0.581699346 | 37 |
| COL4A2 | 0.031269321 | 0.570512821 | 32 |
| SPARC | 0.012967698 | 0.581699346 | 33 |
| LUM | 0.024939894 | 0.618055556 | 43 |
| COL4A1 | 0.018697041 | 0.605442177 | 37 |
| BGN | 0.033707873 | 0.644927536 | 45 |
| THBS1 | 0.018923771 | 0.559748428 | 30 |
| PDGFRB | 0.078309062 | 0.622377622 | 38 |
| THBS2 | 0.049152698 | 0.644927536 | 45 |
| TGFB2 | 0.012700954 | 0.532934132 | 23 |
| SERPINE1 | 0.030440578 | 0.532934132 | 24 |
| CDH11 | 0.047161233 | 0.536144578 | 23 |

# Table S7: The KEGG signaling pathways of cluster I genes in the PPI network of gastric cancer-related genes.

| **ID** | **Description** | **GeneRatio** | **BgRatio** | **pvalue** | **p.adjust** | **qvalue** | **geneID** | **Count** |
| --- | --- | --- | --- | --- | --- | --- | --- | --- |
| hsa04974 | Protein digestion and absorption | 11/23 | 103/8661 | 4.68E-16 | 2.95E-14 | 2.07E-14 | COL4A1/COL5A1/COL1A1/COL10A1/COL4A2/COL8A1/COL12A1/COL3A1/COL1A2/COL6A3/COL11A1 | 11 |
| hsa04512 | ECM-receptor interaction | 9/23 | 89/8661 | 6.14E-13 | 1.93E-11 | 1.36E-11 | COL4A1/COL1A1/FN1/COL4A2/ITGA11/THBS2/THBS1/COL1A2/COL6A3 | 9 |
| hsa04510 | Focal adhesion | 10/23 | 203/8661 | 3.52E-11 | 7.39E-10 | 5.19E-10 | COL4A1/COL1A1/FN1/COL4A2/ITGA11/PDGFRB/THBS2/THBS1/COL1A2/COL6A3 | 10 |
| hsa04933 | AGE-RAGE signaling pathway in diabetic complications | 8/23 | 100/8661 | 1.01E-10 | 1.59E-09 | 1.12E-09 | COL4A1/COL1A1/FN1/TGFB2/COL4A2/COL3A1/COL1A2/SERPINE1 | 8 |
| hsa05165 | Human papillomavirus infection | 10/23 | 331/8661 | 4.27E-09 | 5.38E-08 | 3.77E-08 | COL4A1/COL1A1/FN1/COL4A2/ITGA11/PDGFRB/THBS2/THBS1/COL1A2/COL6A3 | 10 |
| hsa05146 | Amoebiasis | 7/23 | 102/8661 | 5.36E-09 | 5.63E-08 | 3.95E-08 | COL4A1/COL1A1/FN1/TGFB2/COL4A2/COL3A1/COL1A2 | 7 |
| hsa04151 | PI3K-Akt signaling pathway | 10/23 | 359/8661 | 9.34E-09 | 8.41E-08 | 5.90E-08 | COL4A1/COL1A1/FN1/COL4A2/ITGA11/PDGFRB/THBS2/THBS1/COL1A2/COL6A3 | 10 |
| hsa05205 | Proteoglycans in cancer | 6/23 | 205/8661 | 1.18E-05 | 9.28E-05 | 6.51E-05 | LUM/COL1A1/FN1/TGFB2/THBS1/COL1A2 | 6 |
| hsa04926 | Relaxin signaling pathway | 5/23 | 129/8661 | 1.84E-05 | 0.000128806 | 9.04E-05 | COL4A1/COL1A1/COL4A2/COL3A1/COL1A2 | 5 |
| hsa05144 | Malaria | 3/23 | 50/8661 | 0.000295596 | 0.001862252 | 0.001306844 | TGFB2/THBS2/THBS1 | 3 |
| hsa05222 | Small cell lung cancer | 3/23 | 92/8661 | 0.001761122 | 0.009600043 | 0.006736872 | COL4A1/FN1/COL4A2 | 3 |
| hsa05415 | Diabetic cardiomyopathy | 4/23 | 203/8661 | 0.00182858 | 0.009600043 | 0.006736872 | COL1A1/TGFB2/COL3A1/COL1A2 | 4 |
| hsa04350 | TGF-beta signaling pathway | 3/23 | 108/8661 | 0.002784926 | 0.013496181 | 0.009471004 | TGFB2/FBN1/THBS1 | 3 |
| hsa04611 | Platelet activation | 3/23 | 124/8661 | 0.004115056 | 0.018517752 | 0.012994914 | COL1A1/COL3A1/COL1A2 | 3 |

# Table S8: The clustering of PPI networks of gastric cancer-related genes.

| **Cluster** | **PPI** | **Score** | **Nodes** | **Edges** | **Node IDs** |
| --- | --- | --- | --- | --- | --- |
| 1 | 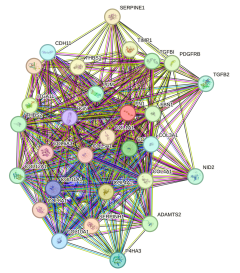 | 22.571 | 29 | 316 | COL4A1, COL5A1, LUM, ADAMTS2, NID2, COL1A1, TIMP1, CDH11, SERPINH1, BGN, FN1, COL10A1, TGFB2, COL4A2, ITGA11, COL8A1, COL12A1, FBN1, VCAN, P4HA3, PDGFRB, THBS2, COL3A1, THBS1, COL1A2, COL6A3, SERPINE1, TGFBI, COL11A1 |
| 2 | 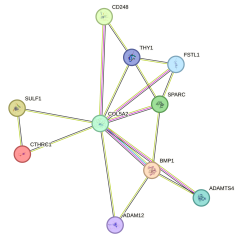 | 3.778 | 10 | 17 | CD248, ADAMTS4, SULF1, THY1, ADAM12, FSTL1, BMP1, COL5A2, SPARC, CTHRC1 |

# Table S9: The top 20 biological processes of depression-related genes.

| **ID** | **Description** | **GeneRatio** | **BgRatio** | **pvalue** | **p.adjust** | **qvalue** | **geneID** | **Count** |
| --- | --- | --- | --- | --- | --- | --- | --- | --- |
| GO:0050804 | modulation of chemical synaptic transmission | 102/533 | 489/18870 | 1.31E-59 | 4.45E-56 | 1.69E-56 | SLC6A4/HTR2A/BDNF/GRIN1/GRIN2A/MECP2/DRD2/APOE/GRIN2B/TNF/GRIA1/MAPT/DISC1/PTEN/DLG4/DRD3/NRXN1/IL1B/NTRK2/ACE/RELN/INS/PRKN/HTR1B/KRAS/DRD5/FMR1/SNCA/APP/OXTR/DRD1/GRIA2/SNAP25/PRNP/JAK2/NGF/GRM7/GRIK2/GRIA3/HCRT/GRM5/CNR1/GHRL/GSK3B/SYN1/ADIPOQ/LRRK2/PSEN1/GFAP/DTNBP1/CHRM2/CAMK2B/GRM1/SYP/TACR1/CAMK2A/NEFL/CCL2/SLC6A1/ATP2A2/KIT/GRIA4/MAPK1/SLC1A1/PINK1/SYNE1/S100B/SLC1A3/PRRT2/NTF3/CRHR2/NLGN3/ADRA2A/ADORA2A/SQSTM1/LGI1/GRM2/GRM3/ACHE/VGF/EDN1/HOMER1/CALB2/CHMP2B/ABL1/ADRB2/PAFAH1B1/NTRK1/PTGS2/GRIK4/PRKCG/BCHE/CALB1/MAPK3/SNCAIP/UNC13A/NGFR/GRIN2D/BCR/CHRNB2/KISS1/SLC12A2 | 102 |
| GO:0099177 | regulation of trans-synaptic signaling | 102/533 | 490/18870 | 1.61E-59 | 4.45E-56 | 1.69E-56 | SLC6A4/HTR2A/BDNF/GRIN1/GRIN2A/MECP2/DRD2/APOE/GRIN2B/TNF/GRIA1/MAPT/DISC1/PTEN/DLG4/DRD3/NRXN1/IL1B/NTRK2/ACE/RELN/INS/PRKN/HTR1B/KRAS/DRD5/FMR1/SNCA/APP/OXTR/DRD1/GRIA2/SNAP25/PRNP/JAK2/NGF/GRM7/GRIK2/GRIA3/HCRT/GRM5/CNR1/GHRL/GSK3B/SYN1/ADIPOQ/LRRK2/PSEN1/GFAP/DTNBP1/CHRM2/CAMK2B/GRM1/SYP/TACR1/CAMK2A/NEFL/CCL2/SLC6A1/ATP2A2/KIT/GRIA4/MAPK1/SLC1A1/PINK1/SYNE1/S100B/SLC1A3/PRRT2/NTF3/CRHR2/NLGN3/ADRA2A/ADORA2A/SQSTM1/LGI1/GRM2/GRM3/ACHE/VGF/EDN1/HOMER1/CALB2/CHMP2B/ABL1/ADRB2/PAFAH1B1/NTRK1/PTGS2/GRIK4/PRKCG/BCHE/CALB1/MAPK3/SNCAIP/UNC13A/NGFR/GRIN2D/BCR/CHRNB2/KISS1/SLC12A2 | 102 |
| GO:0023061 | signal release | 97/533 | 484/18870 | 6.55E-55 | 1.20E-51 | 4.58E-52 | SLC6A4/HTR2A/DRD2/TNF/HTR1A/IL6/CRH/DRD3/HTR2C/NRXN1/IL1B/POMC/INS/PRKN/LEP/CRHR1/HTR1B/CREB1/IFNG/FMR1/SNCA/MAOB/GDNF/OXTR/OPRM1/SNAP25/JAK2/HCRT/CLOCK/CHD7/CNR1/GHRL/PCLO/CRY1/ADCY5/PER2/SLC18A2/SYN1/PLA2G6/ADIPOQ/LRRK2/PSEN1/TSPO/CHRNA4/DTNBP1/P2RX7/KDM5B/SYP/HLA-DRB1/TACR1/TARDBP/CAMK2A/GAL/ATP2A2/NR1D1/ADCYAP1/PINK1/ACSL4/CRY2/REN/PRRT2/CASR/PPARG/CAMK2G/TRH/AGT/CRHBP/AGTR1/ADRA2A/ADORA2A/CYP19A1/PARK7/GRM2/REST/VGF/IL1RN/ABCC8/GJA1/EDN1/PPT1/ABCA1/PTGS2/NOS2/IL1A/PRKCG/TCF7L2/TFAP2B/PLA2G4A/PRKAR1A/MIF/BMP6/SNCAIP/UNC13A/GNRHR/CCL5/CHRNB2/KISS1 | 97 |
| GO:0009410 | response to xenobiotic stimulus | 81/533 | 434/18870 | 3.23E-43 | 4.44E-40 | 1.69E-40 | SLC6A4/HTR2A/GRIN1/GRIN2A/DRD2/SLC6A3/TNF/GRIA1/MTHFR/TH/SLC6A2/DRD3/IL1B/CTNNB1/IL10/ACE/CYP2D6/HTR1B/CREB1/TP53/SNCA/RORA/MAOB/OXTR/DRD1/PRNP/NOS1/SOD1/CYP2C19/ADIPOQ/TSPO/ABCB1/NPC1/PAX6/GAD2/P2RX7/ITGB3/SST/FOS/CYP3A4/GAL/PDE4B/SLC1A1/TGFBR2/SLC1A2/NPAS2/SLC1A3/SMPD1/REN/CYP2B6/CRHBP/ADORA2A/GRM2/REST/PTH/ABCC8/CAT/FGF8/EDN1/BCL2/ABL1/PDE4A/ABCA1/NTRK1/PTGS2/SLC29A3/NOS2/SOD2/BCHE/STAT1/ENG/TFAP2B/LTA/AMH/MMP2/ABCC9/CYP2C9/CALR/UCHL1/GSTM1/CCND1 | 81 |
| GO:0050890 | cognition | 67/533 | 317/18870 | 2.67E-39 | 2.94E-36 | 1.12E-36 | SLC6A4/HTR2A/BDNF/GRIN1/GRIN2A/MECP2/DRD2/APOE/GRIN2B/TNF/GRIA1/TH/CRH/MAPT/PTEN/DLG4/DRD3/NRXN1/NTRK2/RELN/INS/PRKN/CREB1/KRAS/DRD5/APP/OXTR/DRD1/SNAP25/PRNP/NGF/TUSC3/GRM5/DBH/CHD7/CNR1/HTT/PSEN1/CHRNA4/PAX6/FOS/TACR1/SLC6A1/KIT/MAPK1/SLC1A1/S100B/INSR/NTF3/NLGN3/CRHBP/PPP1R1B/ABCC8/CHMP2B/EGFR/TREM2/ABL1/PPT1/LINS1/PAFAH1B1/NTRK1/PTGS2/PRKCG/BCHE/CALB1/FGF13/CHRNB2 | 67 |
| GO:0042391 | regulation of membrane potential | 76/533 | 440/18870 | 4.58E-38 | 4.21E-35 | 1.60E-35 | SLC6A4/GRIN1/GRIN2A/MECP2/DRD2/DRD4/GRIN2B/TNF/CACNA1C/GRIA1/MAPT/PTEN/DLG4/NRXN1/NTRK2/RELN/PRKN/FLNA/FMR1/SNCA/MTOR/APP/AKT1/DRD1/GRIA2/HTR3A/GRIK2/GRIA3/HCRT/GRM5/SOD1/CNR1/GHRL/CD36/GSK3B/SCN8A/ATP1A3/LRRK2/PSEN1/TSPO/CHRNA4/P2RX7/PANK2/GRM1/GABRB3/TACR1/GABRA1/VCP/ATP2A2/GRIA4/PINK1/SYNE1/GABRG2/NLGN3/ADORA2A/PARK7/CACNA1H/GJA1/EDN1/SCN10A/BCL2/TREM2/ABL1/GABRA3/TBX5/ADRB2/GRIK4/SOD2/HCN1/NOS1AP/TRPV1/FGF13/ABCC9/GRIN2D/HTR3B/CHRNB2 | 76 |
| GO:0007611 | learning or memory | 60/533 | 274/18870 | 3.72E-36 | 2.93E-33 | 1.12E-33 | SLC6A4/HTR2A/BDNF/GRIN1/GRIN2A/MECP2/DRD2/APOE/GRIN2B/GRIA1/TH/CRH/MAPT/PTEN/DLG4/DRD3/NRXN1/NTRK2/RELN/PRKN/CREB1/KRAS/DRD5/APP/OXTR/DRD1/SNAP25/PRNP/NGF/GRM5/DBH/CNR1/HTT/PSEN1/PAX6/FOS/TACR1/SLC6A1/KIT/MAPK1/SLC1A1/S100B/INSR/NTF3/NLGN3/CRHBP/PPP1R1B/ABCC8/EGFR/TREM2/ABL1/PPT1/PAFAH1B1/NTRK1/PTGS2/PRKCG/BCHE/CALB1/FGF13/CHRNB2 | 60 |
| GO:0051347 | positive regulation of transferase activity | 71/533 | 414/18870 | 2.39E-35 | 1.65E-32 | 6.26E-33 | APOE/DRD4/TNF/MAPT/PTEN/DLG4/IL1B/NTRK2/CTNNB1/ACE/RELN/INS/LEP/FGFR3/IFNG/SNCA/FGFR1/AKT1/IGF1/PRNP/JAK2/MAP2K1/GRM5/FGFR2/PDGFRB/ADIPOQ/LRRK2/ITGB3/NRG1/HLA-DRB1/KIT/MAPK1/ADCYAP1/VEGFA/TGFBR2/SIRT1/TGFB1/PINK1/INSR/NTF3/CSF1R/ERBB4/AGT/TLR4/AGTR1/GH1/IGF2/ADRA2A/PARK7/APOA1/IL4/EDN1/FBXW7/CALCA/EGFR/TREM2/ABL1/ADRB2/IGF1R/NTRK1/IL18/POLG2/FGF2/EGF/PPP2CA/NTRK3/MAPK3/CCL5/PDGFB/CLU/CCND1 | 71 |
| GO:0046879 | hormone secretion | 62/533 | 308/18870 | 4.43E-35 | 2.71E-32 | 1.03E-32 | DRD2/TNF/HTR1A/IL6/CRH/HTR2C/IL1B/POMC/INS/PRKN/LEP/CRHR1/CREB1/IFNG/SNAP25/JAK2/CLOCK/CHD7/CNR1/GHRL/PCLO/CRY1/ADCY5/PER2/SLC18A2/PLA2G6/ADIPOQ/TSPO/KDM5B/HLA-DRB1/TACR1/TARDBP/GAL/NR1D1/ADCYAP1/ACSL4/CRY2/REN/CASR/PPARG/CAMK2G/TRH/AGT/CRHBP/AGTR1/ADRA2A/CYP19A1/PARK7/REST/VGF/IL1RN/ABCC8/GJA1/EDN1/NOS2/TCF7L2/TFAP2B/PRKAR1A/BMP6/GNRHR/CCL5/KISS1 | 62 |
| GO:0042886 | amide transport | 65/533 | 354/18870 | 3.18E-34 | 1.75E-31 | 6.68E-32 | DRD2/TNF/IL6/CRH/HTR2C/IL1B/NTRK2/INS/PRKN/LEP/CRHR1/IFNG/SNCA/SNAP25/JAK2/GRM7/CLOCK/CHD7/CNR1/GHRL/PCLO/ADCY5/PER2/PLA2G6/PSEN1/ABCB1/DTNBP1/P2RX7/GRM1/HLA-DRB1/TARDBP/GAL/NR1D1/SLC1A1/ADCYAP1/SLC1A2/ACSL4/SLC1A3/CASR/CAMK2G/TRH/PSAP/CRHBP/ADRA2A/ADORA2A/PARK7/GRM2/REST/AVP/VGF/IL1RN/ABCC8/GJA1/EDN1/AVPR1A/ABCA1/NOS2/TCF7L2/TFAP2B/TRPV1/PRKAR1A/SLC17A7/ABCA13/CCL5/KISS1 | 65 |
| GO:0009914 | hormone transport | 62/533 | 319/18870 | 3.77E-34 | 1.89E-31 | 7.19E-32 | DRD2/TNF/HTR1A/IL6/CRH/HTR2C/IL1B/POMC/INS/PRKN/LEP/CRHR1/CREB1/IFNG/SNAP25/JAK2/CLOCK/CHD7/CNR1/GHRL/PCLO/CRY1/ADCY5/PER2/SLC18A2/PLA2G6/ADIPOQ/TSPO/KDM5B/HLA-DRB1/TACR1/TARDBP/GAL/NR1D1/ADCYAP1/ACSL4/CRY2/REN/CASR/PPARG/CAMK2G/TRH/AGT/CRHBP/AGTR1/ADRA2A/CYP19A1/PARK7/REST/VGF/IL1RN/ABCC8/GJA1/EDN1/NOS2/TCF7L2/TFAP2B/PRKAR1A/BMP6/GNRHR/CCL5/KISS1 | 62 |
| GO:0035249 | synaptic transmission, glutamatergic | 40/533 | 111/18870 | 1.02E-33 | 4.70E-31 | 1.79E-31 | HTR2A/GRIN1/GRIN2A/DRD2/GRIN2B/TNF/GRIA1/NR3C1/DISC1/DRD3/NRXN1/RELN/PRKN/HTR1B/OXTR/DRD1/GRIA2/GRM7/GRIK2/GRIA3/GRM5/CNR1/LRRK2/PSEN1/DTNBP1/GRM1/CCL2/GRIA4/NLGN3/ADORA2A/GRM2/GRM3/ABCC8/HOMER1/NTRK1/PTGS2/GRIK4/SLC17A7/UNC13A/GRIN2D | 40 |
| GO:0003018 | vascular process in circulatory system | 57/533 | 269/18870 | 1.49E-33 | 6.30E-31 | 2.40E-31 | SLC6A4/HTR2A/APOE/TNF/HTR1A/CRP/ACE/INS/LEP/HTR1B/DRD5/OXTR/DRD1/NOS1/DBH/SOD1/CD36/PER2/CBS/ABCB1/SLC2A1/GCH1/NOS3/TACR1/HTR1D/SLC6A1/HBB/SLC1A1/VEGFA/AVPR1B/TGFB1/SLC1A2/SLC1A3/INSR/CASR/AGT/AGTR1/ADRA2A/ADORA2A/AVP/ABCC8/EDN1/AVPR1A/FAAH/CALCA/ABL1/ADRB2/PTGS2/SOD2/EDNRA/MMP2/ABCC9/BMP6/LEPR/HTR7/BCR/SLC12A2 | 57 |
| GO:0046883 | regulation of hormone secretion | 55/533 | 251/18870 | 3.33E-33 | 1.31E-30 | 4.99E-31 | DRD2/TNF/HTR1A/IL6/CRH/HTR2C/IL1B/POMC/INS/PRKN/LEP/CRHR1/CREB1/IFNG/SNAP25/JAK2/CLOCK/CHD7/CNR1/GHRL/CRY1/ADCY5/PER2/PLA2G6/ADIPOQ/TSPO/KDM5B/HLA-DRB1/TACR1/TARDBP/GAL/NR1D1/ADCYAP1/ACSL4/CRY2/REN/CASR/PPARG/TRH/AGT/CRHBP/AGTR1/ADRA2A/CYP19A1/REST/ABCC8/GJA1/EDN1/NOS2/TCF7L2/TFAP2B/PRKAR1A/BMP6/CCL5/KISS1 | 55 |
| GO:0043523 | regulation of neuron apoptotic process | 53/533 | 232/18870 | 5.40E-33 | 1.98E-30 | 7.55E-31 | HTR2A/BDNF/MECP2/APOE/TNF/NR3C1/WFS1/NTRK2/CTNNB1/NR4A2/IL10/PRKN/TP53/KRAS/SNCA/GDNF/PRNP/JAK2/NGF/GRIK2/SOD1/PSEN1/NEFL/ATM/CCL2/PIK3CA/SLC1A1/SIRT1/PINK1/GRN/NTF3/ADORA2A/PARK7/SIGMAR1/ATP13A2/PPARGC1A/FGF8/WNT1/TERT/FBXW7/BCL2/TREM2/ABL1/PPT1/NTRK1/SOD2/PRKCG/TFAP2B/CNTF/MSH2/CLU/MAG/CCND1 | 53 |
| GO:0051402 | neuron apoptotic process | 57/533 | 278/18870 | 9.65E-33 | 3.32E-30 | 1.26E-30 | HTR2A/BDNF/MECP2/APOE/TNF/NR3C1/WFS1/NTRK2/CTNNB1/NR4A2/IL10/PRKN/TP53/KRAS/SNCA/APP/GDNF/PRNP/JAK2/NGF/GRIK2/SOD1/PSEN1/NEFL/ATM/CCL2/PIK3CA/SLC1A1/SIRT1/PINK1/GRN/NTF3/ADORA2A/PARK7/SIGMAR1/ATP13A2/PPARGC1A/FAS/FGF8/WNT1/TERT/FBXW7/BCL2/TREM2/ABL1/PPT1/NTRK1/ATN1/SOD2/PRKCG/TFAP2B/CNTF/NGFR/MSH2/CLU/MAG/CCND1 | 57 |
| GO:0033674 | positive regulation of kinase activity | 62/533 | 337/18870 | 1.03E-32 | 3.35E-30 | 1.28E-30 | DRD4/TNF/MAPT/DLG4/IL1B/NTRK2/ACE/RELN/INS/LEP/FGFR3/IFNG/SNCA/FGFR1/AKT1/IGF1/PRNP/JAK2/MAP2K1/GRM5/FGFR2/PDGFRB/ADIPOQ/LRRK2/ITGB3/NRG1/HLA-DRB1/KIT/ADCYAP1/VEGFA/TGFBR2/SIRT1/TGFB1/PINK1/INSR/NTF3/CSF1R/ERBB4/AGT/TLR4/GH1/ADRA2A/PARK7/IL4/EDN1/FBXW7/CALCA/EGFR/TREM2/ABL1/ADRB2/IGF1R/NTRK1/IL18/FGF2/EGF/PPP2CA/NTRK3/CCL5/PDGFB/CLU/CCND1 | 62 |
| GO:0043410 | positive regulation of MAPK cascade | 72/533 | 474/18870 | 2.68E-32 | 8.21E-30 | 3.12E-30 | HTR2A/DRD2/APOE/DRD4/TNF/IL6/HTR2C/IL1B/NTRK2/CTNNB1/INS/LEP/NOTCH1/FGFR3/APP/FGFR1/IGF1/OPRM1/JAK2/MAP2K1/GRM5/SOD1/FGFR2/GHRL/CD36/PDGFRB/AR/LRRK2/P2RX7/ITGB3/NRG1/GRM1/HLA-DRB1/CCL2/KIT/ADCYAP1/VEGFA/AVPR1B/TGFB1/INSR/CASR/NTF3/CSF1R/ERBB4/TLR4/GH1/IGF2/ADRA2A/TBX1/FGF8/HCRTR1/EDN1/FBXW7/EPO/EGFR/TREM2/ABL1/PTPN22/ADRB2/IGF1R/NTRK1/IL1A/ICAM1/FGF2/SOX2/EGF/NTRK3/MIF/MAPK3/CCL5/PDGFB/KISS1 | 72 |
| GO:0015850 | organic hydroxy compound transport | 59/533 | 308/18870 | 3.71E-32 | 1.08E-29 | 4.10E-30 | SLC6A4/HTR2A/MECP2/DRD2/APOE/DRD4/SLC6A3/HTR1A/CRH/SLC6A2/DRD3/POMC/PRKN/LEP/CRHR1/HTR1B/SNCA/MAOB/GDNF/OXTR/DRD1/NOS1/TOR1A/CNR1/GHRL/CD36/SLC18A2/ADIPOQ/TSPO/CHRNA4/NPC1/DTNBP1/ITGB3/APOB/KDM5B/PON1/GAL/SIRT1/PINK1/REN/PPARG/AGT/AGTR1/ADRA2A/ADORA2A/CYP19A1/PARK7/GRM2/APOA1/ACTB/TREM2/ABCA1/SLC29A3/EGF/STAR/BMP6/ABCA13/CLU/CHRNB2 | 59 |
| GO:0007626 | locomotory behavior | 49/533 | 201/18870 | 5.54E-32 | 1.53E-29 | 5.80E-30 | MECP2/DRD2/APOE/DRD4/SLC6A3/GAD1/TH/CRH/PTEN/DLG4/DRD3/HTR2C/NR4A2/RELN/PRKN/SNCA/MTOR/APP/GDNF/DRD1/SNAP25/GRM5/DBH/CHD7/SOD1/ADCY5/SLC18A2/LRRK2/NPC1/GRM1/SLC1A1/IDO1/TRH/ADORA2A/CDH23/CLN6/PARK7/AVP/PPP1R1B/FIG4/PPT1/PAFAH1B1/DPP4/SOD2/CRBN/CALB1/GRIN2D/UCHL1/CHRNB2 | 49 |
| GO:0018108 | peptidyl-tyrosine phosphorylation | 56/533 | 276/18870 | 6.33E-32 | 1.66E-29 | 6.32E-30 | HTR2A/TNF/IL6/DLG4/ACE/RELN/LEP/FGFR3/IFNG/TP53/MTOR/APP/FGFR1/IGF1/PRNP/JAK2/GRM5/FGFR2/CD36/PDGFRB/ADIPOQ/ITGB3/KIT/IL2/VEGFA/TGFB1/INSR/NTF3/CSF1R/ERBB4/AGT/GH1/IGF2/ADRA2A/TNFRSF1A/IL4/FGF8/FBXW7/EPO/EGFR/TREM2/ABL1/IGF1R/NTRK1/IL18/EGF/PPP2CA/MIF/BMP6/MAPK3/CNTF/CSF3/CCL5/PDGFB/SOCS1/IL13 | 56 |

# Table S10: The top 20 KEGG signaling pathways of depression-related genes.

| **ID** | **Description** | **GeneRatio** | **BgRatio** | **pvalue** | **p.adjust** | **qvalue** | **geneID** | **Count** |
| --- | --- | --- | --- | --- | --- | --- | --- | --- |
| hsa04080 | Neuroactive ligand-receptor interaction | 82/439 | 367/8645 | 4.00E-32 | 1.23E-29 | 4.97E-30 | HTR2A/GRIN1/GRIN2A/DRD2/DRD4/GRIN2B/HTR1A/GRIA1/NR3C1/CRH/PRL/DRD3/HTR2C/POMC/LEP/CRHR1/OXT/HTR1B/NPY/DRD5/OXTR/OPRM1/DRD1/GRIA2/GRM7/GRIK2/GRIA3/HCRT/GRM5/CNR1/GHRL/CCK/TSPO/CHRNA4/PDYN/P2RX7/CHRM2/SST/GRM1/GABRB3/TACR1/GABRA1/GAL/HTR1D/TAC1/TACR3/GRIA4/ADCYAP1/AVPR1B/GABRG2/HTR6/CRHR2/TRH/AGT/GNRH1/AGTR1/GH1/ADRA2A/ADORA2A/GPR50/GRM2/GRM3/AVP/PTH/VGF/HCRTR1/EDN1/AVPR1A/CALCA/GABRA3/ADRB2/GRIK4/EDNRA/PYY/PLG/TRPV1/GNRHR/GRIN2D/LEPR/HTR7/CHRNB2/KISS1 | 82 |
| hsa04024 | cAMP signaling pathway | 54/439 | 225/8645 | 9.46E-23 | 1.46E-20 | 5.87E-21 | BDNF/GRIN1/GRIN2A/DRD2/GRIN2B/CACNA1C/HTR1A/GRIA1/CRH/POMC/GLI3/CRHR1/OXT/HTR1B/CREB1/NPY/DRD5/AKT1/OXTR/DRD1/GRIA2/GRIA3/MAP2K1/GHRL/ADCY5/ATP1A3/GNAS/CHRM2/SST/CAMK2B/FOS/CAMK2A/HTR1D/PIK3CA/ATP2A2/GRIA4/MAPK1/PDE4B/ADCYAP1/PIK3R1/HTR6/CRHR2/CAMK2G/RAC1/ADORA2A/PPP1R1B/EDN1/PDE4A/ADRB2/EDNRA/PPARA/AMH/MAPK3/GRIN2D | 54 |
| hsa04151 | PI3K-Akt signaling pathway | 66/439 | 359/8645 | 8.35E-21 | 8.58E-19 | 3.46E-19 | BDNF/IL6/PRL/PTEN/NTRK2/RELN/INS/CREB1/GNB3/FGFR3/TP53/KRAS/MTOR/FGFR1/AKT1/IGF1/GDNF/JAK2/NGF/MAP2K1/FGFR2/GSK3B/PDGFRB/GNB1/CHRM2/ITGB3/NOS3/PIK3CA/KIT/NRAS/MAPK1/IL2/VEGFA/COL4A1/PIK3R1/INSR/NTF3/CSF1R/ERBB4/RAC1/COL9A3/TLR4/GH1/IGF2/IL4/FGF8/COL9A2/BRCA1/IL2RA/BCL2/IFNA1/EPO/COL9A1/EGFR/IGF1R/NTRK1/FGF2/EGF/PPP2CA/MAPK3/NGFR/CSF3/PDGFB/PPP2R2B/IL2RB/CCND1 | 66 |
| hsa05022 | Pathways of neurodegeneration - multiple diseases | 74/439 | 476/8645 | 7.64E-19 | 5.88E-17 | 2.37E-17 | BDNF/GRIN1/GRIN2A/SLC6A3/GRIN2B/TNF/CACNA1C/IL6/GRIA1/MAPT/DLG4/IL1B/CTNNB1/PRKN/KRAS/SNCA/MTOR/APP/C9orf72/XBP1/GRIA2/PRNP/GRIA3/MAP2K1/NOS1/GRM5/SOD1/HTT/GSK3B/LRRK2/PSEN1/PDYN/DCTN1/ATXN2/CAMK2B/GRM1/TARDBP/CAMK2A/VCP/RYR1/NEFL/ATXN3/ATP2A2/GRIA4/NRAS/MAPK1/PINK1/FUS/CAMK2G/RAC1/SQSTM1/PARK7/SIGMAR1/FAS/TNFRSF1A/SLC25A4/CAT/FIG4/WNT1/BCL2/CHMP2B/FZD6/PTGS2/NOS2/IL1A/PRKCG/NDUFS4/OPTN/MAPK3/SNCAIP/GRIN2D/UCHL1/TBK1/DNAH8 | 74 |
| hsa04020 | Calcium signaling pathway | 52/439 | 253/8645 | 1.05E-18 | 6.45E-17 | 2.60E-17 | HTR2A/GRIN1/GRIN2A/GRIN2B/CACNA1C/HTR2C/NTRK2/FGFR3/DRD5/FGFR1/GDNF/OXTR/DRD1/NGF/NOS1/GRM5/FGFR2/PDGFRB/GNAS/P2RX7/CHRM2/NOS3/CAMK2B/GRM1/TACR1/CAMK2A/RYR1/ATP2A2/TACR3/VEGFA/AVPR1B/HTR6/CAMK2G/ERBB4/AGTR1/ADORA2A/CACNA1H/SLC25A4/FGF8/AVPR1A/EGFR/ADRB2/NTRK1/NOS2/PRKCG/EDNRA/FGF2/EGF/NTRK3/GRIN2D/PDGFB/HTR7 | 52 |
| hsa04933 | AGE-RAGE signaling pathway in diabetic complications | 31/439 | 100/8645 | 6.92E-17 | 3.55E-15 | 1.43E-15 | TNF/IL6/IL1B/KRAS/AKT1/JAK2/NOS3/CCL2/PIK3CA/NRAS/MAPK1/CXCL8/VEGFA/TGFBR2/STAT3/TGFB1/COL4A1/PIK3R1/RAC1/AGT/SERPINE1/AGTR1/F3/EDN1/BCL2/IL1A/ICAM1/STAT1/MMP2/MAPK3/CCND1 | 31 |
| hsa04726 | Serotonergic synapse | 33/439 | 115/8645 | 9.05E-17 | 3.85E-15 | 1.55E-15 | SLC6A4/HTR2A/TPH2/MAOA/CACNA1C/HTR1A/TPH1/HTR2C/CYP2D6/HTR1B/GNB3/KRAS/APP/MAOB/HTR3A/MAP2K1/ADCY5/CYP2C19/SLC18A2/GNB1/GNAS/GABRB3/HTR1D/NRAS/MAPK1/HTR6/PTGS2/PRKCG/PLA2G4A/MAPK3/CYP2C9/HTR3B/HTR7 | 33 |
| hsa05031 | Amphetamine addiction | 26/439 | 69/8645 | 1.00E-16 | 3.85E-15 | 1.55E-15 | GRIN1/GRIN2A/MAOA/SLC6A3/GRIN2B/CACNA1C/GRIA1/TH/CREB1/MAOB/DRD1/GRIA2/GRIA3/ADCY5/SLC18A2/GNAS/PDYN/CAMK2B/FOS/CAMK2A/GRIA4/SIRT1/CAMK2G/PPP1R1B/PRKCG/GRIN2D | 26 |
| hsa01521 | EGFR tyrosine kinase inhibitor resistance | 27/439 | 79/8645 | 4.55E-16 | 1.56E-14 | 6.28E-15 | IL6/PTEN/FGFR3/KRAS/MTOR/AKT1/IGF1/JAK2/MAP2K1/FGFR2/GSK3B/PDGFRB/NRG1/PIK3CA/NRAS/MAPK1/VEGFA/STAT3/PIK3R1/BCL2/EGFR/IGF1R/PRKCG/FGF2/EGF/MAPK3/PDGFB | 27 |
| hsa04728 | Dopaminergic synapse | 34/439 | 132/8645 | 1.15E-15 | 3.53E-14 | 1.42E-14 | COMT/GRIN2A/DRD2/DRD4/MAOA/SLC6A3/GRIN2B/CACNA1C/GRIA1/TH/DRD3/CREB1/GNB3/DRD5/AKT1/MAOB/DRD1/GRIA2/GRIA3/CLOCK/ADCY5/GSK3B/SLC18A2/GNB1/GNAS/CAMK2B/FOS/CAMK2A/GRIA4/CAMK2G/PPP1R1B/PRKCG/PPP2CA/PPP2R2B | 34 |
| hsa05215 | Prostate cancer | 29/439 | 97/8645 | 2.11E-15 | 5.90E-14 | 2.38E-14 | PTEN/CTNNB1/INS/CREB1/TP53/KRAS/MTOR/FGFR1/AKT1/IGF1/MAP2K1/FGFR2/GSK3B/PDGFRB/AR/PLAU/PIK3CA/NRAS/MAPK1/PIK3R1/MMP9/BCL2/EGFR/IGF1R/TCF7L2/EGF/MAPK3/PDGFB/CCND1 | 29 |
| hsa05417 | Lipid and atherosclerosis | 43/439 | 215/8645 | 3.62E-15 | 9.28E-14 | 3.74E-14 | TNF/IL6/IL1B/TP53/KRAS/AKT1/XBP1/JAK2/CD36/GSK3B/NOS3/CAMK2B/APOB/FOS/CAMK2A/CCL2/PIK3CA/NRAS/MAPK1/CXCL8/STAT3/PIK3R1/PPARG/CAMK2G/RAC1/CYP2B6/TLR4/NLRP3/FAS/APOA1/TNFRSF1A/MMP9/BCL2/IFNA1/SELP/ABCA1/IL18/SOD2/ICAM1/MAPK3/CYP2C9/CCL5/TBK1 | 43 |
| hsa05030 | Cocaine addiction | 21/439 | 49/8645 | 4.31E-15 | 1.02E-13 | 4.12E-14 | BDNF/GRIN1/GRIN2A/DRD2/MAOA/SLC6A3/GRIN2B/TH/DLG4/CREB1/MAOB/DRD1/GRIA2/ADCY5/SLC18A2/GNAS/PDYN/GRM2/GRM3/PPP1R1B/GRIN2D | 21 |
| hsa04066 | HIF-1 signaling pathway | 30/439 | 109/8645 | 8.31E-15 | 1.83E-13 | 7.37E-14 | IL6/INS/IFNG/MTOR/AKT1/IGF1/MAP2K1/SLC2A1/NOS3/CAMK2B/CAMK2A/PIK3CA/MAPK1/VEGFA/STAT3/PIK3R1/INSR/CAMK2G/SERPINE1/TLR4/HMOX1/EDN1/BCL2/EPO/EGFR/IGF1R/NOS2/PRKCG/EGF/MAPK3 | 30 |
| hsa04713 | Circadian entrainment | 28/439 | 97/8645 | 1.77E-14 | 3.63E-13 | 1.46E-13 | GRIN1/GRIN2A/GRIN2B/CACNA1C/GRIA1/CREB1/GNB3/GRIA2/GRIA3/NOS1/PER3/ADCY5/PER2/GNB1/GNAS/CAMK2B/FOS/CAMK2A/RYR1/GRIA4/MAPK1/ADCYAP1/CAMK2G/CACNA1H/PRKCG/NOS1AP/MAPK3/GRIN2D | 28 |
| hsa04724 | Glutamatergic synapse | 30/439 | 115/8645 | 4.02E-14 | 7.75E-13 | 3.12E-13 | GRIN1/GRIN2A/GRIN2B/CACNA1C/GRIA1/DLG4/GNB3/GRIA2/GRM7/GRIK2/GRIA3/GRM5/ADCY5/GNB1/GNAS/GRM1/GRIA4/MAPK1/SLC1A1/SLC1A2/SLC1A3/GRM2/GRM3/HOMER1/GRIK4/PRKCG/PLA2G4A/SLC17A7/MAPK3/GRIN2D | 30 |
| hsa04014 | Ras signaling pathway | 43/439 | 236/8645 | 1.14E-13 | 2.07E-12 | 8.36E-13 | BDNF/GRIN1/GRIN2A/GRIN2B/NTRK2/INS/GNB3/FGFR3/KRAS/FGFR1/AKT1/IGF1/NGF/MAP2K1/FGFR2/PDGFRB/PLA2G6/GNB1/PIK3CA/KIT/NRAS/MAPK1/VEGFA/PIK3R1/INSR/NTF3/CSF1R/RAC1/IGF2/FGF8/EGFR/ABL1/IGF1R/NTRK1/PRKCG/FGF2/EGF/PLA2G4A/MAPK3/NGFR/PDGFB/TBK1/HTR7 | 43 |
| hsa05205 | Proteoglycans in cancer | 39/439 | 205/8645 | 4.06E-13 | 6.95E-12 | 2.80E-12 | TNF/CTNNB1/ESR1/FLNA/TP53/KRAS/MTOR/FGFR1/AKT1/IGF1/MAP2K1/ITGB3/CAMK2B/CAMK2A/PLAU/PIK3CA/NRAS/MAPK1/VEGFA/STAT3/TGFB1/PIK3R1/CAMK2G/ERBB4/RAC1/TLR4/IGF2/FAS/ACTB/MMP9/WNT1/EGFR/FZD6/IGF1R/PRKCG/FGF2/MMP2/MAPK3/CCND1 | 39 |
| hsa05142 | Chagas disease | 27/439 | 102/8645 | 5.35E-13 | 8.68E-12 | 3.50E-12 | TNF/IL6/IL1B/IL10/ACE/IFNG/AKT1/GNAS/FOS/CCL2/PIK3CA/MAPK1/CXCL8/IL2/TGFBR2/TGFB1/PIK3R1/SERPINE1/TLR4/FAS/TNFRSF1A/NOS2/PPP2CA/MAPK3/CCL5/CALR/PPP2R2B | 27 |
| hsa04010 | MAPK signaling pathway | 48/439 | 301/8645 | 7.27E-13 | 1.12E-11 | 4.52E-12 | BDNF/TNF/CACNA1C/MAPT/IL1B/NTRK2/INS/FGFR3/FLNA/TP53/KRAS/FGFR1/AKT1/IGF1/GDNF/NGF/MAP2K1/FGFR2/PDGFRB/FOS/RPS6KA3/KIT/NRAS/MAPK1/VEGFA/TGFBR2/TGFB1/INSR/NTF3/CSF1R/ERBB4/RAC1/IGF2/CACNA1H/FAS/TNFRSF1A/FGF8/EGFR/IGF1R/NTRK1/IL1A/PRKCG/FGF2/EGF/PLA2G4A/MAPK3/NGFR/PDGFB | 48 |

# Table S11: The topological characteristic of 126 nodes in the PPI network of depression-related genes.

| **Gene** | **BetweennessCentrality** | **ClosenessCentrality** | **Degree** |
| --- | --- | --- | --- |
| INS | 0.032957314 | 0.63020214 | 237 |
| AKT1 | 0.027000426 | 0.630952381 | 229 |
| ALB | 0.028279906 | 0.62426384 | 227 |
| BDNF | 0.025047239 | 0.618436406 | 222 |
| ACTB | 0.020608892 | 0.62133646 | 218 |
| TNF | 0.021284823 | 0.61627907 | 214 |
| IL6 | 0.013959827 | 0.610599078 | 210 |
| TP53 | 0.032065948 | 0.612716763 | 208 |
| IL1B | 0.012191005 | 0.599547511 | 198 |
| CTNNB1 | 0.031557137 | 0.594837262 | 183 |
| FOS | 0.015610896 | 0.583058306 | 182 |
| ESR1 | 0.0131602 | 0.583058306 | 177 |
| EGFR | 0.016770194 | 0.590200445 | 177 |
| STAT3 | 0.009290128 | 0.579234973 | 169 |
| APP | 0.015491172 | 0.581140351 | 167 |
| CREB1 | 0.011353859 | 0.572972973 | 162 |
| MAPK3 | 0.008003239 | 0.575461455 | 160 |
| BCL2 | 0.005439925 | 0.57050592 | 157 |
| IGF1 | 0.005726862 | 0.568060021 | 154 |
| IFNG | 0.005851985 | 0.565031983 | 152 |
| IL10 | 0.004887042 | 0.563230606 | 151 |
| APOE | 0.010373559 | 0.56745182 | 150 |
| PPARG | 0.008504955 | 0.564430245 | 149 |
| AGT | 0.009421891 | 0.556722689 | 148 |
| EGF | 0.004208735 | 0.55613851 | 146 |
| LEP | 0.007789322 | 0.552083333 | 143 |
| GSK3B | 0.012493166 | 0.564430245 | 141 |
| TGFB1 | 0.003722767 | 0.554393305 | 141 |
| PTGS2 | 0.005136291 | 0.557308097 | 138 |
| MMP9 | 0.003485109 | 0.548086867 | 136 |
| CXCL8 | 0.005794314 | 0.548654244 | 135 |
| CCL2 | 0.002924594 | 0.546391753 | 134 |
| NGF | 0.003821984 | 0.554973822 | 134 |
| POMC | 0.008464642 | 0.541368744 | 134 |
| FGF2 | 0.005634345 | 0.549222798 | 133 |
| GFAP | 0.007216282 | 0.557308097 | 133 |
| PTEN | 0.004241407 | 0.55613851 | 132 |
| TLR4 | 0.002795153 | 0.548654244 | 128 |
| KRAS | 0.005232802 | 0.546391753 | 127 |
| MTOR | 0.002878602 | 0.551508845 | 127 |
| NTRK2 | 0.005062251 | 0.544707091 | 120 |
| IL2 | 0.002458244 | 0.540265036 | 118 |
| SIRT1 | 0.005358402 | 0.543032787 | 117 |
| TAC1 | 0.005019409 | 0.53481332 | 117 |
| TH | 0.004930028 | 0.543032787 | 117 |
| DRD2 | 0.008579031 | 0.540265036 | 116 |
| JAK2 | 0.004724297 | 0.53481332 | 115 |
| STAT1 | 0.004316311 | 0.533736153 | 115 |
| GRIN2B | 0.005812146 | 0.53481332 | 114 |
| SNCA | 0.014421001 | 0.54526749 | 113 |
| CCND1 | 0.004878387 | 0.539714868 | 111 |
| SST | 0.004728013 | 0.531062124 | 109 |
| SOX2 | 0.008149165 | 0.53698075 | 107 |
| ACE | 0.005085018 | 0.525793651 | 107 |
| NPY | 0.003640991 | 0.526838966 | 106 |
| CCK | 0.002373037 | 0.526315789 | 104 |
| FGF8 | 0.006010707 | 0.523715415 | 104 |
| DLG4 | 0.004473609 | 0.53 | 103 |
| KIT | 0.003385637 | 0.524752475 | 102 |
| NOTCH1 | 0.006257482 | 0.525793651 | 102 |
| PVALB | 0.006836091 | 0.517073171 | 101 |
| NR3C1 | 0.006058925 | 0.53 | 100 |
| NCAM1 | 0.007153969 | 0.526838966 | 99 |
| PRL | 0.005745752 | 0.524233432 | 99 |
| SLC6A4 | 0.006989303 | 0.520628684 | 98 |
| CDKN2A | 0.00258168 | 0.526315789 | 98 |
| COMT | 0.011136679 | 0.527363184 | 97 |
| MAOB | 0.007391899 | 0.525272547 | 97 |
| MAOA | 0.007313195 | 0.517578125 | 97 |
| MAPK1 | 0.002809139 | 0.531062124 | 97 |
| PIK3CA | 0.006488727 | 0.524752475 | 97 |
| SLC6A3 | 0.005440797 | 0.525793651 | 97 |
| HMOX1 | 0.004029403 | 0.525793651 | 96 |
| MAPT | 0.006142865 | 0.534274194 | 95 |
| PIK3R1 | 0.002383195 | 0.526315789 | 95 |
| SYP | 0.004474034 | 0.531062124 | 95 |
| PPARA | 0.002464674 | 0.523715415 | 93 |
| GAD1 | 0.003116176 | 0.518084066 | 91 |
| AR | 0.004304521 | 0.52114061 | 90 |
| SOD1 | 0.008860413 | 0.526838966 | 90 |
| GAD2 | 0.007280577 | 0.514064016 | 88 |
| PPARGC1A | 0.004013894 | 0.523715415 | 87 |
| IGF2 | 0.002453114 | 0.516569201 | 87 |
| SNAP25 | 0.007700809 | 0.519607843 | 87 |
| AGTR1 | 0.003449656 | 0.508149569 | 86 |
| GRIN2A | 0.004538803 | 0.504761905 | 86 |
| ATM | 0.004211439 | 0.518084066 | 85 |
| HTT | 0.008437023 | 0.527363184 | 85 |
| ESR2 | 0.002921166 | 0.518084066 | 84 |
| HTR2A | 0.0032722 | 0.501893939 | 83 |
| OPRM1 | 0.003151421 | 0.508149569 | 82 |
| BRCA1 | 0.007426112 | 0.514064016 | 81 |
| FGFR1 | 0.002897865 | 0.506692161 | 80 |
| SQSTM1 | 0.003734746 | 0.510597303 | 78 |
| GRIA1 | 0.004411124 | 0.503802281 | 78 |
| DRD1 | 0.002415724 | 0.494402985 | 77 |
| GNAS | 0.005690001 | 0.5028463 | 76 |
| HTR1A | 0.004191294 | 0.498588899 | 76 |
| SOD2 | 0.006621052 | 0.514064016 | 75 |
| APOB | 0.002312786 | 0.505243089 | 73 |
| DNMT1 | 0.002635674 | 0.509615385 | 72 |
| PRKN | 0.004365855 | 0.514563107 | 72 |
| SLC2A1 | 0.006969312 | 0.507662835 | 71 |
| GRIA2 | 0.002993657 | 0.5 | 71 |
| PDYN | 0.002950716 | 0.493482309 | 70 |
| ERBB4 | 0.002320671 | 0.503802281 | 69 |
| IDO1 | 0.005362033 | 0.500472144 | 66 |
| SLC1A2 | 0.002328067 | 0.501893939 | 66 |
| LRRK2 | 0.003469052 | 0.508149569 | 65 |
| GNB1 | 0.003213257 | 0.489833641 | 63 |
| PAX6 | 0.002361927 | 0.499528746 | 60 |
| PARK7 | 0.004426228 | 0.502369668 | 59 |
| MECP2 | 0.00250376 | 0.499528746 | 59 |
| TARDBP | 0.003849667 | 0.499528746 | 57 |
| PINK1 | 0.003190787 | 0.5 | 57 |
| SERPINA1 | 0.003275636 | 0.482695811 | 56 |
| FMR1 | 0.005262921 | 0.492107707 | 56 |
| CACNA1C | 0.003756901 | 0.489381348 | 54 |
| ITGB3 | 0.002557536 | 0.492107707 | 54 |
| DRD4 | 0.002325776 | 0.477477477 | 53 |
| MTHFR | 0.004096311 | 0.491651206 | 53 |
| BRCA2 | 0.002842708 | 0.485792851 | 53 |
| TBP | 0.002925202 | 0.499528746 | 53 |
| VCP | 0.004120238 | 0.493482309 | 53 |
| PLAU | 0.002785631 | 0.485347985 | 52 |
| PRKCG | 0.003354473 | 0.491651206 | 52 |

# Table S12: The clustering of PPI networks of depression-related genes.

| **Cluster** | **PPI** | **Score** | **Nodes** | **Edges** | **Node IDs** |
| --- | --- | --- | --- | --- | --- |
| 1 | 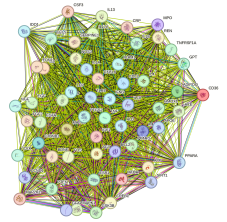 | 49.103 | 59 | 1424 | MAPK1, EGF, IL17A, GFAP, MAPK3, IL10, ESR1, CRP, BDNF, APOE, TNF, IL18, CREB1, SERPINE1, CCND1, IFNG, AGT, BCL2, CCL2, MMP2, ADIPOQ, CCK, MPO, NGF, REN, HMOX1, CXCL8, TLR4, SIRT1, CCL5, PTEN, ACE, FGF2, MMP9, EDN1, PTGS2, MTOR, IDO1, IGF1, LEP, IL13, IL1A, GSK3B, NOS3, MAP2K1, ALB, IL4, PPARG, GPT, APP, IL2, CSF3, PPARA, EGFR, TNFRSF1A, CTNNB1, CD36, FOS, ICAM1 |
| 2 | 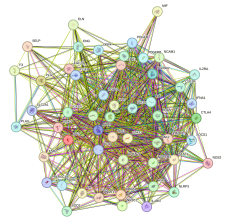 | 27.077 | 53 | 704 | STAT3, IL2RA, JAK2, NLRP3, IL1B, NOTCH1, IL1RN, PLG, AR, IL6, PLAU, BRCA1, SLC2A1, ACTB, KRAS, PDGFB, CSF1R, FGF13, SOD2, NR3C1, CTLA4, POMC, IGF2, ATM, NOS2, NCAM1, AKT1, IFNA1, FGF8, DPP4, MIF, PIK3CA, NTRK1, PDGFRB, STAT1, ENG, SELP, PIK3R1, EPO, TGFBR2, DNMT1, CDKN2A, ELN, SOCS1, SOX2, KIT, ESR2, PPARGC1A, TGFB1, TP53, F3, IGF1R, INS |
| 3 | 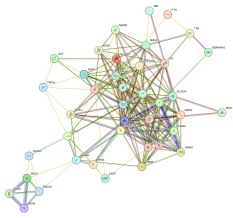 | 11.128 | 40 | 217 | GRM3, TH, GRM1, SYN1, DLG4, TRPV1, FGFR3, NTF3, BRCA2, MAOB, SERPINA1, GRIN2A, SLC1A3, ACHE, SNCA, TTR, NRG1, MSH6, MLH1, SLC6A3, PPP1R1B, MSH2, ERBB4, GRIA4, GRIA1, DRD1, CALB1, PDYN, HTT, PTH, CNTF, GRIA2, SLC1A2, TBP, NOS1, NRXN1, OXT, MOG, SYP, GAD2 |
| 4 | 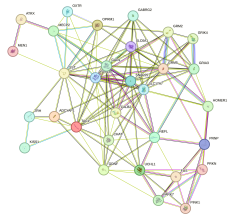 | 8.867 | 31 | 133 | ATRX, PINK1, PRKN, TAC1, GRM2, TRH, SST, HOMER1, GABRG2, SLC17A7, FUS, OXTR, PRNP, UCHL1, GDNF, PVALB, CHAT, SNAP25, OPRM1, KISS1, GRIK4, GAD1, MECP2, CALB2, NEFL, PARK7, GRIA3, SLC6A1, MEN1, GRM5, ADCYAP1 |
| 5 | 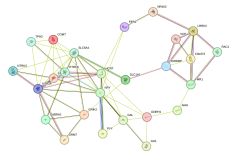 | 5.2 | 26 | 65 | COMT, C9orf72, LRRK2, NPY, HCRT, TARDBP, NTRK3, GABRA1, HTR2A, GNRH1, GRM7, SLC6A4, NR4A2, TPH2, VCP, RAC1, PER2, FMR1, GAL, HTR1B, PYY, GRIK2, SLC1A1, CNR1, AMH, GH1 |
| 6 | 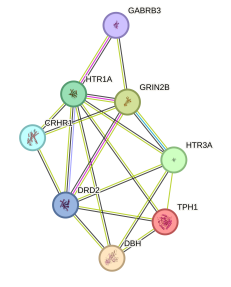 | 5.143 | 8 | 18 | GABRB3, DRD2, HTR1A, CRHR1, DBH, HTR3A, TPH1, GRIN2B |
| 7 | 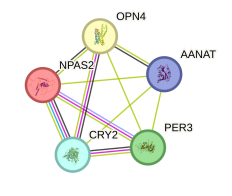 | 5 | 5 | 10 | OPN4, AANAT, NPAS2, CRY2, PER3 |

# Table S13: The KEGG signaling pathways of cluster I genes in the PPI network of depression-related genes.

| **ID** | **Description** | **GeneRatio** | **BgRatio** | **pvalue** | **p.adjust** | **qvalue** | **geneID** | **Count** |
| --- | --- | --- | --- | --- | --- | --- | --- | --- |
| hsa04657 | IL-17 signaling pathway | 14/58 | 94/8661 | 7.95E-16 | 1.38E-13 | 3.71E-14 | MAPK1/IL17A/MAPK3/TNF/IFNG/CCL2/CXCL8/MMP9/PTGS2/IL13/GSK3B/IL4/CSF3/FOS | 14 |
| hsa05215 | Prostate cancer | 14/58 | 97/8661 | 1.26E-15 | 1.38E-13 | 3.71E-14 | MAPK1/EGF/MAPK3/CREB1/CCND1/BCL2/PTEN/MMP9/MTOR/IGF1/GSK3B/MAP2K1/EGFR/CTNNB1 | 14 |
| hsa04933 | AGE-RAGE signaling pathway in diabetic complications | 14/58 | 100/8661 | 1.96E-15 | 1.44E-13 | 3.85E-14 | MAPK1/MAPK3/TNF/SERPINE1/CCND1/AGT/BCL2/CCL2/MMP2/CXCL8/EDN1/IL1A/NOS3/ICAM1/ | 14 |
| hsa05142 | Chagas disease | 14/58 | 102/8661 | 2.61E-15 | 1.44E-13 | 3.85E-14 | MAPK1/MAPK3/IL10/TNF/SERPINE1/IFNG/CCL2/CXCL8/TLR4/CCL5/ACE/IL2/TNFRSF1A/FOS | 14 |
| hsa04066 | HIF-1 signaling pathway | 14/58 | 109/8661 | 6.81E-15 | 3.00E-13 | 8.03E-14 | MAPK1/EGF/MAPK3/SERPINE1/IFNG/BCL2/HMOX1/TLR4/EDN1/MTOR/IGF1/NOS3/MAP2K1/EGFR | 14 |
| hsa05417 | Lipid and atherosclerosis | 17/58 | 215/8661 | 2.21E-14 | 8.09E-13 | 2.17E-13 | MAPK1/MAPK3/TNF/IL18/BCL2/CCL2/CXCL8/TLR4/CCL5/MMP9/GSK3B/NOS3/PPARG/TNFRSF1A/CD36/FOS/ICAM1/ | 17 |
| hsa04151 | PI3K-Akt signaling pathway | 20/58 | 359/8661 | 5.81E-14 | 1.82E-12 | 4.89E-13 | MAPK1/EGF/MAPK3/BDNF/CREB1/CCND1/BCL2/NGF/TLR4/PTEN/FGF2/MTOR/IGF1/GSK3B/NOS3/MAP2K1/IL4/IL2/CSF3/EGFR/ | 20 |
| hsa05418 | Fluid shear stress and atherosclerosis | 14/58 | 139/8661 | 2.15E-13 | 5.92E-12 | 1.59E-12 | TNF/IFNG/BCL2/CCL2/MMP2/HMOX1/MMP9/EDN1/IL1A/NOS3/TNFRSF1A/CTNNB1/FOS/ICAM1 | 14 |
| hsa04668 | TNF signaling pathway | 13/58 | 114/8661 | 3.41E-13 | 8.33E-12 | 2.23E-12 | MAPK1/MAPK3/TNF/CREB1/CCL2/CCL5/MMP9/EDN1/PTGS2/MAP2K1/TNFRSF1A/FOS/ICAM1 | 13 |
| hsa05224 | Breast cancer | 14/58 | 147/8661 | 4.71E-13 | 1.04E-11 | 2.78E-12 | MAPK1/EGF/MAPK3/ESR1/CCND1/PTEN/FGF2/MTOR/IGF1/GSK3B/MAP2K1/EGFR/CTNNB1/FOS | 14 |
| hsa05144 | Malaria | 10/58 | 50/8661 | 6.72E-13 | 1.34E-11 | 3.60E-12 | IL10/TNF/IL18/IFNG/CCL2/CXCL8/TLR4/CSF3/CD36/ICAM1 | 10 |
| hsa01522 | Endocrine resistance | 12/58 | 98/8661 | 1.28E-12 | 2.35E-11 | 6.31E-12 | MAPK1/MAPK3/ESR1/CCND1/BCL2/MMP2/MMP9/MTOR/IGF1/MAP2K1/EGFR/FOS | 12 |
| hsa01521 | EGFR tyrosine kinase inhibitor resistance | 11/58 | 79/8661 | 2.86E-12 | 4.84E-11 | 1.30E-11 | MAPK1/EGF/MAPK3/BCL2/PTEN/FGF2/MTOR/IGF1/GSK3B/MAP2K1/EGFR | 11 |
| hsa05219 | Bladder cancer | 9/58 | 41/8661 | 4.21E-12 | 6.61E-11 | 1.77E-11 | MAPK1/EGF/MAPK3/CCND1/MMP2/CXCL8/MMP9/MAP2K1/EGFR | 9 |
| hsa05210 | Colorectal cancer | 11/58 | 86/8661 | 7.47E-12 | 1.10E-10 | 2.94E-11 | MAPK1/EGF/MAPK3/CCND1/BCL2/MTOR/GSK3B/MAP2K1/EGFR/CTNNB1/FOS | 11 |
| hsa05321 | Inflammatory bowel disease | 10/58 | 65/8661 | 1.09E-11 | 1.49E-10 | 3.99E-11 | IL17A/IL10/TNF/IL18/IFNG/TLR4/IL13/IL1A/IL4/IL2 | 10 |
| hsa05163 | Human cytomegalovirus infection | 15/58 | 225/8661 | 1.15E-11 | 1.49E-10 | 3.99E-11 | MAPK1/MAPK3/TNF/CREB1/CCND1/CCL2/CXCL8/CCL5/PTGS2/MTOR/GSK3B/MAP2K1/EGFR/TNFRSF1A/CTNNB1 | 15 |
| hsa05323 | Rheumatoid arthritis | 11/58 | 93/8661 | 1.80E-11 | 2.20E-10 | 5.89E-11 | IL17A/TNF/IL18/IFNG/CCL2/CXCL8/TLR4/CCL5/IL1A/FOS/ICAM1 | 11 |
| hsa05167 | Kaposi sarcoma-associated herpesvirus infection | 14/58 | 194/8661 | 2.14E-11 | 2.48E-10 | 6.64E-11 | MAPK1/MAPK3/CREB1/CCND1/CXCL8/FGF2/PTGS2/MTOR/GSK3B/MAP2K1/TNFRSF1A/CTNNB1/FOS/ICAM1 | 14 |
| hsa04630 | JAK-STAT signaling pathway | 13/58 | 166/8661 | 4.39E-11 | 4.72E-10 | 1.27E-10 | EGF/GFAP/IL10/CCND1/IFNG/BCL2/MTOR/LEP/IL13/IL4/IL2/CSF3/EGFR | 13 |

# Table S14: Common miRNAs between gastric cancer and depression.

| **No.** | **miRNA Name** | **Associated Diseases** |
| --- | --- | --- |
| 1 | hsa-mir-21 | Gastric cancer & Depression |
| 2 | hsa-mir-146a | Gastric cancer & Depression |
| 3 | hsa-mir-126 | Gastric cancer & Depression |
| 4 | hsa-mir-19b | Gastric cancer & Depression |
| 5 | hsa-mir-34a | Gastric cancer & Depression |
| 6 | hsa-mir-145 | Gastric cancer & Depression |
| 7 | hsa-let-7b | Gastric cancer & Depression |
| 8 | hsa-mir-16 | Gastric cancer & Depression |
| 9 | hsa-let-7a | Gastric cancer & Depression |
| 10 | hsa-mir-26a | Gastric cancer & Depression |
| 11 | hsa-mir-183 | Gastric cancer & Depression |
| 12 | hsa-mir-491 | Gastric cancer & Depression |
