# Supplementary material for: Shared molecular features and candidate pathways underlying gastric cancer–depression comorbidity: a systems biology analysis
Source: Front Bioinform. 2026 May 20;6:1836419. doi: 10.3389/fbinf.2026.1836419 (PMC13231047; doi:10.3389/fbinf.2026.1836419)
Supplement: Supplementary file 3 [file DataSheet1.pdf]

SUPPLEMENTARY MATERIAL

Fig. S1 Pearson correlation analysis identifies outlier samples in the GSE113255 and GSE122401 datasets. **a** Scatter plot of sample pearson correlation in the GSE113255 dataset. The blue cross marks represent outlier samples. **b** Sample clustering dendrogram for the GSE113255 dataset. The red boxes represent the outlier samples. **c** Analysis of average connectivity at different soft threshold powers based on dataset GSE113255. **d** Scatter plot of sample pearson correlation in the GSE122401 dataset. The blue cross marks represent outlier samples. **e** Sample clustering dendrogram for the GSE122401 dataset. The red boxes represent the outlier samples. **f** Analysis of average connectivity at different soft threshold powers based on dataset GSE122401.

Fig. S2 Top 20 genes identified by the MCC algorithm using the cytoHubba plugin. **a** Top 20 genes obtained by the MCC algorithm in GC. **b** Top 20 genes obtained by the MCC algorithm in depression. GC, gastric cancer; MCC, maximal clique centrality.

Fig. S3 Kaplan-Meier survival analysis of 6 hub genes based on TCGA-STAD cohort in GEPIA2 database. **a** Survival analysis for SERPINE1 in STAD. **b** Survival analysis for COL4A1 in STAD. **c** Survival analysis for PDGFRB in STAD. **d** Survival analysis for BMP1 in STAD. **e** Survival analysis for NOTCH3 in STAD. **f** Survival analysis for EDNRA in STAD.

Fig. S4 GSEA of hub genes in datasets GSE113255, showing top five enriched pathways. **a** GSEA of SERPINE1. **b** GSEA of COL4A1. **c** GSEA of PDGFRB. **d** GSEA of BMP1. **e** GSEA of

NOTCH3. **f** GSEA of EDNRA. GSEA, gene set enrichment analysis.

Fig. S5 GSEA of hub genes in datasets GSE122401, showing top five enriched pathways. **a** GSEA of SERPINE1. **b** GSEA of COL4A1. **c** GSEA of PDGFRB. **d** GSEA of BMP1. **e** GSEA of NOTCH3. **f** GSEA of EDNRA. GSEA, gene set enrichment analysis.

Fig. S6 Analysis of immune cell infiltration and correlation with hub genes in the GEO dataset. **a** Differences in immune cells between GC tissues and normal tissues in GSE113255. **b** Analysis of the association of 6 hub genes with immune cells in GSE113255. **c** Differences in immune cells between GC tissues and normal tissues in GSE122401. **d** Analysis of the association of 6 hub genes with immune cells in GSE122401. GEO, Gene Expression Omnibus. **e** Differences in immune cells between depression and healthy controls in GSE54575. **f** Analysis of the association of 6 hub genes with immune cells in GSE54575. GEO, Gene Expression Omnibus.

S1

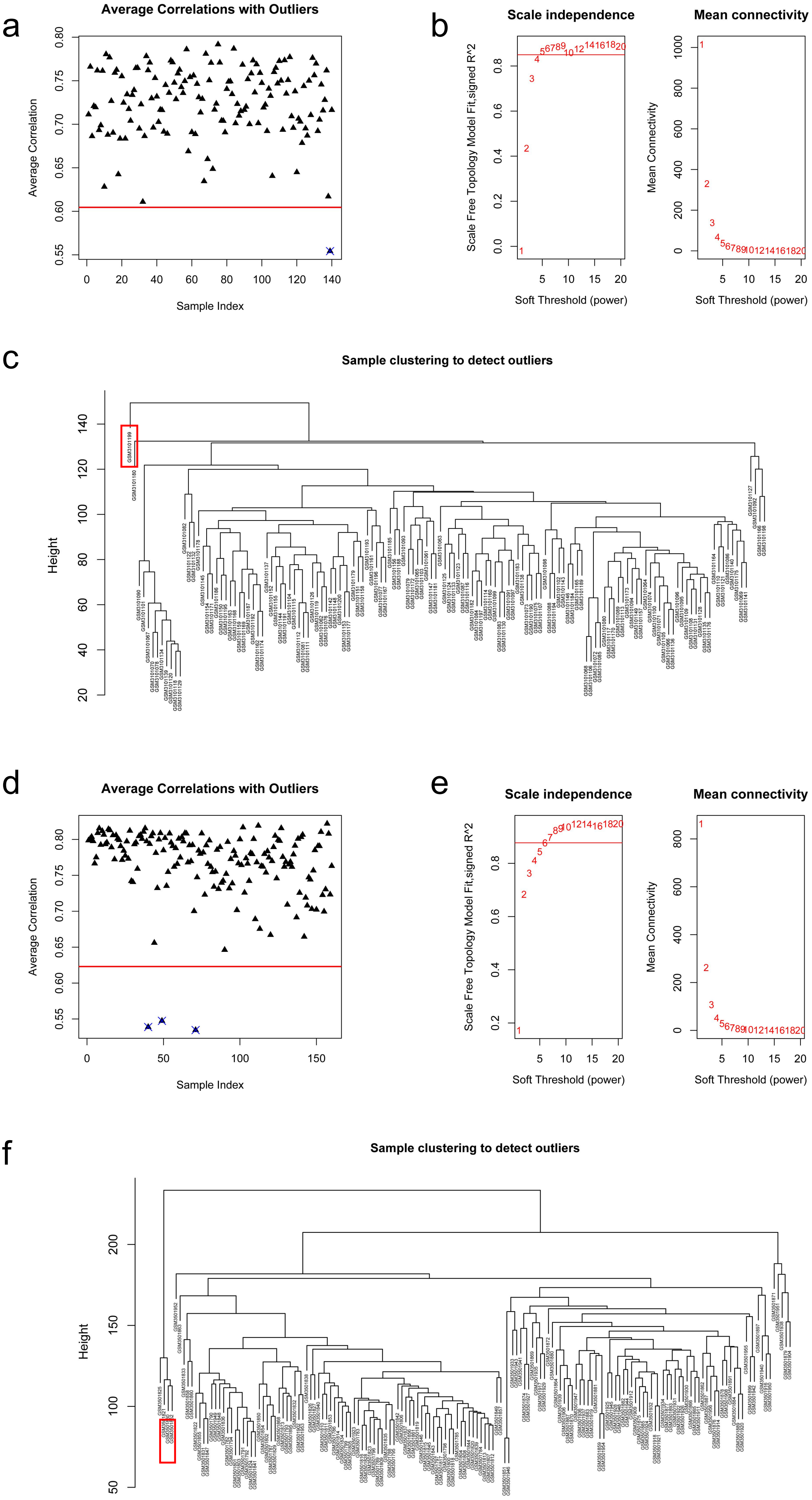

a
Average Correlations with Outliers
Average Correlation
Sample Index
b
Scale independence
Scale Free Topology Model Fit,signed R^2
Soft Threshold (power)
Mean connectivity
Mean Connectivity
Soft Threshold (power)
c
Sample clustering to detect outliers
Height
d
Average Correlations with Outliers
Average Correlation
Sample Index
e
Scale independence
Scale Free Topology Model Fit,signed R^2
Soft Threshold (power)
Mean connectivity
Mean Connectivity
Soft Threshold (power)
f
Sample clustering to detect outliers
Height

S2

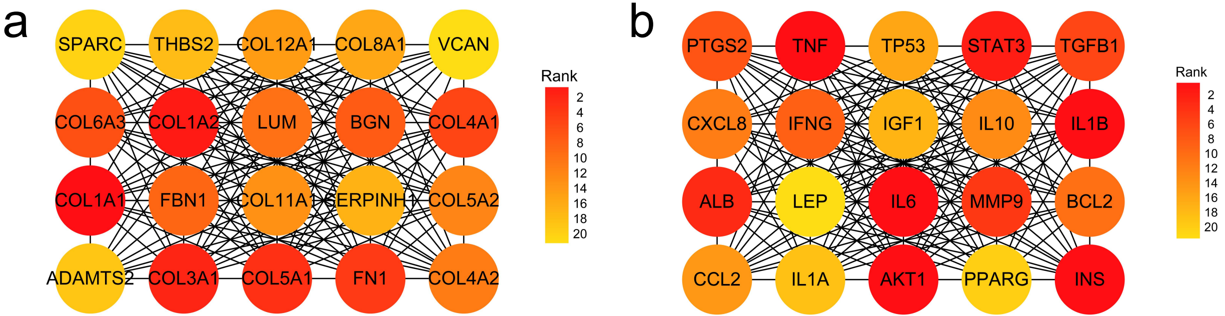
a
SPARC
THBS2
COL12A1
COL8A1
VCAN
COL6A3
COL1A2
LUM
BGN
COL4A1
COL1A1
FBN1
COL11A1
SERPINH1
COL5A2
ADAMTS2
COL3A1
COL5A1
FN1
COL4A2
Rank
2
4
6
8
10
12
14
16
18
20
b
PTGS2
TNF
TP53
STAT3
TGFB1
CXCL8
IFNG
IGF1
IL10
IL1B
ALB
LEP
IL6
MMP9
BCL2
CCL2
IL1A
AKT1
PPARG
INS
Rank
2
4
6
8
10
12
14
16
18
20

S3

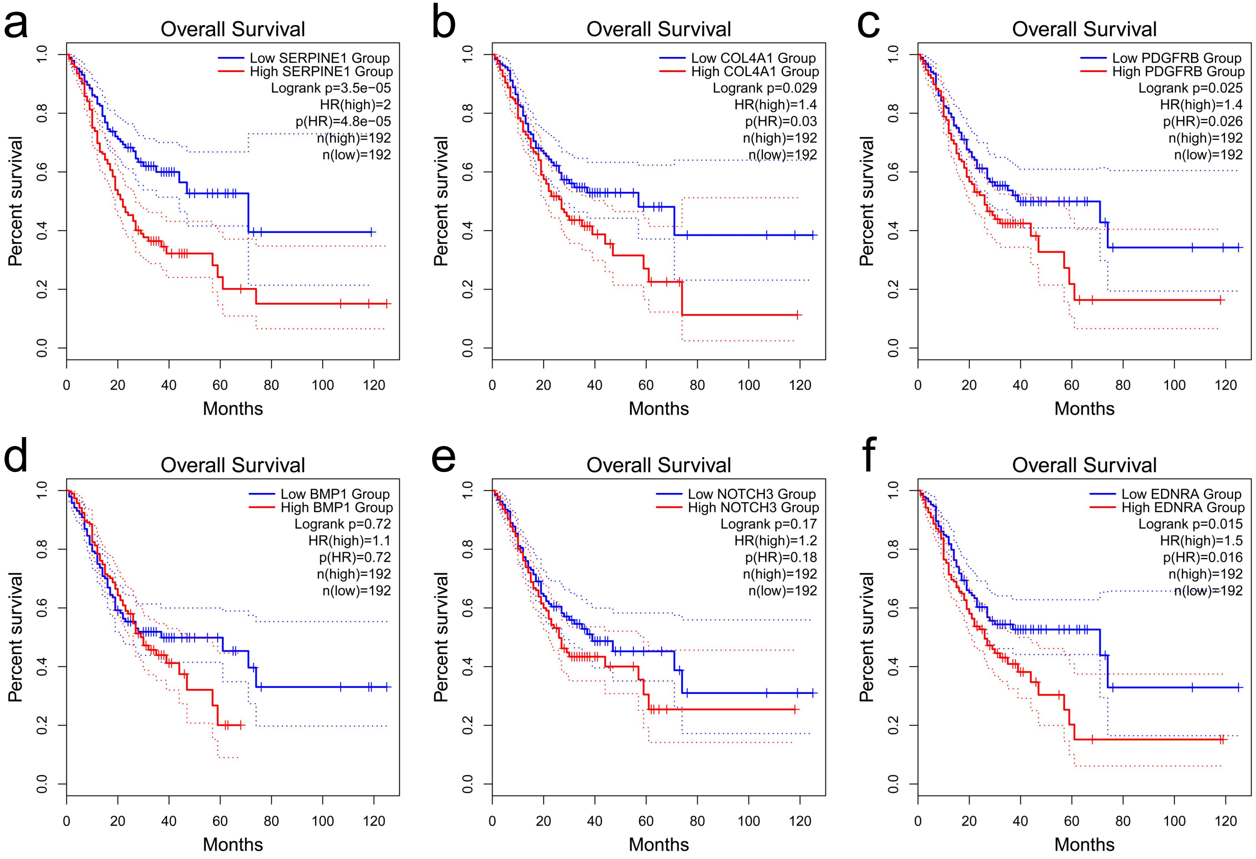

a
Overall Survival
Low SERPINE1 Group
High SERPINE1 Group
Logrank p=3.5e−05
HR(high)=2
p(HR)=4.8e−05
n(high)=192
n(low)=192
Percent survival
Months
b
Overall Survival
Low COL4A1 Group
High COL4A1 Group
Logrank p=0.029
HR(high)=1.4
p(HR)=0.03
n(high)=192
n(low)=192
Percent survival
Months
c
Overall Survival
Low PDGFRB Group
High PDGFRB Group
Logrank p=0.025
HR(high)=1.4
p(HR)=0.026
n(high)=192
n(low)=192
Percent survival
Months
d
Overall Survival
Low BMP1 Group
High BMP1 Group
Logrank p=0.72
HR(high)=1.1
p(HR)=0.72
n(high)=192
n(low)=192
Percent survival
Months
e
Overall Survival
Low NOTCH3 Group
High NOTCH3 Group
Logrank p=0.17
HR(high)=1.2
p(HR)=0.18
n(high)=192
n(low)=192
Percent survival
Months
f
Overall Survival
Low EDNRA Group
High EDNRA Group
Logrank p=0.015
HR(high)=1.5
p(HR)=0.016
n(high)=192
n(low)=192
Percent survival
Months

S4

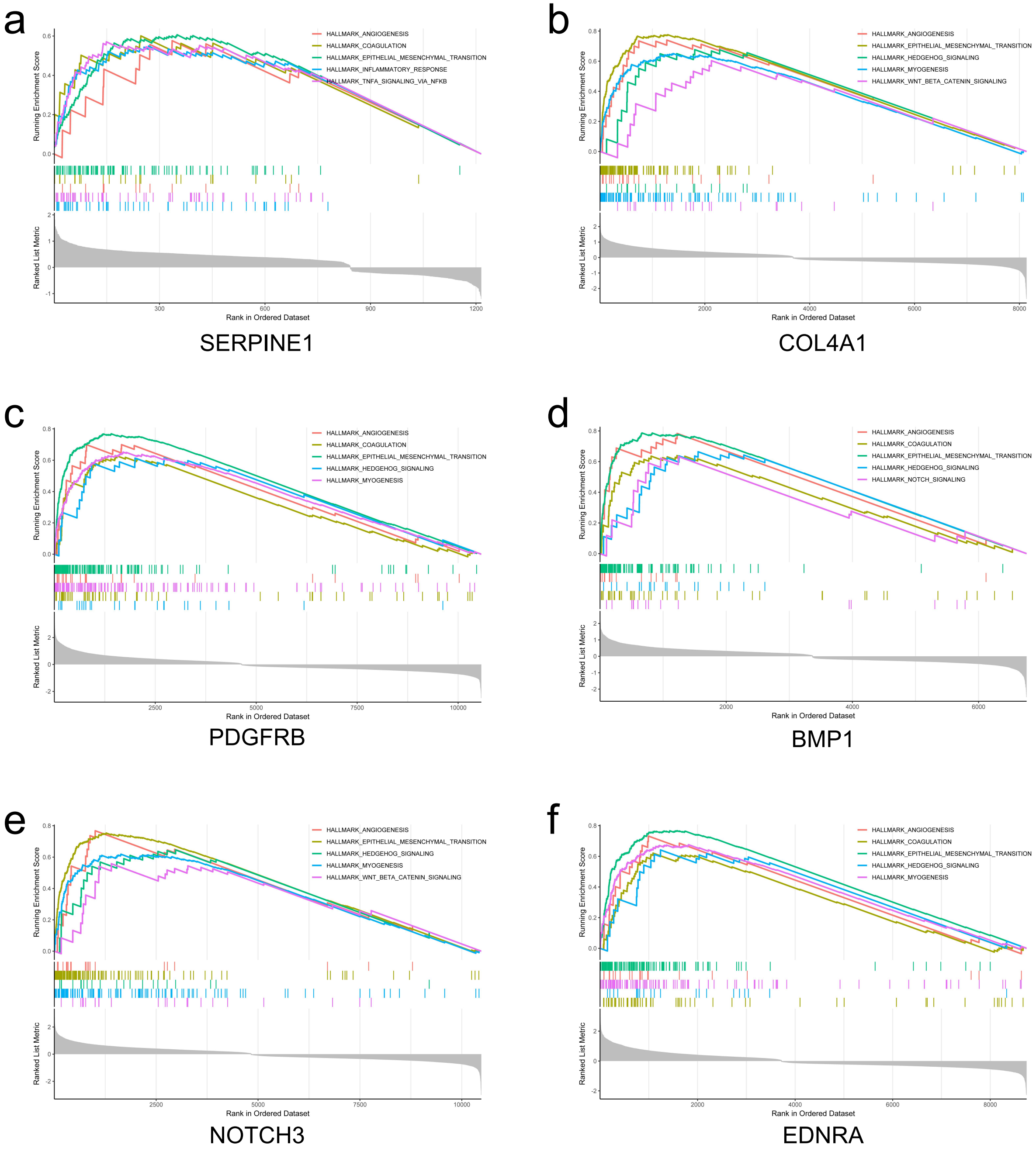
a
HALLMARK_ANGIOGENESIS
HALLMARK_COAGULATION
HALLMARK_EPITHELIAL_MESENCHYMAL_TRANSITION
HALLMARK_INFLAMMATORY_RESPONSE
HALLMARK_TNFA_SIGNALING_VIA_NFKB
Running Enrichment Score
Ranked List Metric
Rank in Ordered Dataset
SERPINE1
b
HALLMARK_ANGIOGENESIS
HALLMARK_EPITHELIAL_MESENCHYMAL_TRANSITION
HALLMARK_HEDGEHOG_SIGNALING
HALLMARK_MYOGENESIS
HALLMARK_WNT_BETA_CATENIN_SIGNALING
Running Enrichment Score
Ranked List Metric
Rank in Ordered Dataset
COL4A1
c
HALLMARK_ANGIOGENESIS
HALLMARK_COAGULATION
HALLMARK_EPITHELIAL_MESENCHYMAL_TRANSITION
HALLMARK_HEDGEHOG_SIGNALING
HALLMARK_MYOGENESIS
Running Enrichment Score
Ranked List Metric
Rank in Ordered Dataset
PDGFRB
d
HALLMARK_ANGIOGENESIS
HALLMARK_COAGULATION
HALLMARK_EPITHELIAL_MESENCHYMAL_TRANSITION
HALLMARK_HEDGEHOG_SIGNALING
HALLMARK_NOTCH_SIGNALING
Running Enrichment Score
Ranked List Metric
Rank in Ordered Dataset
BMP1
e
HALLMARK_ANGIOGENESIS
HALLMARK_EPITHELIAL_MESENCHYMAL_TRANSITION
HALLMARK_HEDGEHOG_SIGNALING
HALLMARK_MYOGENESIS
HALLMARK_WNT_BETA_CATENIN_SIGNALING
Running Enrichment Score
Ranked List Metric
Rank in Ordered Dataset
NOTCH3
f
HALLMARK_ANGIOGENESIS
HALLMARK_COAGULATION
HALLMARK_EPITHELIAL_MESENCHYMAL_TRANSITION
HALLMARK_HEDGEHOG_SIGNALING
HALLMARK_MYOGENESIS
Running Enrichment Score
Ranked List Metric
Rank in Ordered Dataset
EDNRA

S5

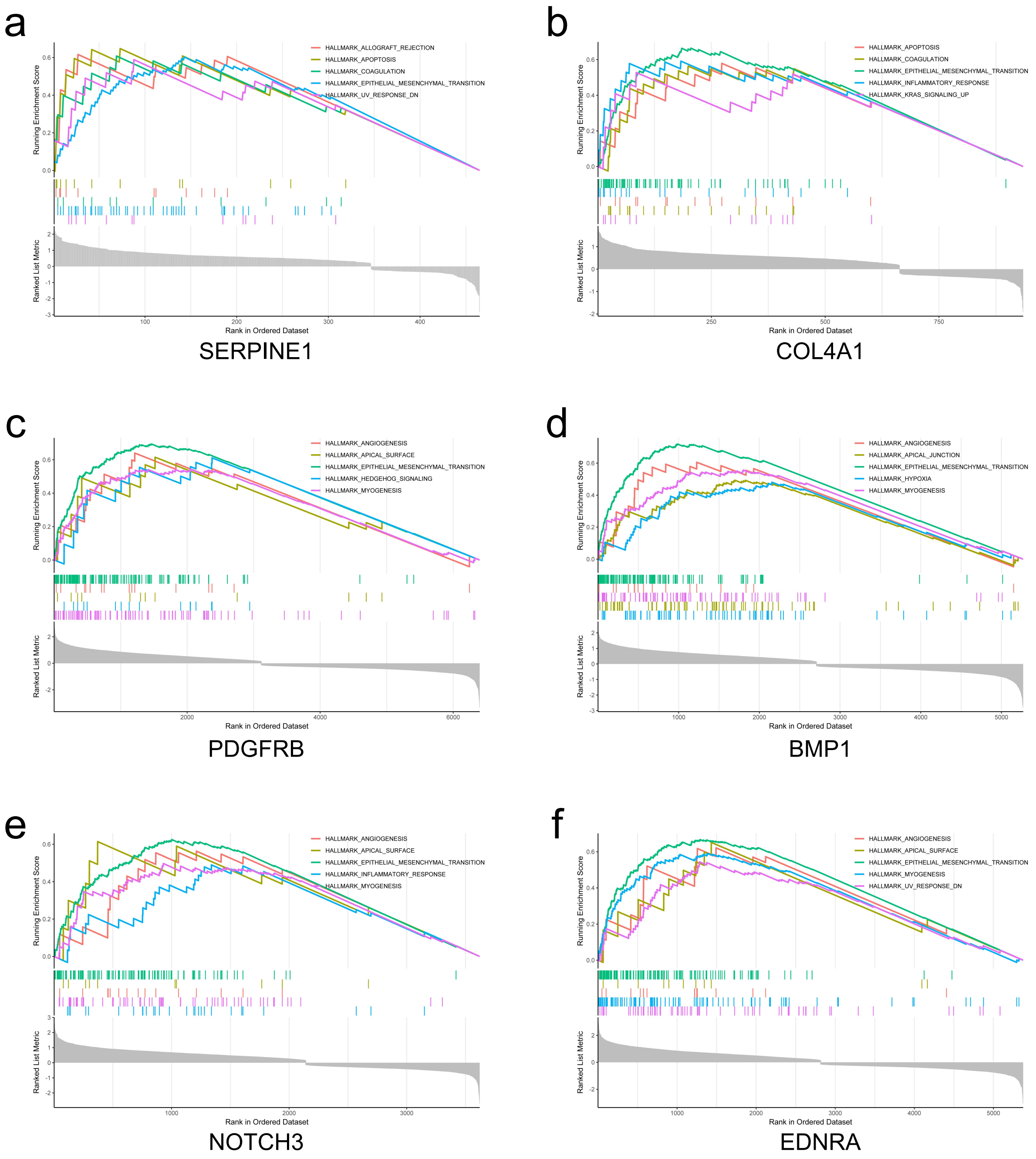
a
HALLMARK_ALLOGRAFT_REJECTION
HALLMARK_APOPTOSIS
HALLMARK_COAGULATION
HALLMARK_EPITHELIAL_MESENCHYMAL_TRANSITION
HALLMARK_UV_RESPONSE_DN
Running Enrichment Score
Ranked List Metric
Rank in Ordered Dataset
SERPINE1
b
HALLMARK_APOPTOSIS
HALLMARK_COAGULATION
HALLMARK_EPITHELIAL_MESENCHYMAL_TRANSITION
HALLMARK_INFLAMMATORY_RESPONSE
HALLMARK_KRAS_SIGNALING_UP
Running Enrichment Score
Ranked List Metric
Rank in Ordered Dataset
COL4A1
c
HALLMARK_ANGIOGENESIS
HALLMARK_APICAL_SURFACE
HALLMARK_EPITHELIAL_MESENCHYMAL_TRANSITION
HALLMARK_HEDGEHOG_SIGNALING
HALLMARK_MYOGENESIS
Running Enrichment Score
Ranked List Metric
Rank in Ordered Dataset
PDGFRB
d
HALLMARK_ANGIOGENESIS
HALLMARK_APICAL_JUNCTION
HALLMARK_EPITHELIAL_MESENCHYMAL_TRANSITION
HALLMARK_HYPOXIA
HALLMARK_MYOGENESIS
Running Enrichment Score
Ranked List Metric
Rank in Ordered Dataset
BMP1
e
HALLMARK_ANGIOGENESIS
HALLMARK_APICAL_SURFACE
HALLMARK_EPITHELIAL_MESENCHYMAL_TRANSITION
HALLMARK_INFLAMMATORY_RESPONSE
HALLMARK_MYOGENESIS
Running Enrichment Score
Ranked List Metric
Rank in Ordered Dataset
NOTCH3
f
HALLMARK_ANGIOGENESIS
HALLMARK_APICAL_SURFACE
HALLMARK_EPITHELIAL_MESENCHYMAL_TRANSITION
HALLMARK_MYOGENESIS
HALLMARK_UV_RESPONSE_DN
Running Enrichment Score
Ranked List Metric
Rank in Ordered Dataset
EDNRA

S6

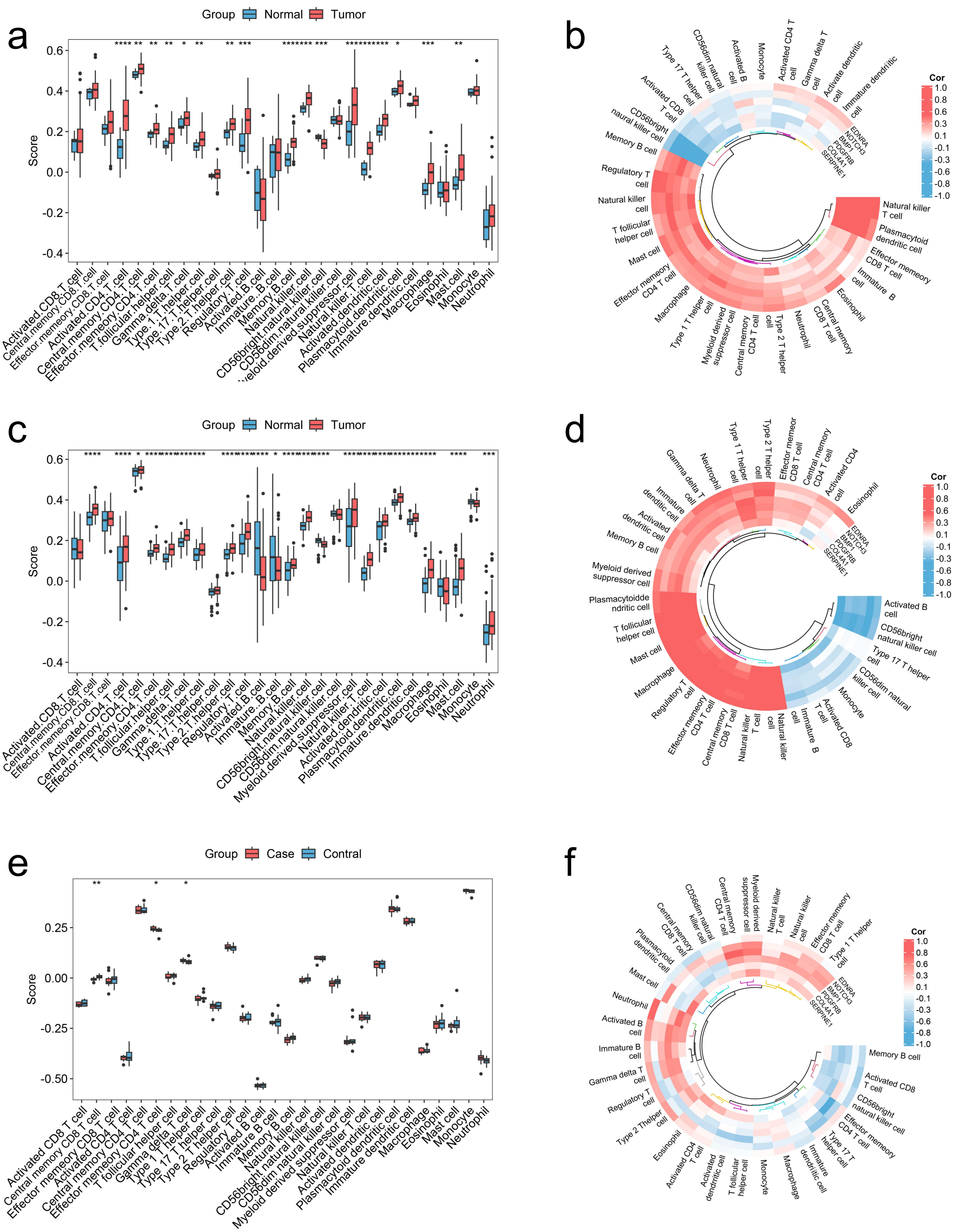
a
Group Normal Tumor
Score
0.6
0.4
0.2
0.0
-0.2
-0.4
Activated.CD8.T.cell
Central.memory.CD8.T.cell
Effector.memeory.CD8.T.cell
Activated.CD4.T.cell
Central.memory.CD4.T.cell
Effector.memeory.CD4.T.cell
T.follicular.helper.cell
Gamma.delta.T.cell
Type.1.T.helper.cell
Type.17.T.helper.cell
Type.2.T.helper.cell
Regulatory.T.cell
Activated.B.cell
Immature..B.cell
Memory.B.cell
Natural.killer.cell
CD56bright.natural.killer.cell
CD56dim.natural.killer.cell
Myeloid.derived.suppressor.cell
Natural.killer.T.cell
Activated.dendritic.cell
Plasmacytoid.dendritic.cell
Immature.dendritic.cell
Macrophage
Eosinophil
Mast.cell
Monocyte
Neutrophil
b
Cor
1.0
0.8
0.6
0.3
0.1
-0.1
-0.3
-0.6
-0.8
-1.0
EDNRA
NOTCH3
BMP1
PDGFRB
COL4A1
SERPINE1
c
Group Normal Tumor
Score
d
Cor
e
Group Case Contral
Score
0.25
0.00
-0.25
-0.50
f
Cor
