# Supplementary material for: Shared molecular features and candidate pathways underlying gastric cancer–depression comorbidity: a systems biology analysis
Source: Front Bioinform. 2026 May 20;6:1836419. doi: 10.3389/fbinf.2026.1836419 (PMC13231047; doi:10.3389/fbinf.2026.1836419)
Supplement: Supplementary file 4 [file Supplementaryfile2.docx]

BMP1 Sample1-6 BMP1 Sample7-12


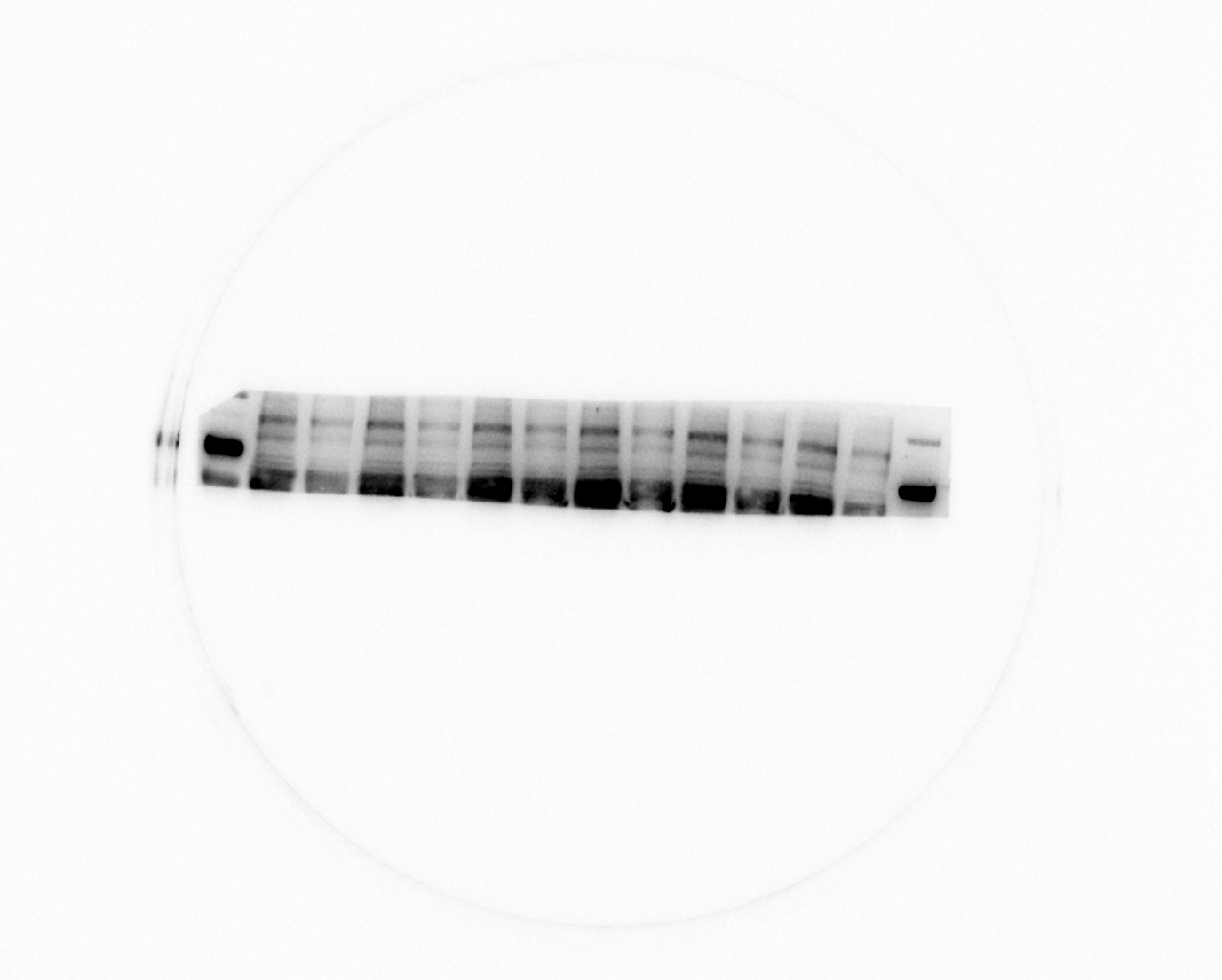

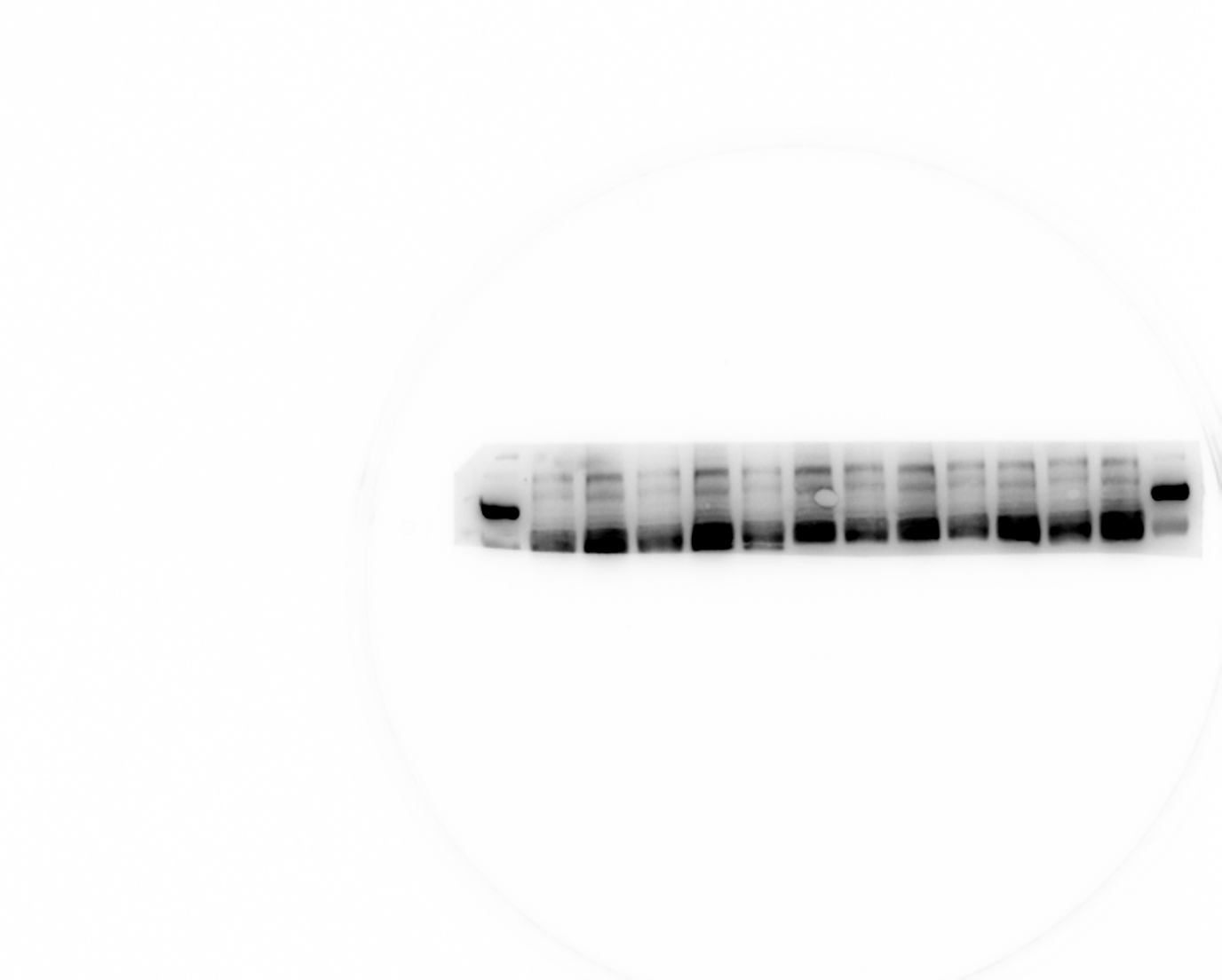


EDNRA Sample1-6 EDNRA Sample7-12


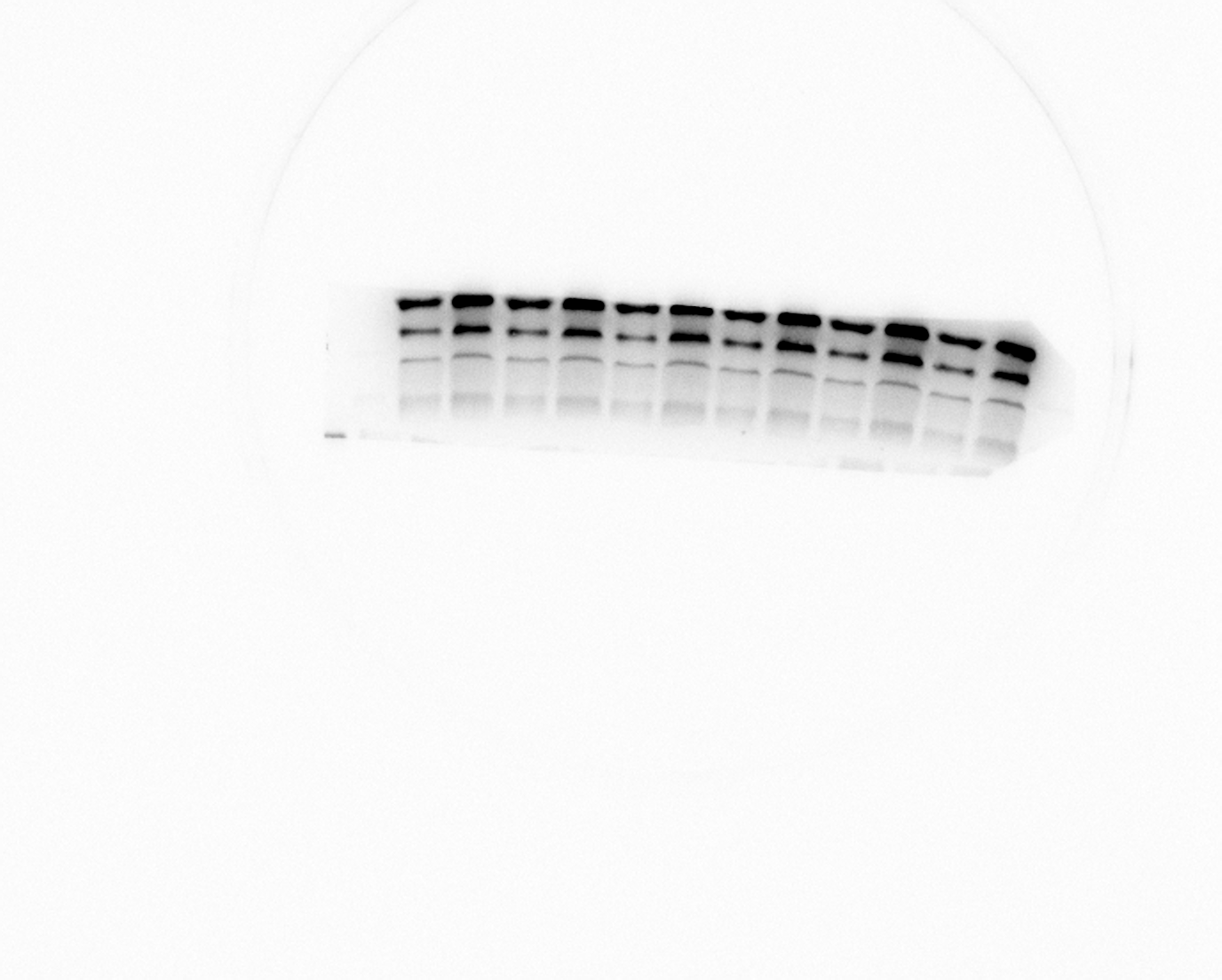
**
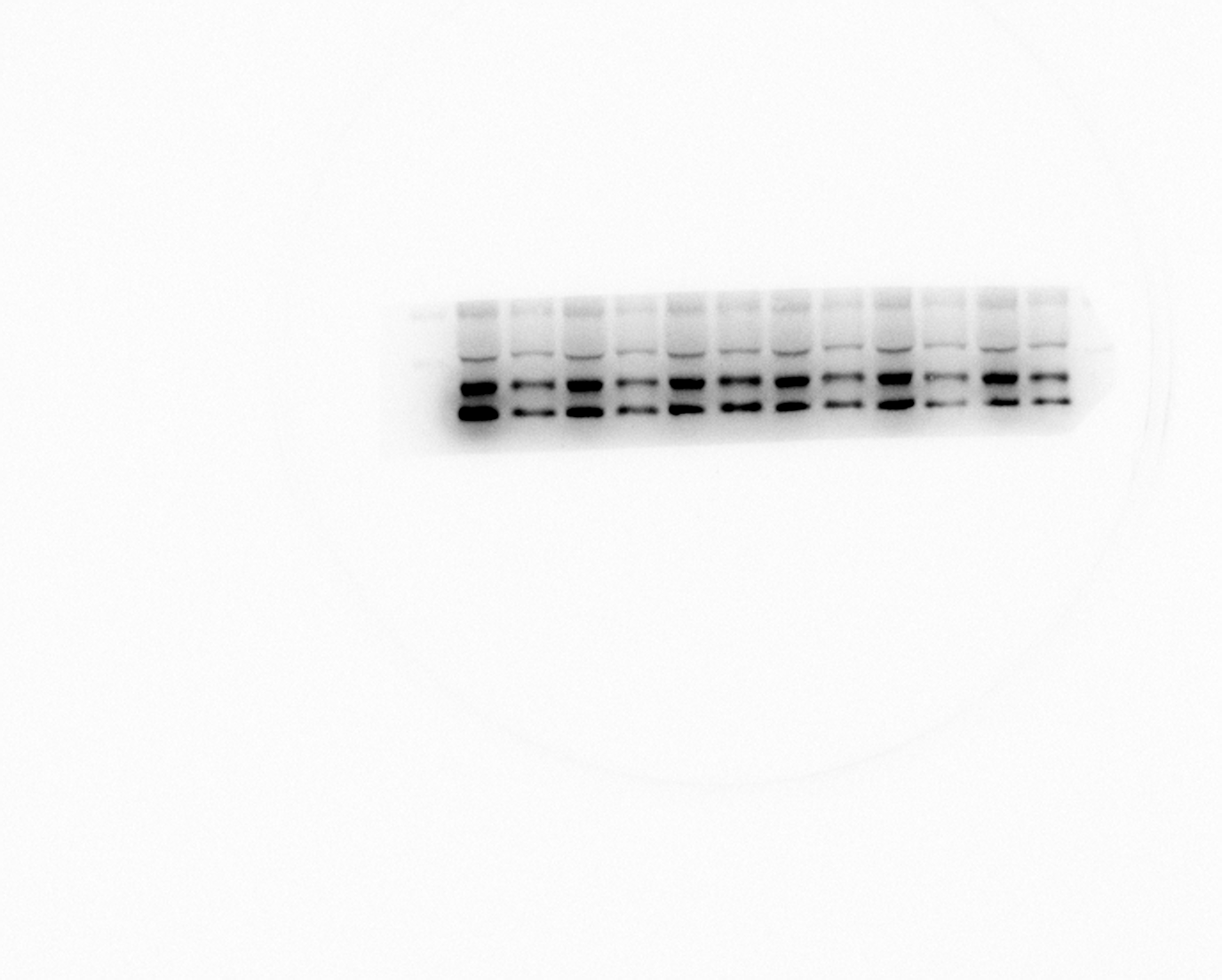
**

SERPINE1 Sample1-6 SERPINE1 Sample7-12

**
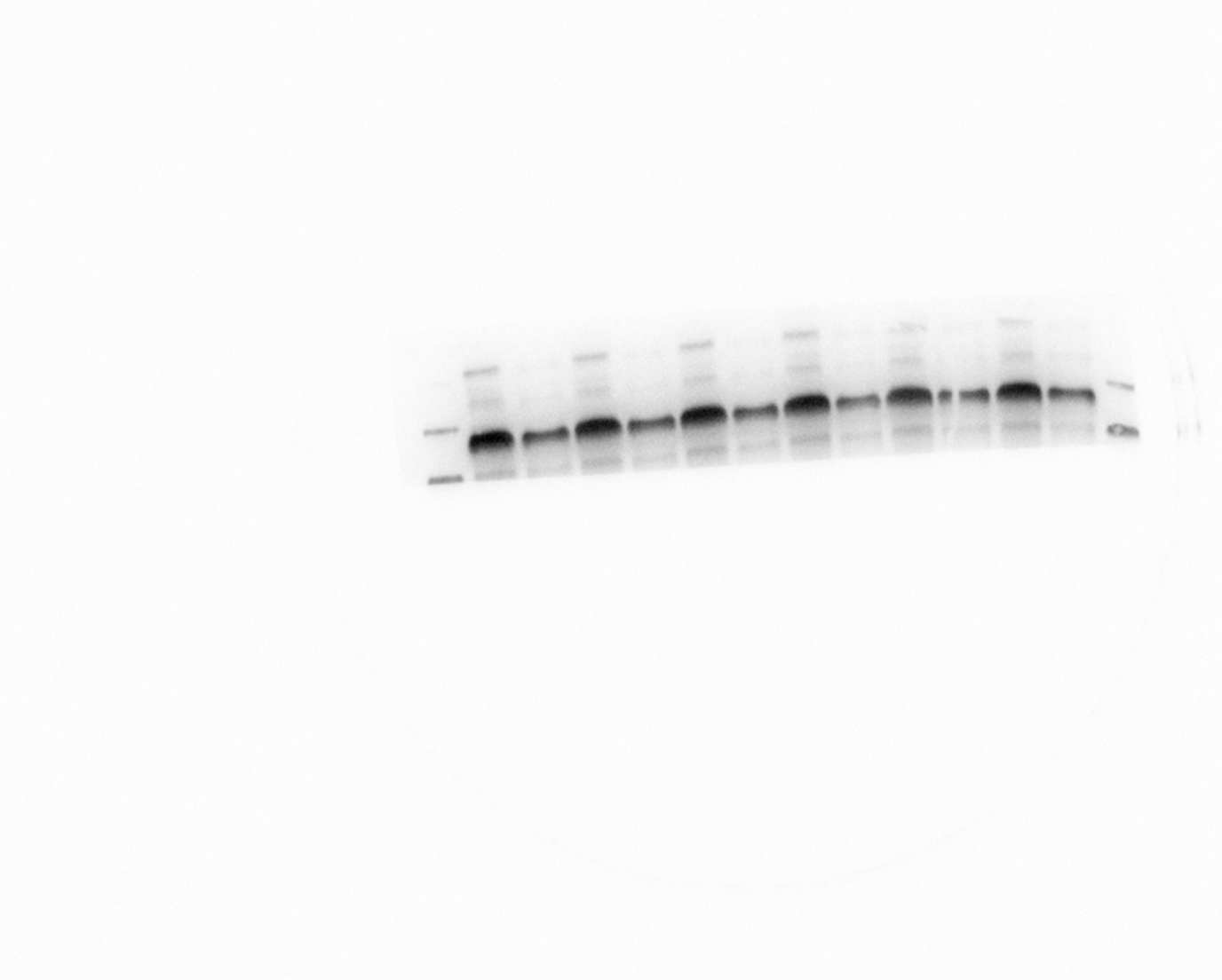

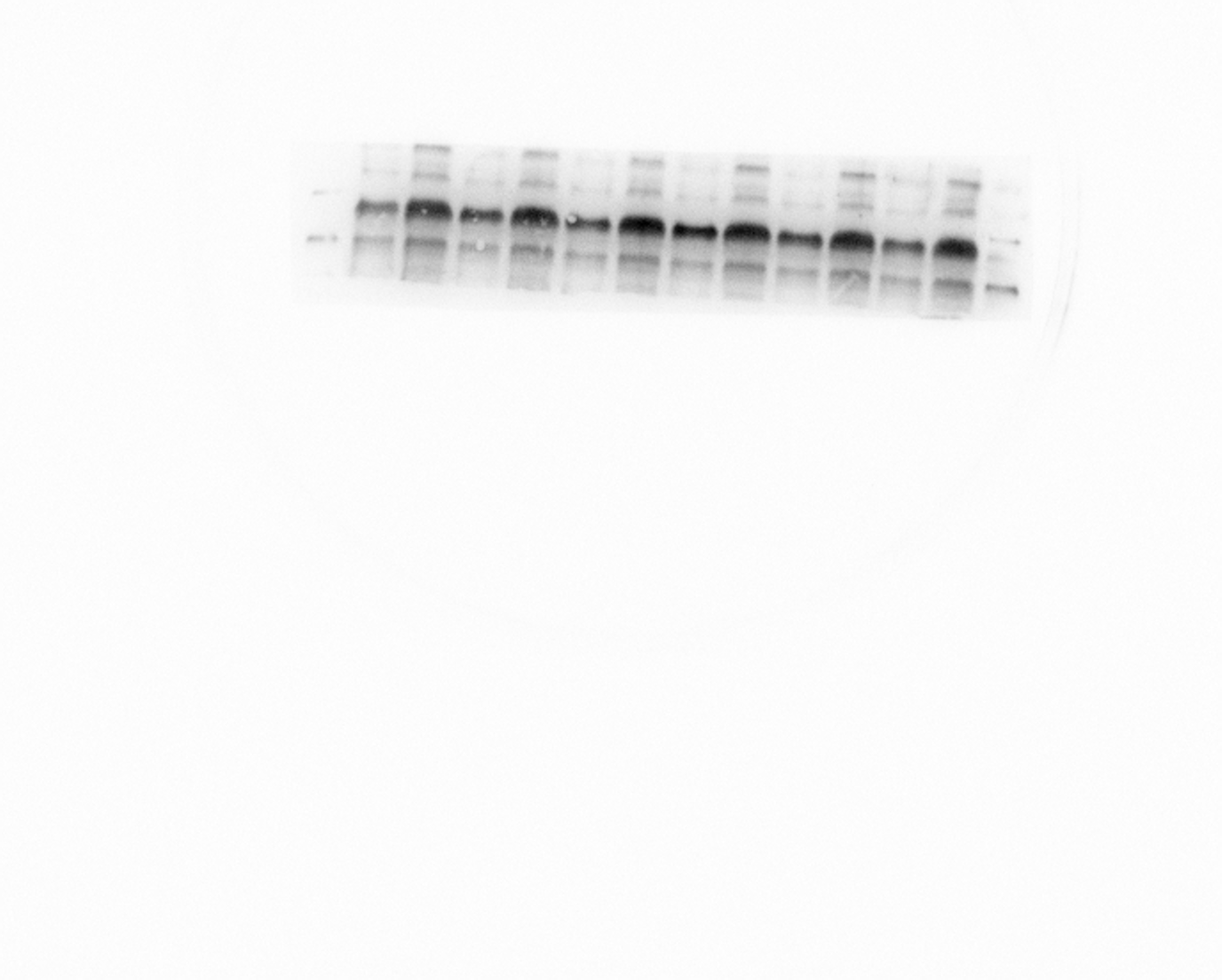
**

PDGFRB Sample1-6 PDGFRB Sample7-12

**
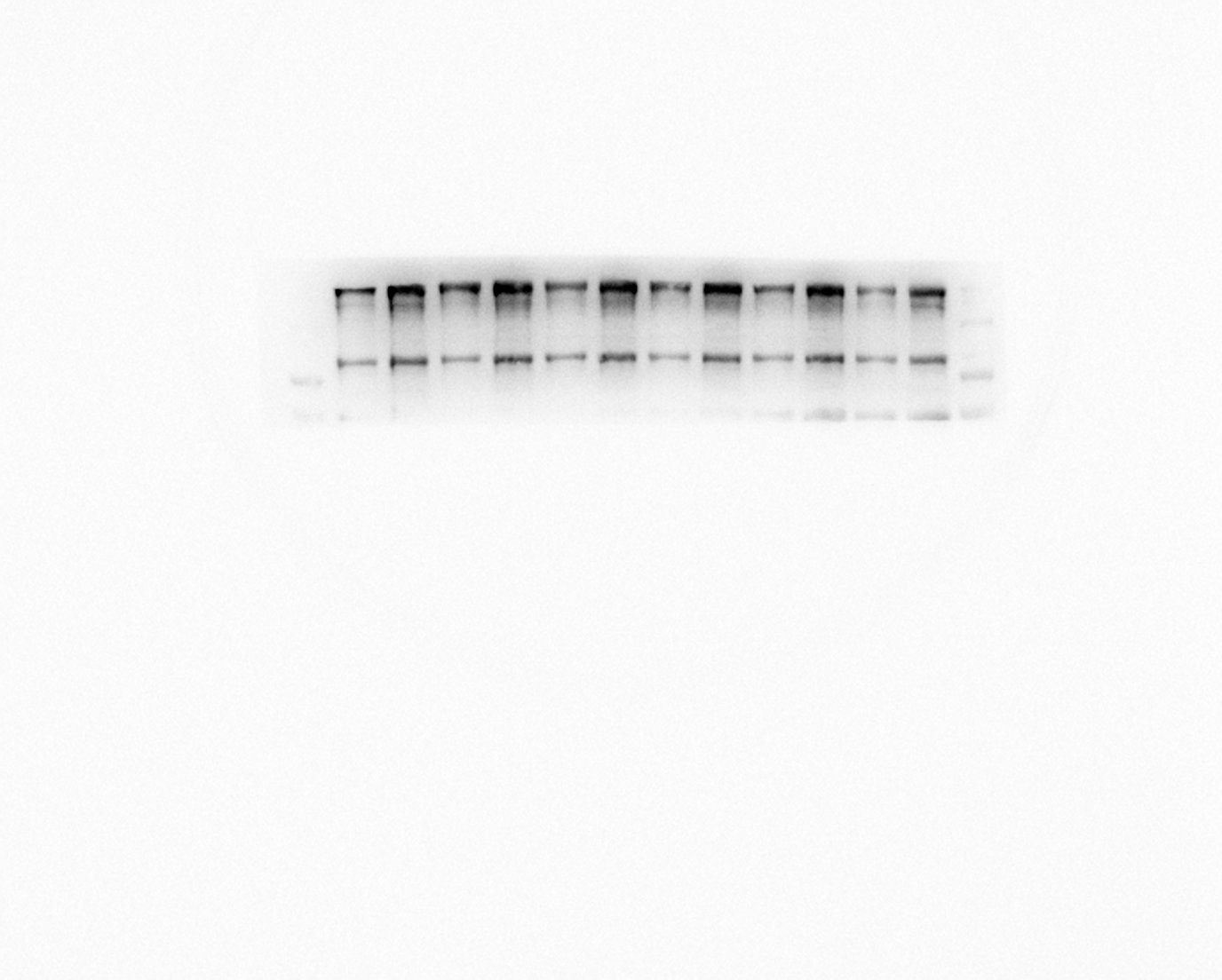

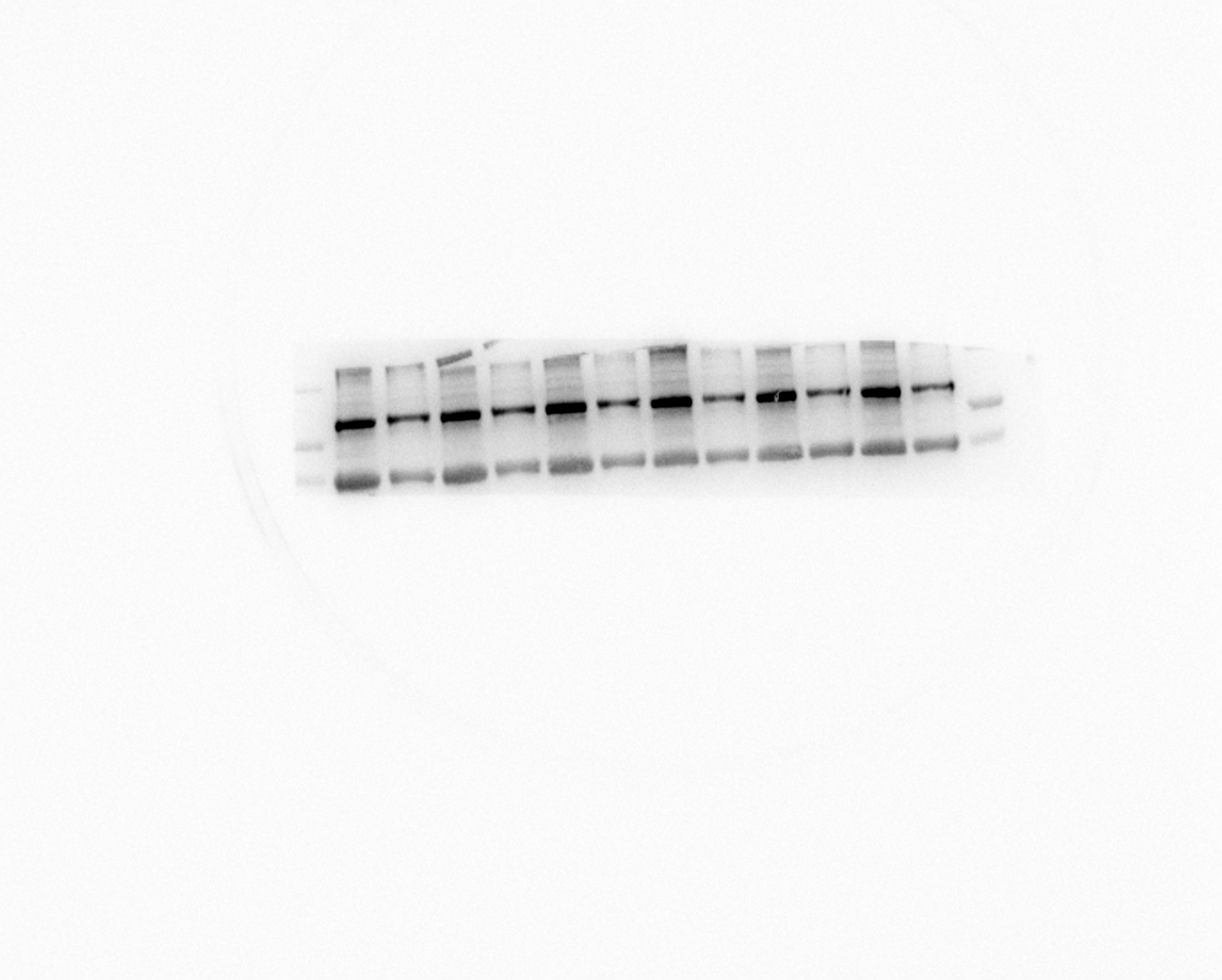
**

COL4A1 Sample1-6 COL4A1 Sample7-12

**
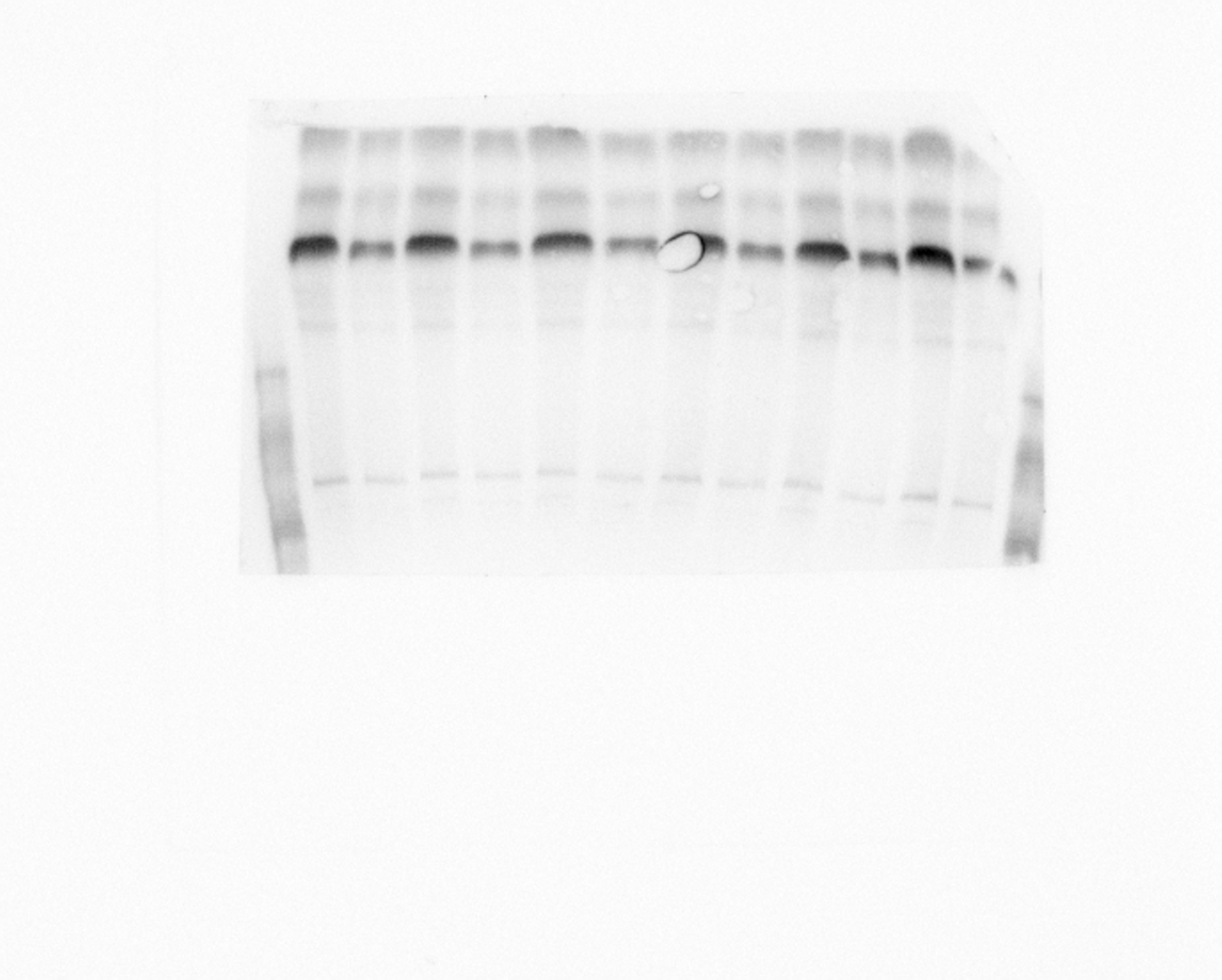

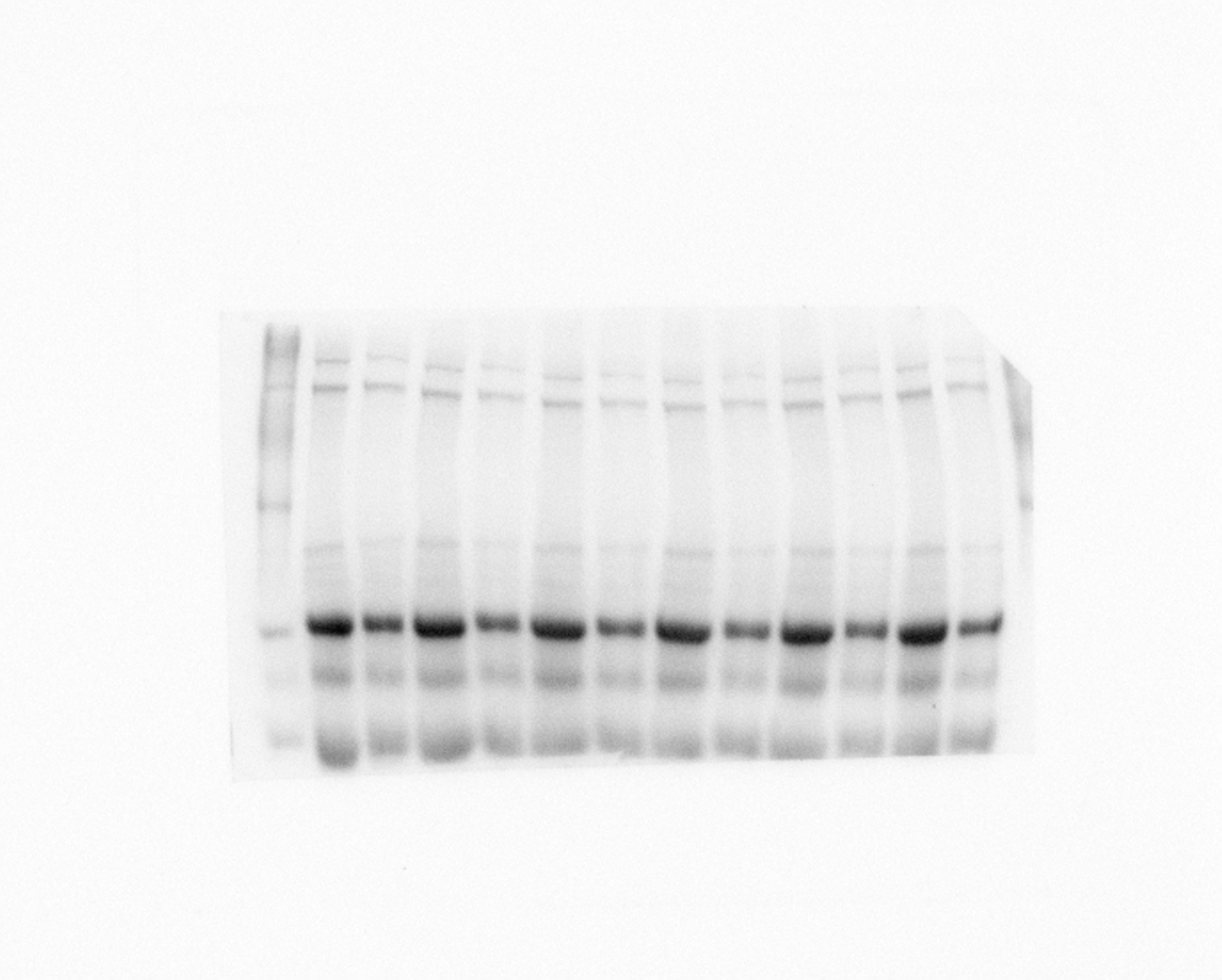
**

GAPDH Sample1-6 GAPDH Sample7-12

**
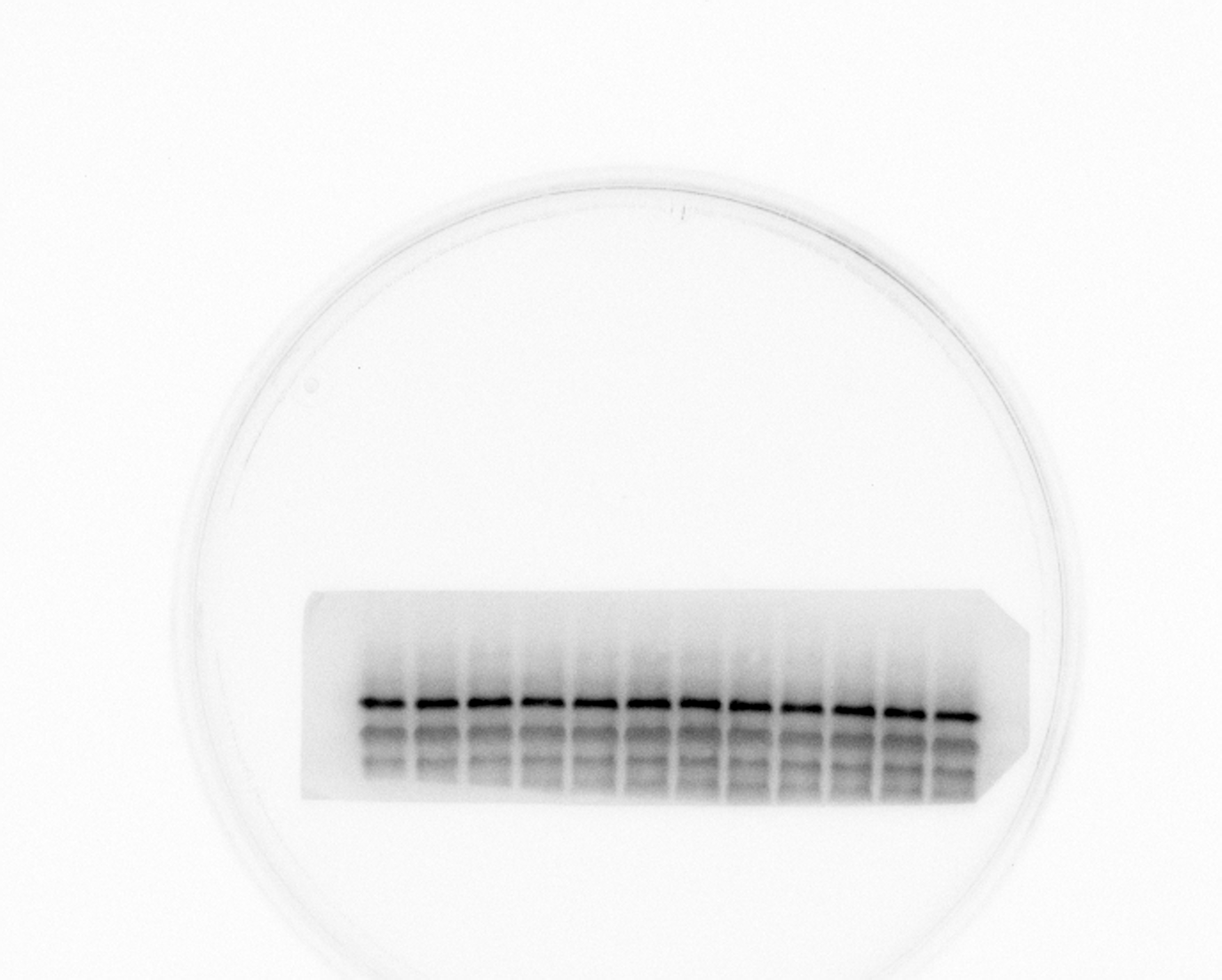

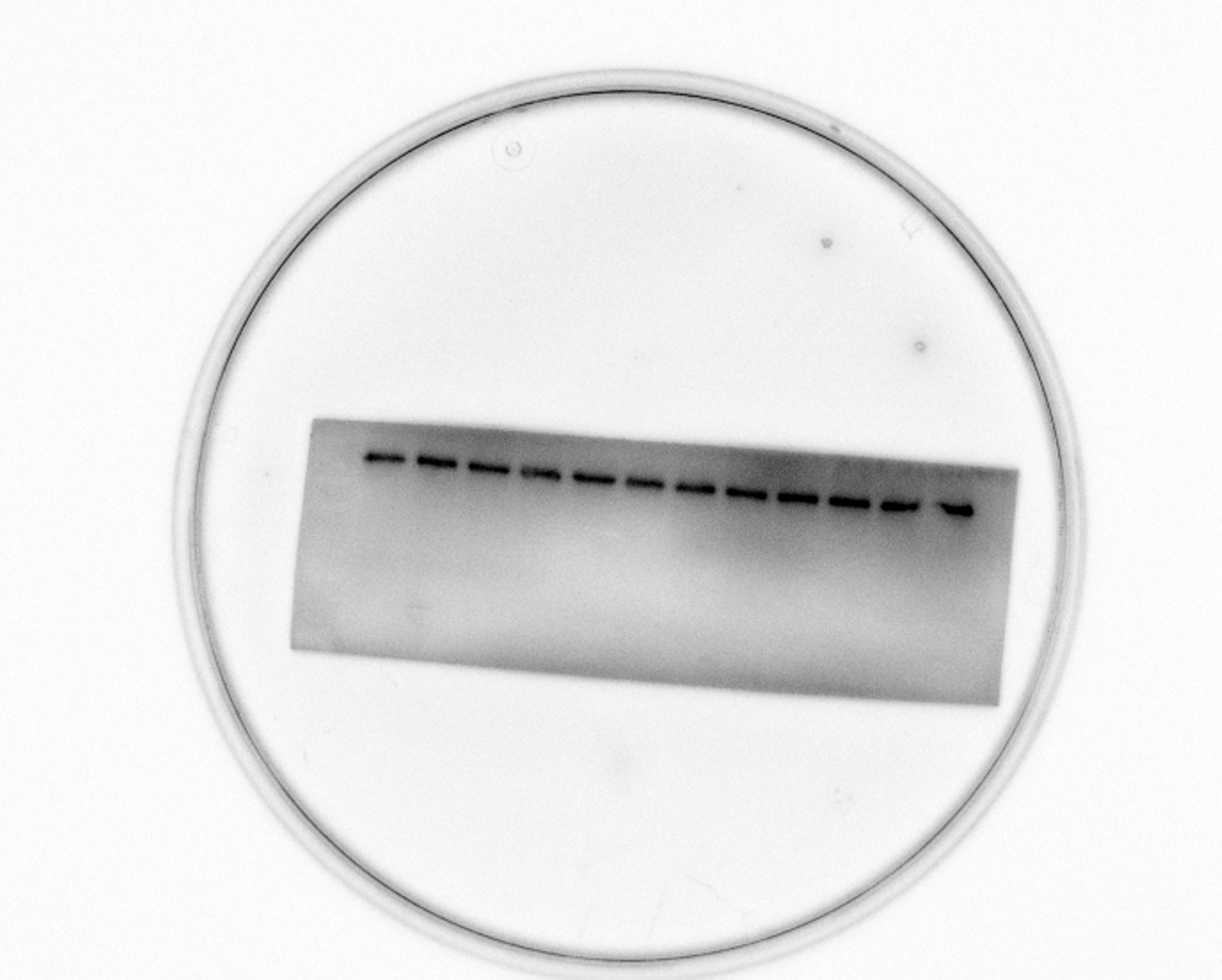
**

NOTCH3 Sample1-6 NOTCH3 Sample7-12

**
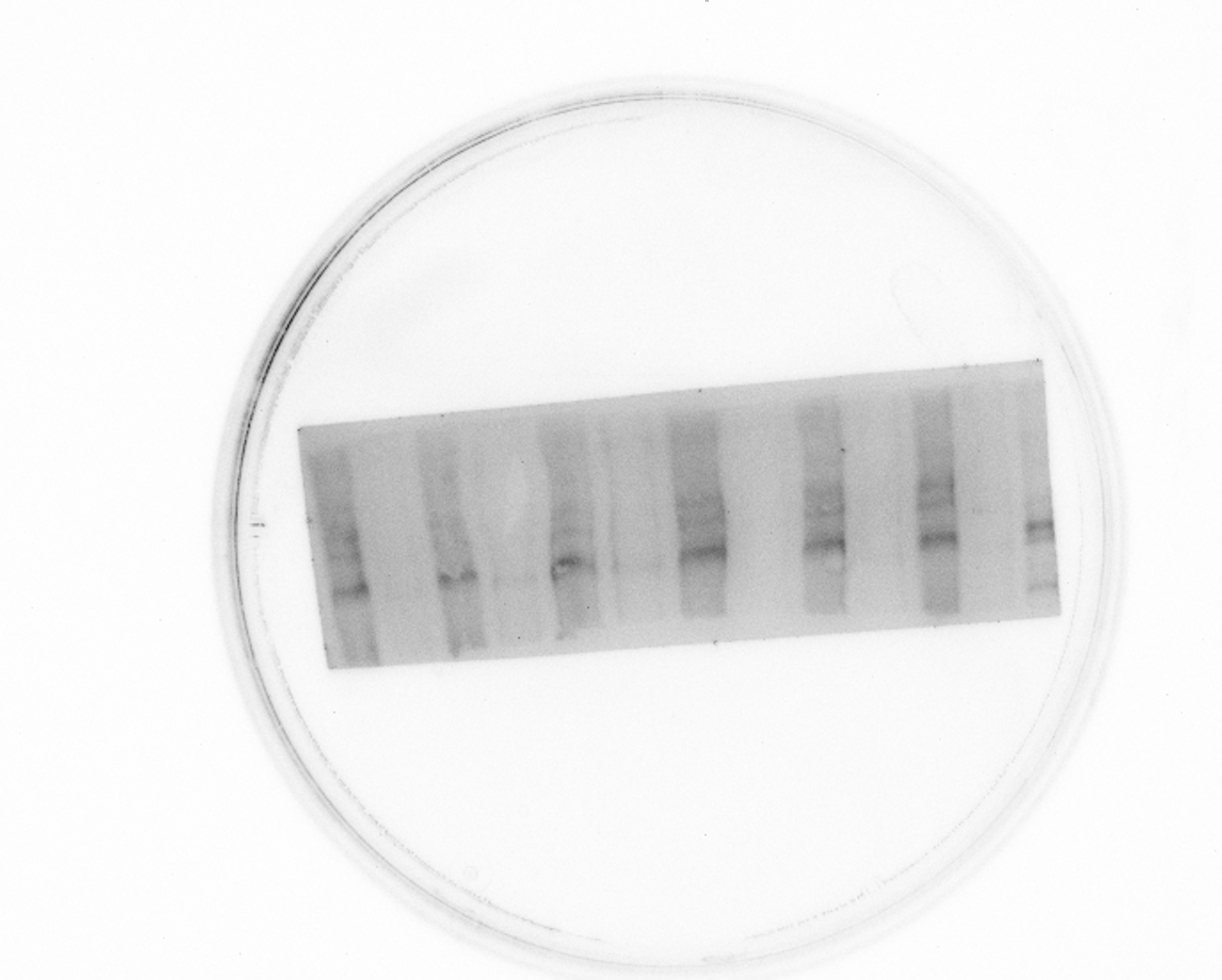

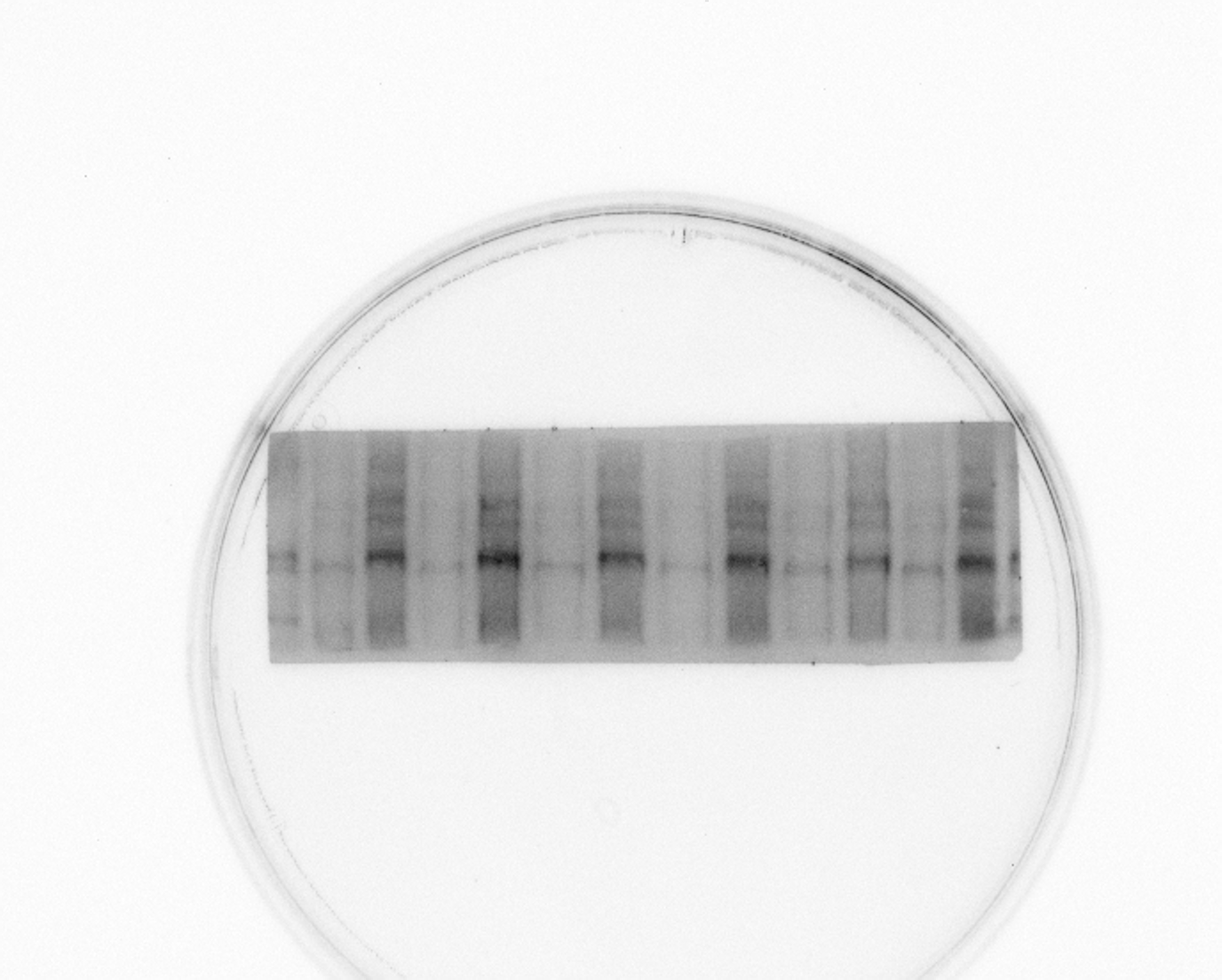
**
